# A Guide to the Medical School Curriculum Vitae

**DOI:** 10.21980/J8HH1S

**Published:** 2024-01-31

**Authors:** Konnor Davis, Megan Boysen-Osborn, Alisa Wray, Lauren Stokes

**Affiliations:** * University of California, Irvine, School of Medicine, Irvine, CA; ^ University of California, Irvine, Department of Emergency Medicine, Orange, CA

## Abstract

**Audience:**

Although this lecture is aimed at medical students, it can also be utilized for residents, fellows, and junior faculty.

**Background:**

The topic of teaching medical students about the fundamentals of creating a curriculum vitae (CV) is important because a CV serves as a record of scholastic and professional experiences.^1^ Thus, their CV will undoubtedly play a vital role in residency applications.^2^,^3^ Intentional instruction about the elements to incorporate in a CV are especially important for first-generation and underrepresented students in the medical field because they may not have had as much exposure to both the requirements of a residency application nor qualities of an effective CV.

**Educational Objectives:**

After this lecture, learners should be able to: 1) elaborate on the significance of a CV for medical students and discuss its purpose, 2) outline the elements that should and should not be included on a CV, 3) integrate knowledge gleaned from basic principles with provided examples to establish the foundation of their own CV.

**Educational Methods:**

A PowerPoint lecture was used to explain the purpose of a CV and the elements to include in a personal CV for medical students. The lecture took place via Zoom and was provided at no cost to all UCISOM medical students.

**Research Methods:**

Students were given a short survey after the session to assess their understanding of why it is important to create and maintain a CV, including an evaluation of their overall satisfaction with the lecture presentation.

**Results:**

All the respondents (n=10) found the workshop to be useful and enjoyed the ability to see student examples while 80% of the respondents (n=8) found their knowledge of CVs increased because of the session. On a Likert scale from 1–5, with a 1 indicating “very unconfident” and 5 indicating “very confident,” 90% of respondents (n=9) indicated they are now confident or very confident in building or updating their CV after this session.

**Discussion:**

Overall, the educational content was found to be effective. Although the sample size from the survey was modest at best, we feel the survey data and comments from attendees during and after the session indicate the effectiveness of the content. From its initial implementation, we learned that this lecture can be given by any level of medical education professional (student, administrator, etc) due to the comprehensiveness of the presentation. We also learned that using video conferencing such as Zoom was an effective administration method but could also be replaced by in-person learning without much difficulty. Overall, we deem this presentation to be easy to administer, thorough, full of examples, and educationally effective.

**Topics:**

Curriculum vitae, CV, medical student, residency application, electronic residency application service, ERAS.

## USER GUIDE

[Table t1-jetem-9-1-l1]

**Table t1-jetem-9-1-l1:** 

List of Resources:	
Abstract	1
User Guide	3
Appendix 1: CV Lecture PowerPoint	5
Appendix 2: CV Sample 1	6
Appendix 3: CV Sample 2	8
Appendix 4: CV Sample 3	10
Appendix 5: CV Sample 4	11
Appendix 6: CV Sample 5	12
Appendix 7: CV Sample 6	14
Appendix 8: CV Sample 7	15
Appendix 9: CV Sample 8	17

**Learner Audience:**

Medical Students, Interns, Junior Residents, Senior Residents, Attending Physicians, Anyone that needs an academic medical CV

**Time Required for Implementation:** 40–45 minutes

**Recommended Number of Learners per Instructor:** 1–125

**Topics:**

Curriculum vitae, CV, medical student, residency application, electronic residency application service, ERAS.

**Objectives:**

After this lecture, learners should be able to:

1. Elaborate on the significance of a CV for medical students and discuss its purpose
2. Outline the elements that should and should not be included on a CV
3. Integrate knowledge gleaned from basic principles with provided examples to establish the foundation of their own CV

### Linked objectives and methods

Objectives 1 and 2 are accomplished by the instructor’s presentation and discussion of the 15-slide PowerPoint. However, objective 3 is accomplished by including a space for students to discuss and present ideas after reviewing each example CV. This format was selected because the basic principles and definitions are quick, but much of the benefit of the presentation is based upon utilizing the examples as frameworks, each with their own unique features. This format was also selected to encourage discussion between learners instead of the presenter being the only one contributing to the discussion. At the completion of the session, learners can begin working on their CV and submit it to the presenter or other learners to provide feedback.

### Recommended pre-reading for instructor

The instructor should review the content within the presentation as well as the resources that were used to create the presentation and are provided to the students at the end of the presentation.

- Resume vs. curriculum vitae: What’s the difference? In: Internship and Career Center. Published May 29, 2020. Accessed: December 24, 2022. https://icc.ucdavis.edu/materials/resume/resumecv
- Curriculum vitae – tips and strategies. In: AAMC. Accessed December 24, 2022. https://www.aamc.org/career-development/affinity-groups/group-faculty-affairs/faculty-vitae/curriculum-vitae-tips-and-strategies
- Preparing your curriculum vitae. In: AAMC. Accessed: December 26, 2022. https://students-residents.aamc.org/managing-your-medical-career/preparing-your-curriculum-vitae

### Resources for Learners

- EMRA and CORD Student Advising Guide. Ch. 7 – Building your ERAS application. In: EMRA. Accessed: January 1, 2023. https://www.emra.org/books/msadvisingguide/building-your-eras-application
- What’s new in the 2024 MyERAS application. In: AAMC. Accessed: May 1 2023. https://students-residents.aamc.org/applying-residencies-eras/what-s-new-2024-myeras-application
- The CV. Accessed May 1, 2023. https://medicine.osu.edu/student-resources/career-advising/the-cv
- Medicine CVs, resumes, and cover letters. Accessed May 1, 2023. https://career.ucsf.edu/professional/medicine/cvs-resumes-cover-letters#Residency-Focused-CV-Samples

### Results and Tips for Successful Implementation

This CV writing session was presented to 36 students at the University of California, Irvine, School of Medicine in a peer-to-peer style lecture. The session ran 72 minutes. After completion of the session, learners were provided with a short survey to assess their satisfaction with the session and recommendations for future sessions. We received 10 responses with 10 (100%) stating that they found the session to be useful and 10 (100%) reporting that they appreciate the ability to see CV examples. Eight (80%) reported that they felt their knowledge about CVs improved at completion of the session, and nine (90%) felt confident in building their own CV after the session. Written comments included: “This was a great session, which helped me update my resume as medical student. It answered many questions I had about what to include and what is unnecessary to include.” “I adjusted the format of my CV based on the advice, specifically creating more sections for educational workshops and community events.” “Some important things I learned from this session were to not include any grades/test scores now that we are at the med school level. Also, I put my achievements and awards higher up!”

This lecture can be implemented in many ways: as a presentation during medical school orientation for first year students, in small group by career advisors, specialty specific advisors and mentors, or it could be given by upper-level students to the class years below them. It may be given via video conferencing or in-person. Also, we recommend representing the lecture to third year students as they begin to finalize their CV for their residency applications. Although this presentation was created for medical students, it can also be adapted for resident learners, fellows, and junior faculty.

Modifications were made from the initial presentation to bring it to its current form. Presenter notes were added, principles were expanded upon and made more concise, and examples were worked upon to present a broader set of principles and ensure that their material aligns with CV best practices for medical students.

### Associated content (optional)

Example and template CVs are included.

### Technology necessary

Ability to share the presentation while giving the lecture is necessary; thus a computer with overhead projector or screen sharing on a large monitor/TV should be utilized. There are no imbedded audio or video files.

### Assessment (optional)

No formal assessment is necessary after the lecture; however, we recommend having students either turn in their completed CV to faculty for written feedback or exchange their CVs with peers at the end of the session for peer-to-peer feedback.

## USER GUIDE AND LEARNER MATERIALS

### Appendix 1 CV Lecture PowerPoint

Please see associated PowerPoint file
