# Supplementary material for: A Guide to the Medical School Curriculum Vitae
Source: J Educ Teach Emerg Med. 2024 Jan 31;9(1):L1–L20. doi: 10.21980/J8HH1S (PMC10854880; doi:10.21980/J8HH1S)
Supplement: Supplementary file 1 — Please see associated PowerPoint file [file jetem-9-1-L1-supp1.pptx]

## Slide 1
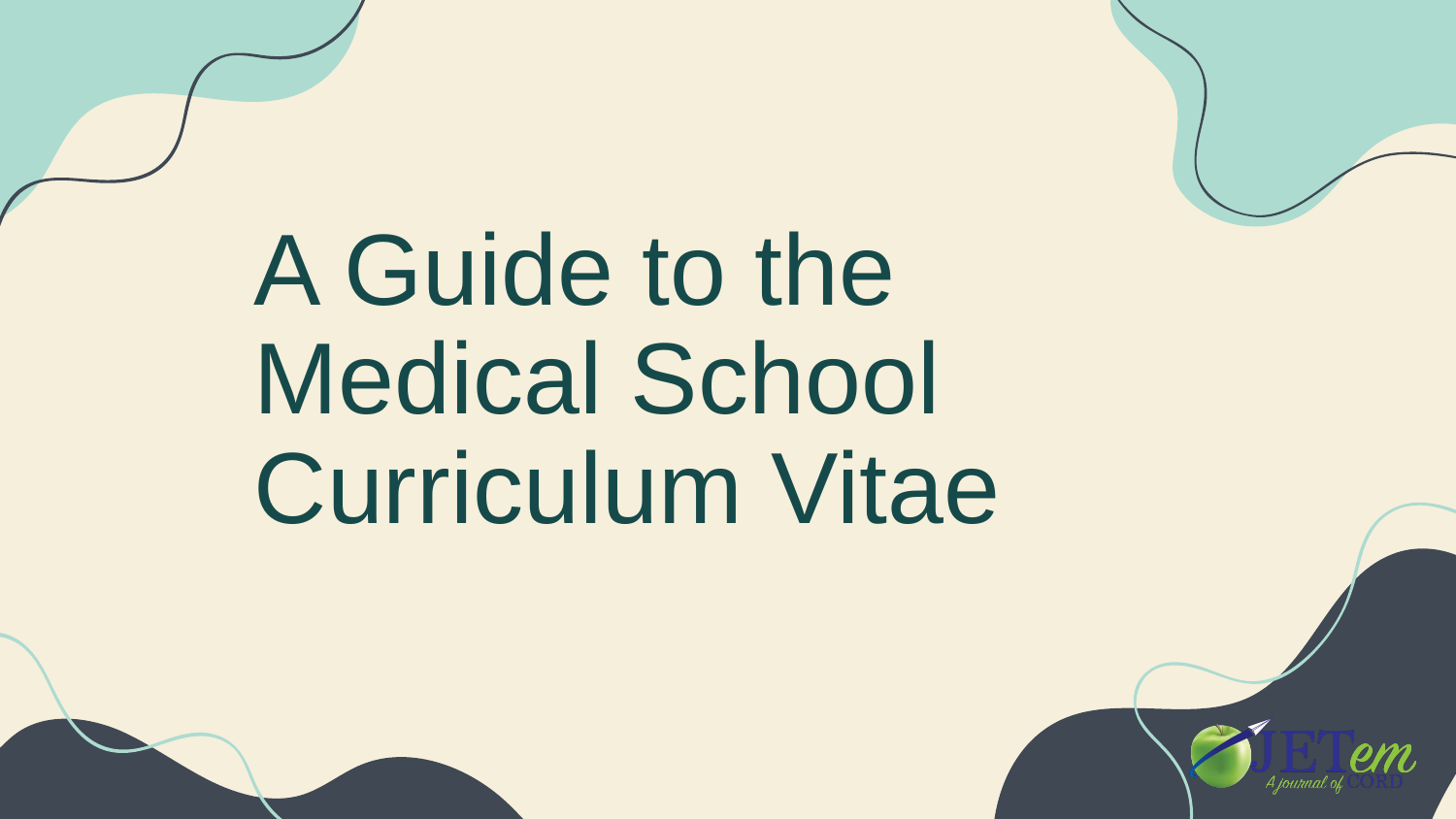

# A Guide to the Medical School Curriculum Vitae

## Slide 2
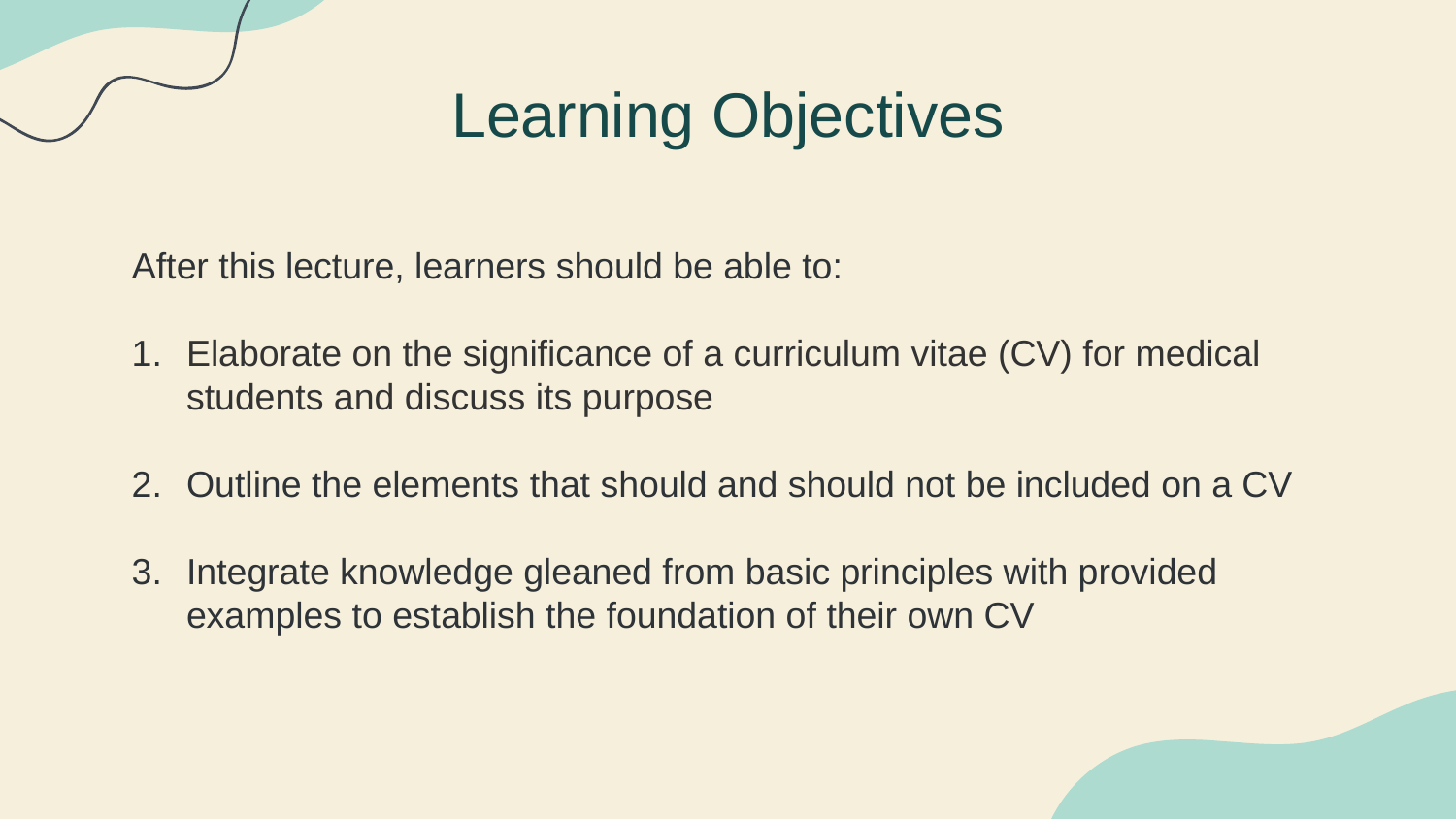

# Learning Objectives
After this lecture, learners should be able to:
Elaborate on the significance of a curriculum vitae (CV) for medical students and discuss its purpose
Outline the elements that should and should not be included on a CV
Integrate knowledge gleaned from basic principles with provided examples to establish the foundation of their own CV

## Slide 3
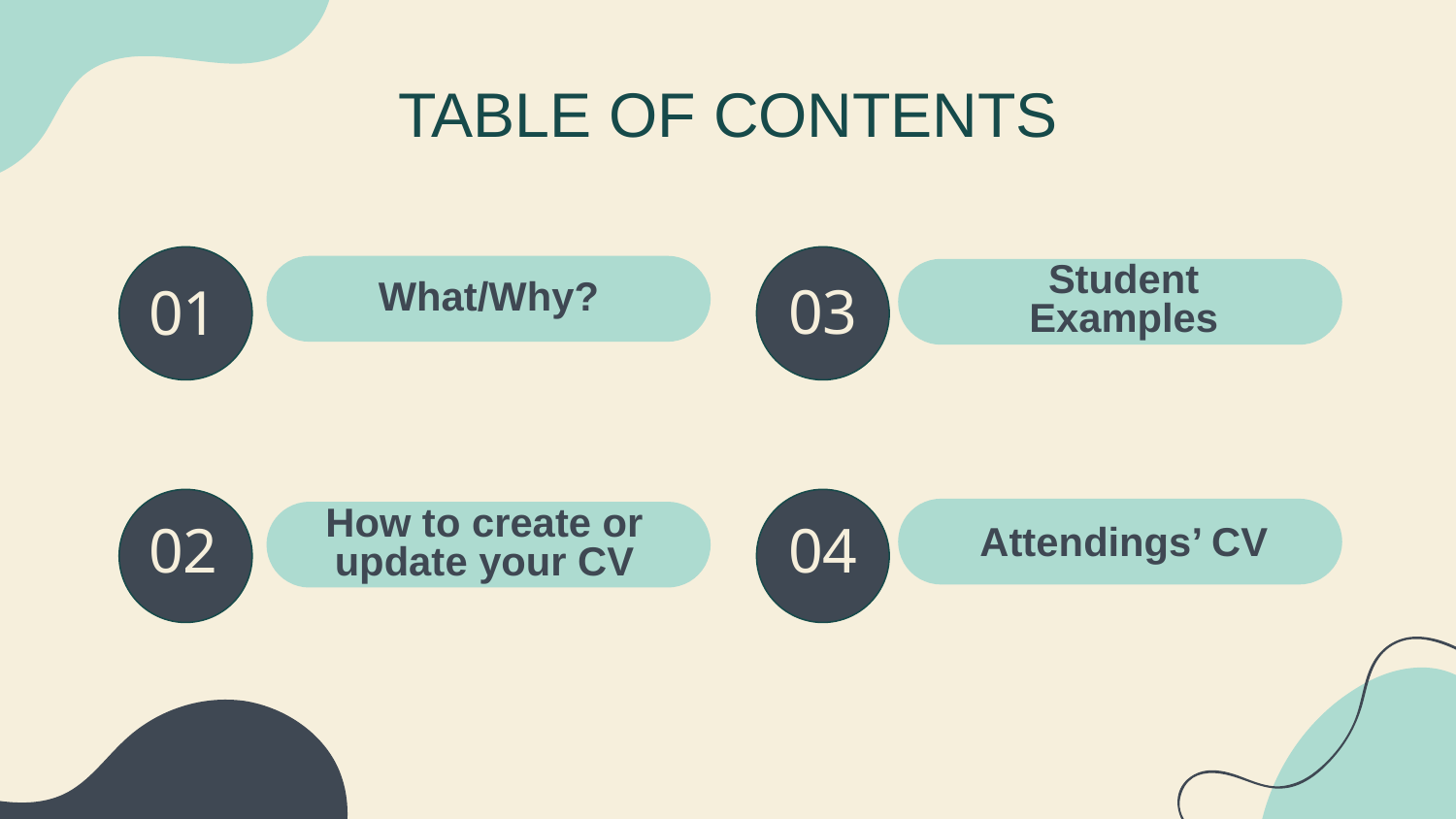

# TABLE OF CONTENTS
03
01
What/Why?
Student Examples
02
04
How to create or update your CV
Attendings’ CV

## Slide 4
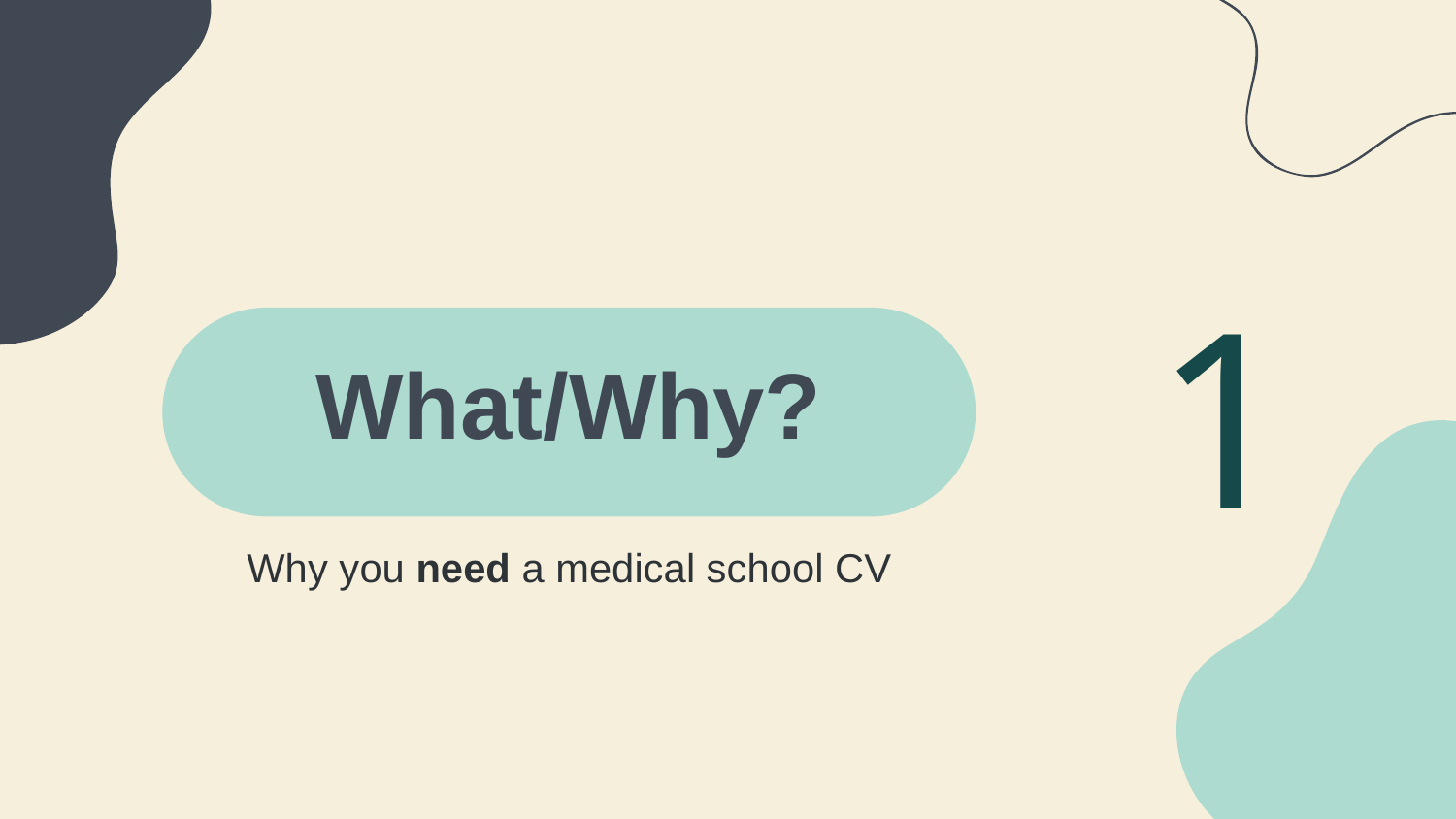

1
# What/Why?
Why you need a medical school CV

## Slide 5
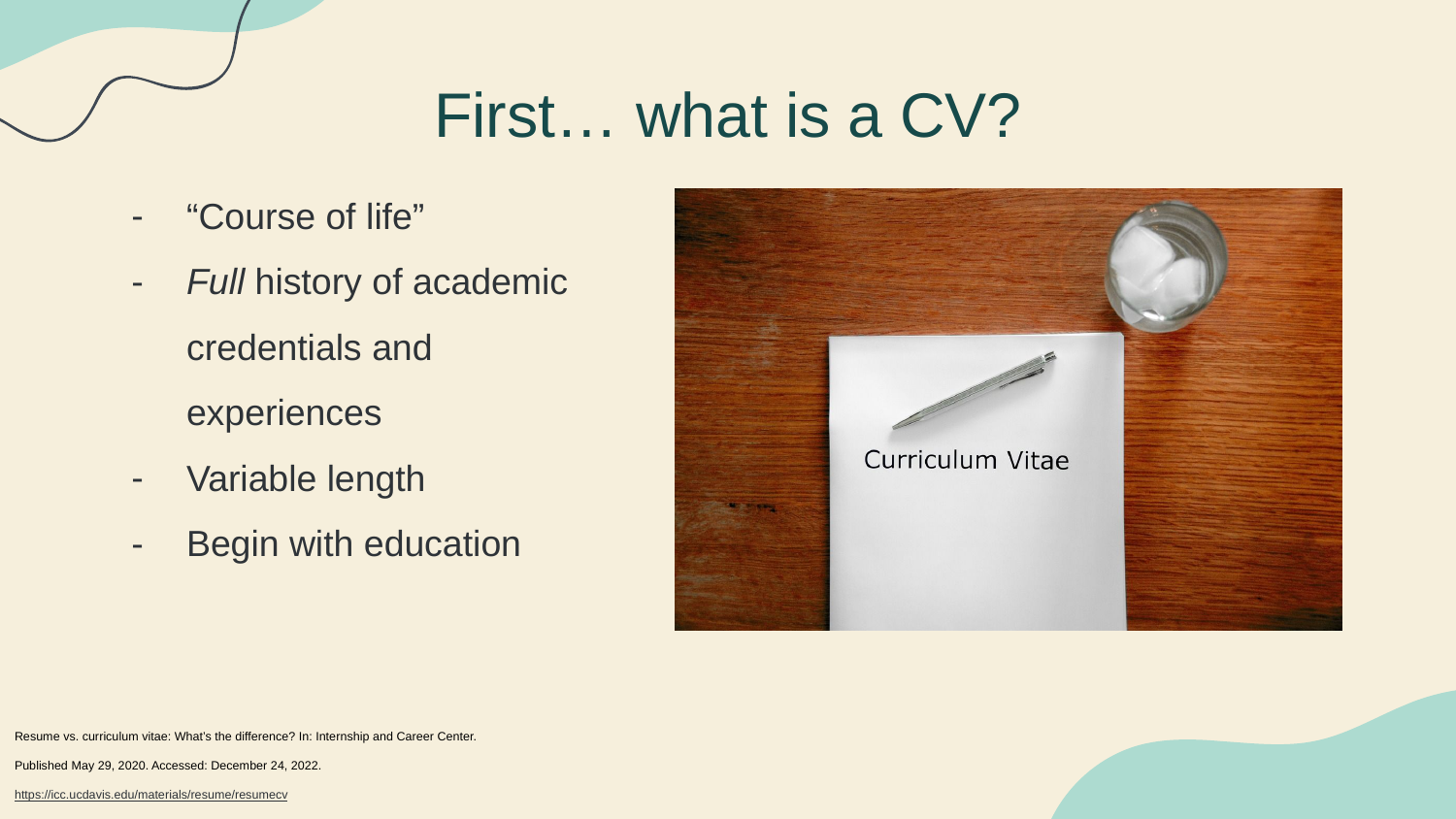

# First… what is a CV?
“Course of life”
Full history of academic credentials and experiences
Variable length
Begin with education
Resume vs. curriculum vitae: What’s the difference? In: Internship and Career Center. Published May 29, 2020. Accessed: December 24, 2022. https://icc.ucdavis.edu/materials/resume/resumecv

## Slide 6
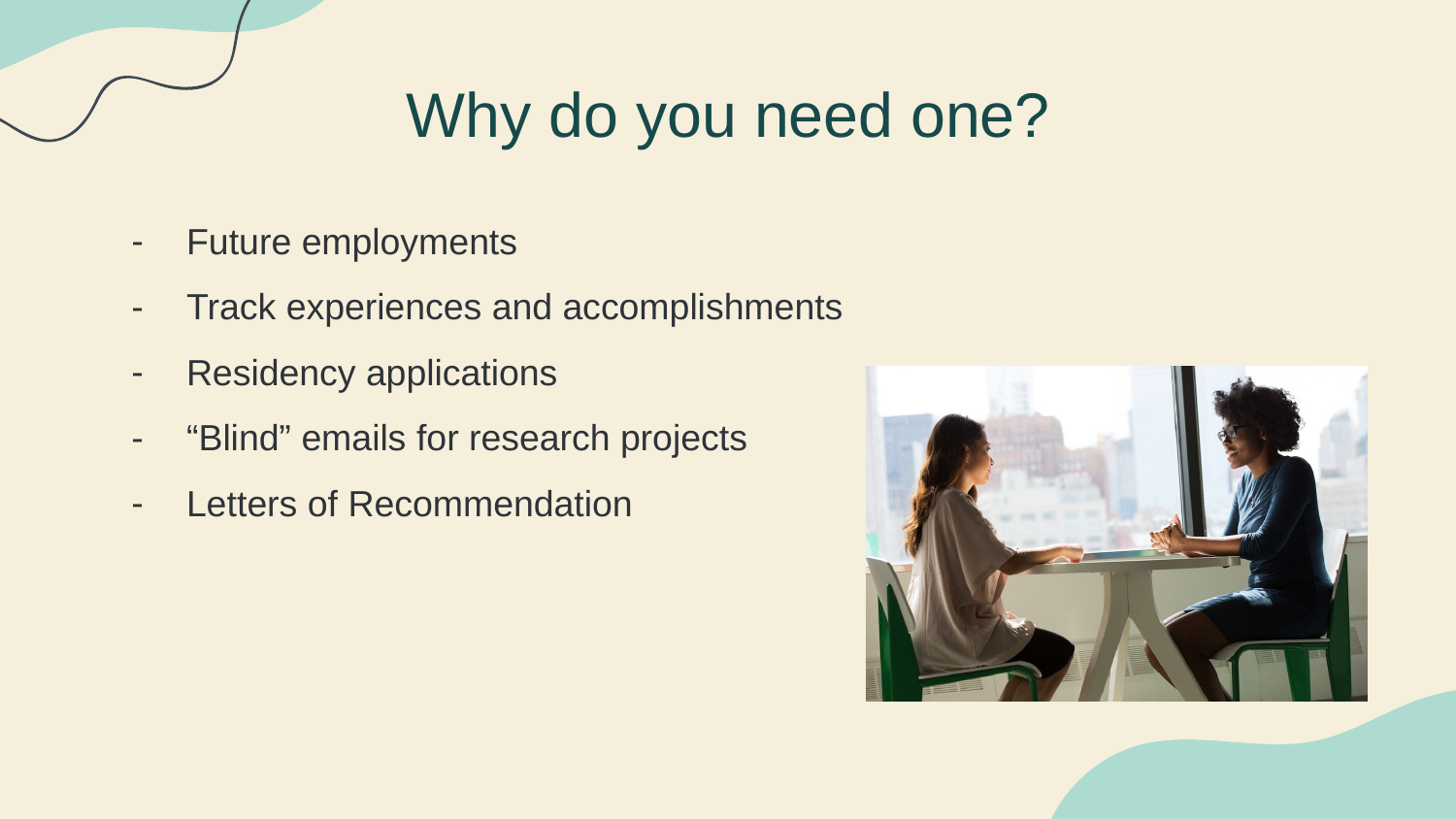

# Why do you need one?
Future employments
Track experiences and accomplishments
Residency applications
“Blind” emails for research projects
Letters of Recommendation

## Slide 7
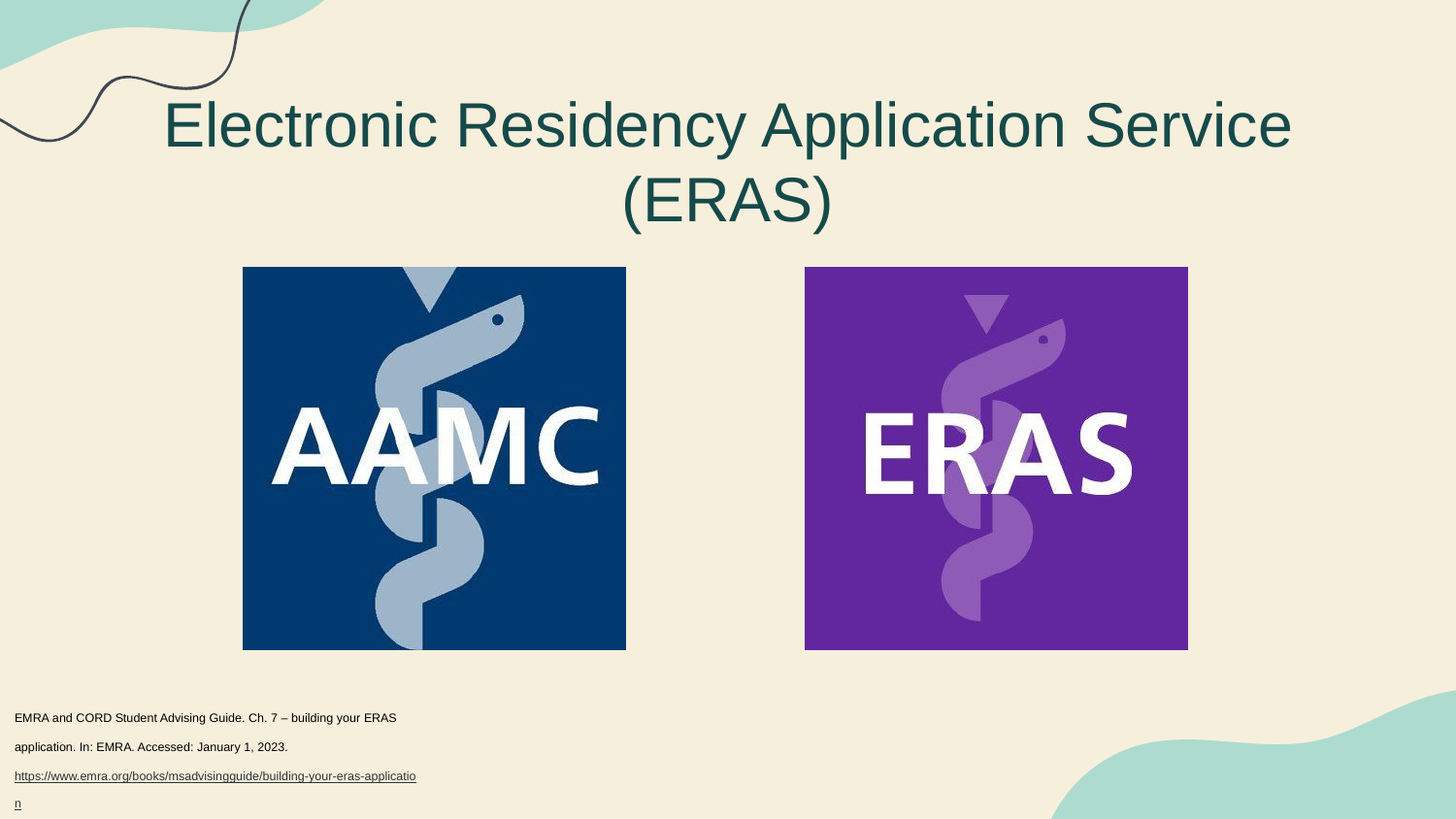

# Electronic Residency Application Service (ERAS)
EMRA and CORD Student Advising Guide. Ch. 7 – building your ERAS application. In: EMRA. Accessed: January 1, 2023. https://www.emra.org/books/msadvisingguide/building-your-eras-application

## Slide 8
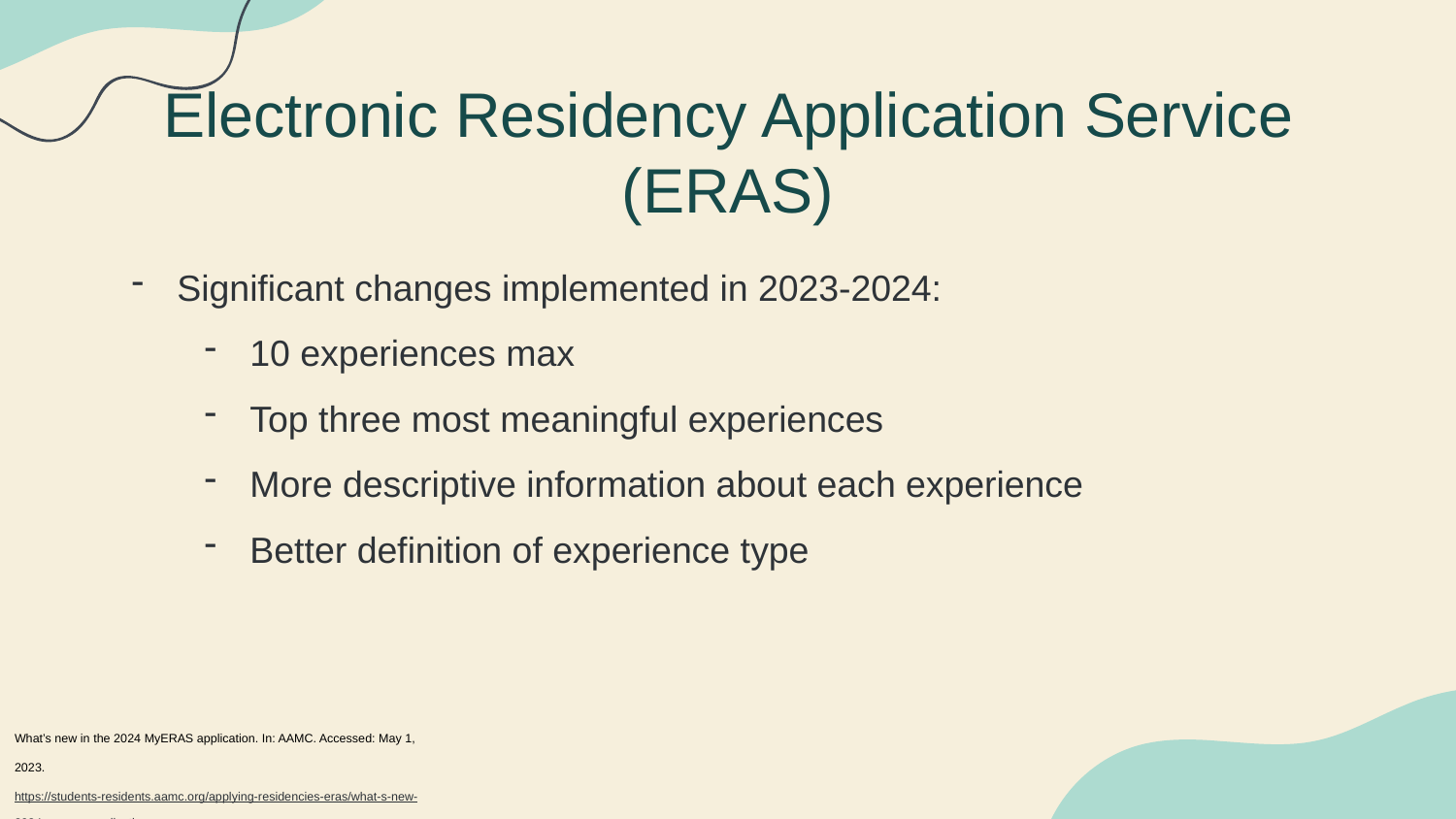

# Electronic Residency Application Service (ERAS)
Significant changes implemented in 2023-2024:
10 experiences max
Top three most meaningful experiences
More descriptive information about each experience
Better definition of experience type
What’s new in the 2024 MyERAS application. In: AAMC. Accessed: May 1, 2023. https://students-residents.aamc.org/applying-residencies-eras/what-s-new-2024-myeras-application

## Slide 9
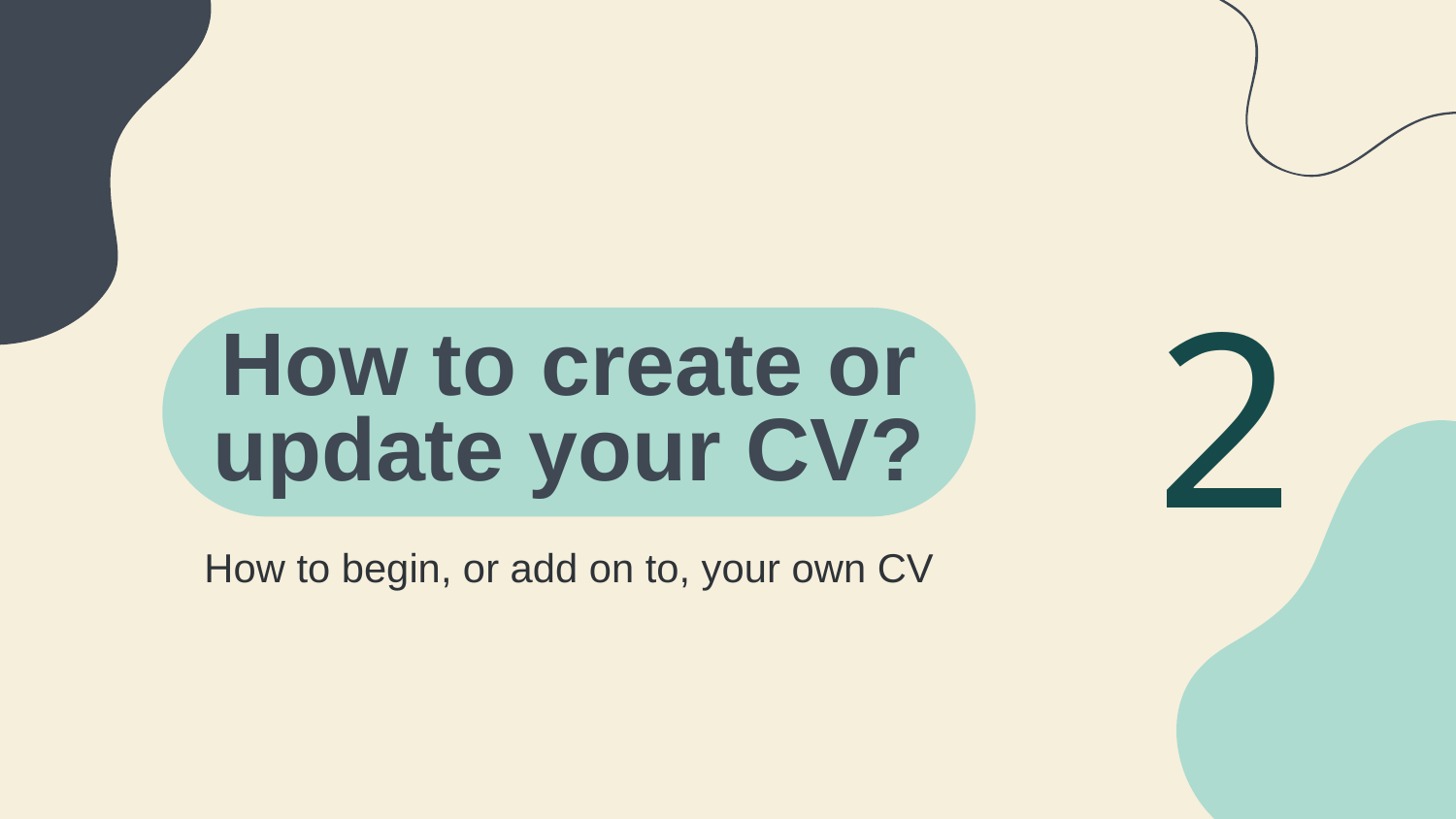

2
# How to create or update your CV?
How to begin, or add on to, your own CV

## Slide 10
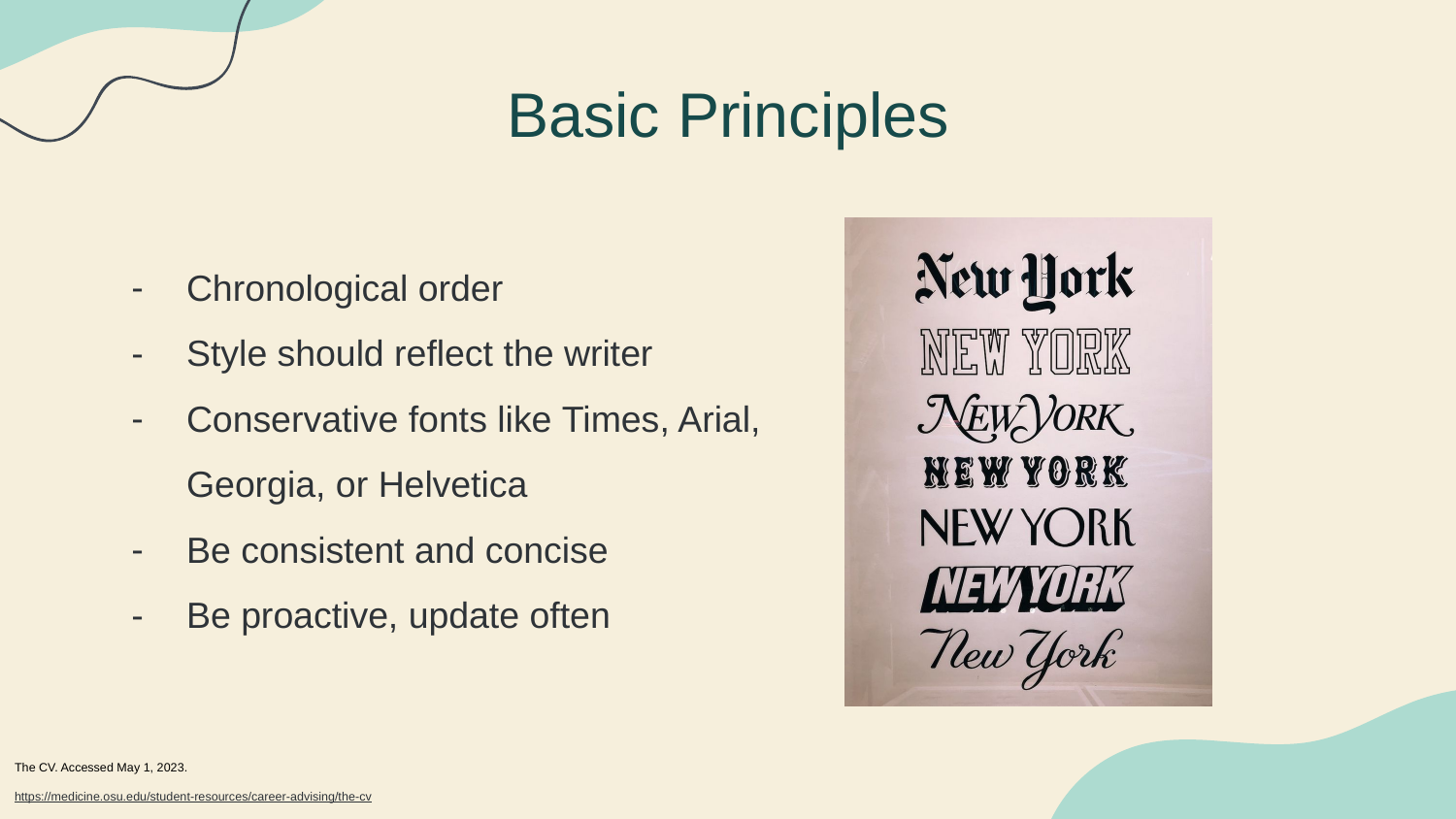

# Basic Principles
Chronological order
Style should reflect the writer
Conservative fonts like Times, Arial, Georgia, or Helvetica
Be consistent and concise
Be proactive, update often
The CV. Accessed May 1, 2023. https://medicine.osu.edu/student-resources/career-advising/the-cv

## Slide 11
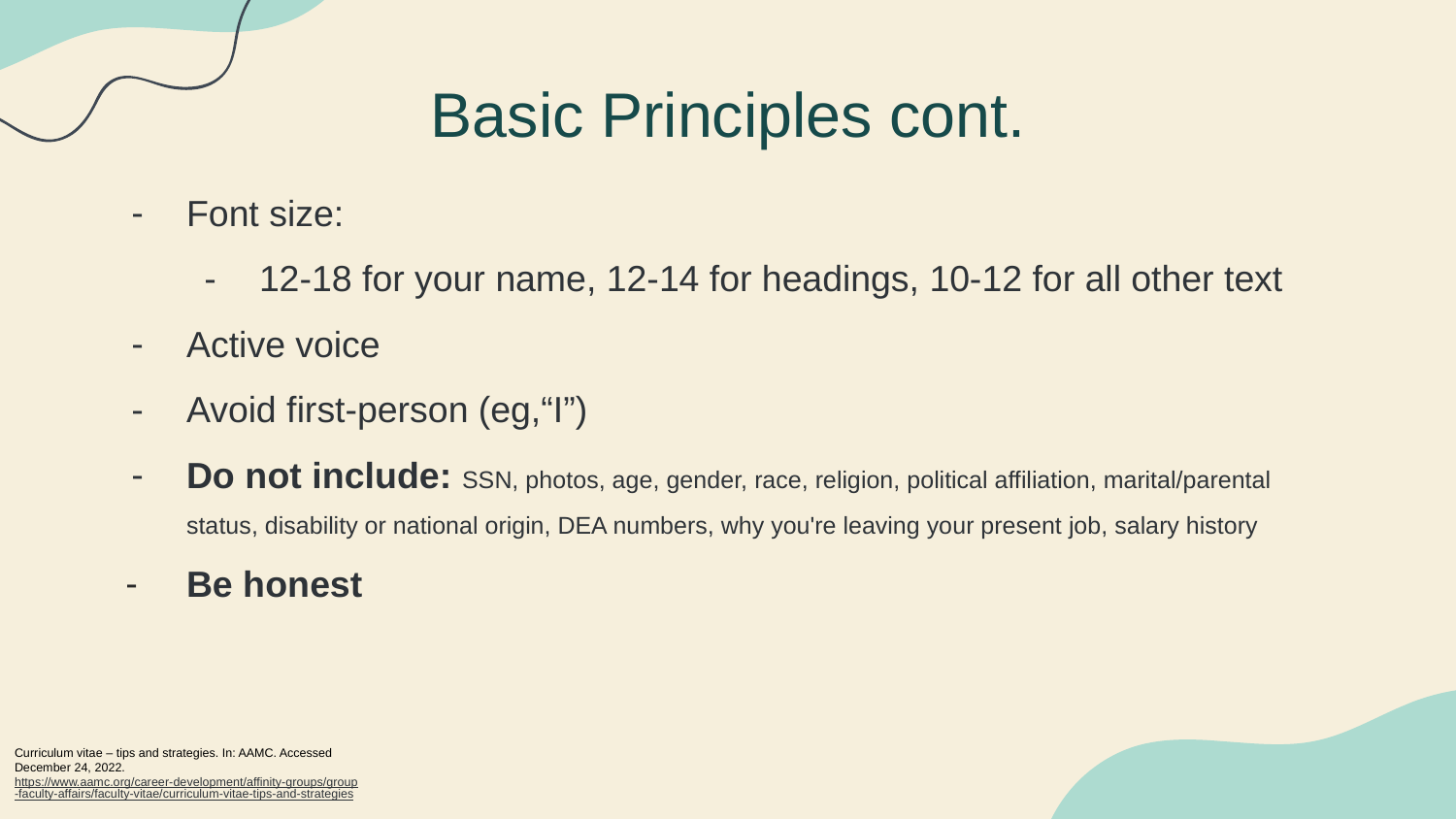

# Basic Principles cont.
Font size:
12-18 for your name, 12-14 for headings, 10-12 for all other text
Active voice
Avoid first-person (eg,“I”)
Do not include: SSN, photos, age, gender, race, religion, political affiliation, marital/parental status, disability or national origin, DEA numbers, why you're leaving your present job, salary history
Be honest
Curriculum vitae – tips and strategies. In: AAMC. Accessed December 24, 2022. https://www.aamc.org/career-development/affinity-groups/group-faculty-affairs/faculty-vitae/curriculum-vitae-tips-and-strategies

## Slide 12
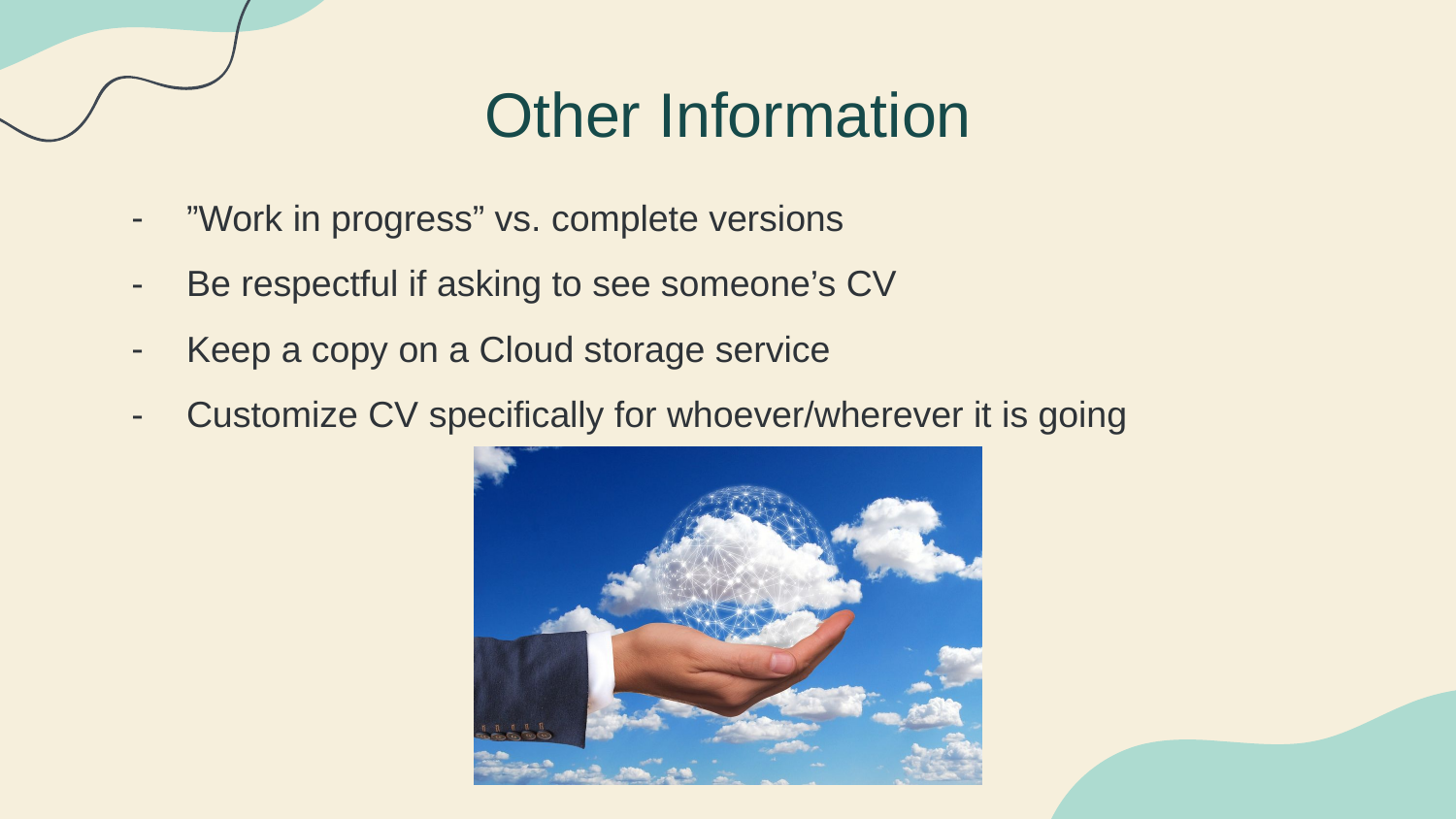

# Other Information
”Work in progress” vs. complete versions
Be respectful if asking to see someone’s CV
Keep a copy on a Cloud storage service
Customize CV specifically for whoever/wherever it is going

## Slide 13
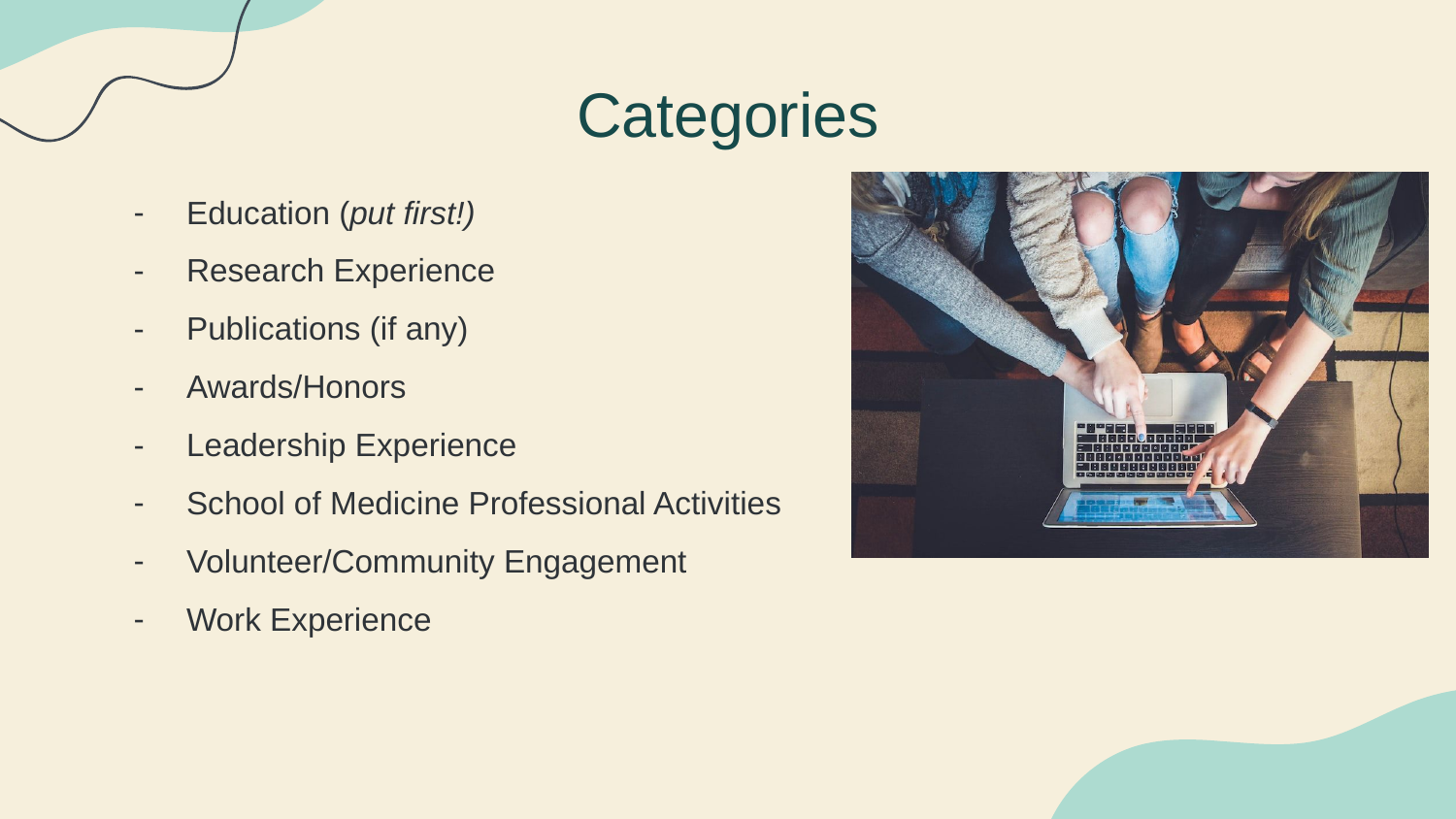

# Categories
Education (put first!)
Research Experience
Publications (if any)
Awards/Honors
Leadership Experience
School of Medicine Professional Activities
Volunteer/Community Engagement
Work Experience

## Slide 14
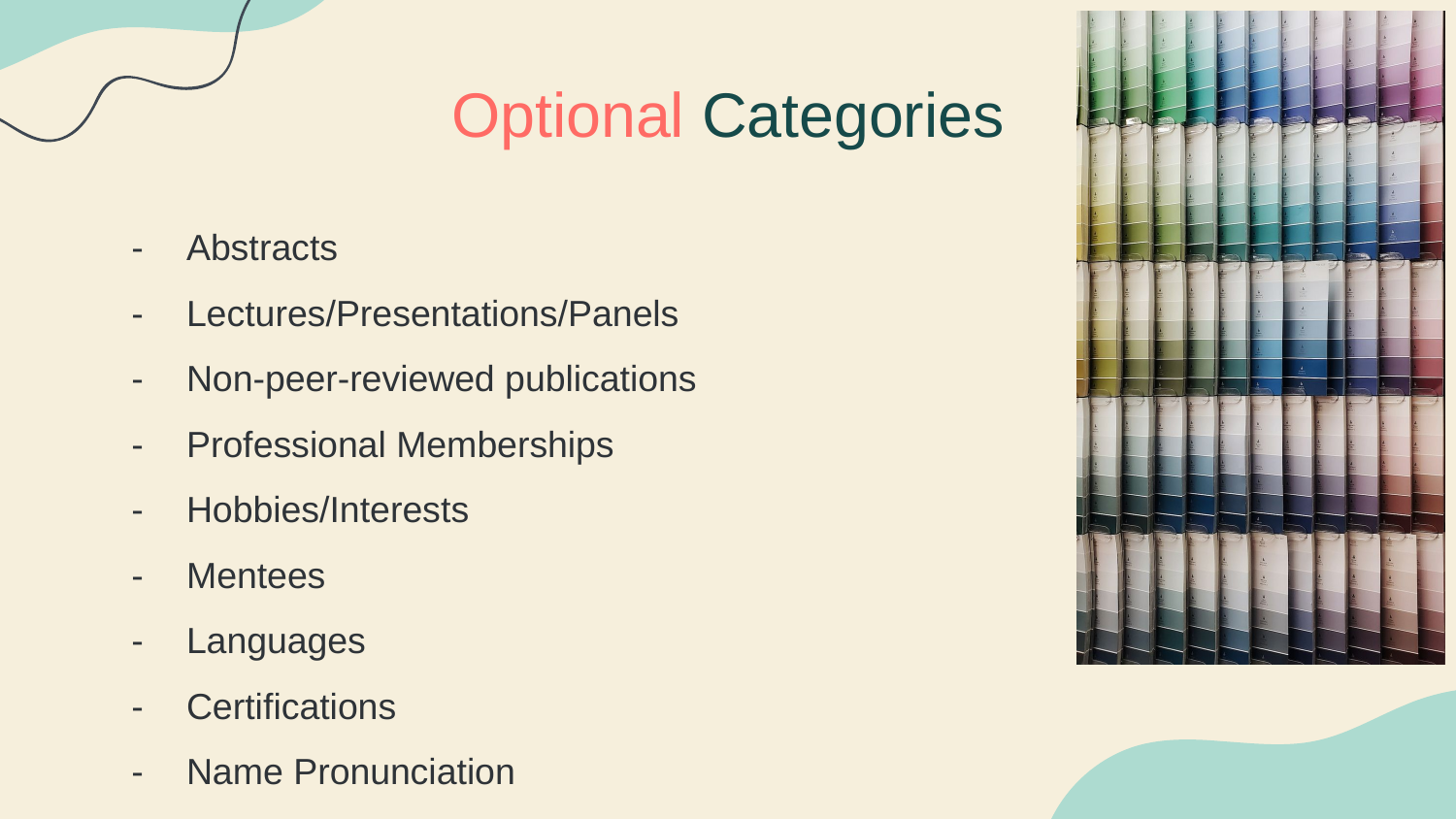

# Optional Categories
Abstracts
Lectures/Presentations/Panels
Non-peer-reviewed publications
Professional Memberships
Hobbies/Interests
Mentees
Languages
Certifications
Name Pronunciation

## Slide 15
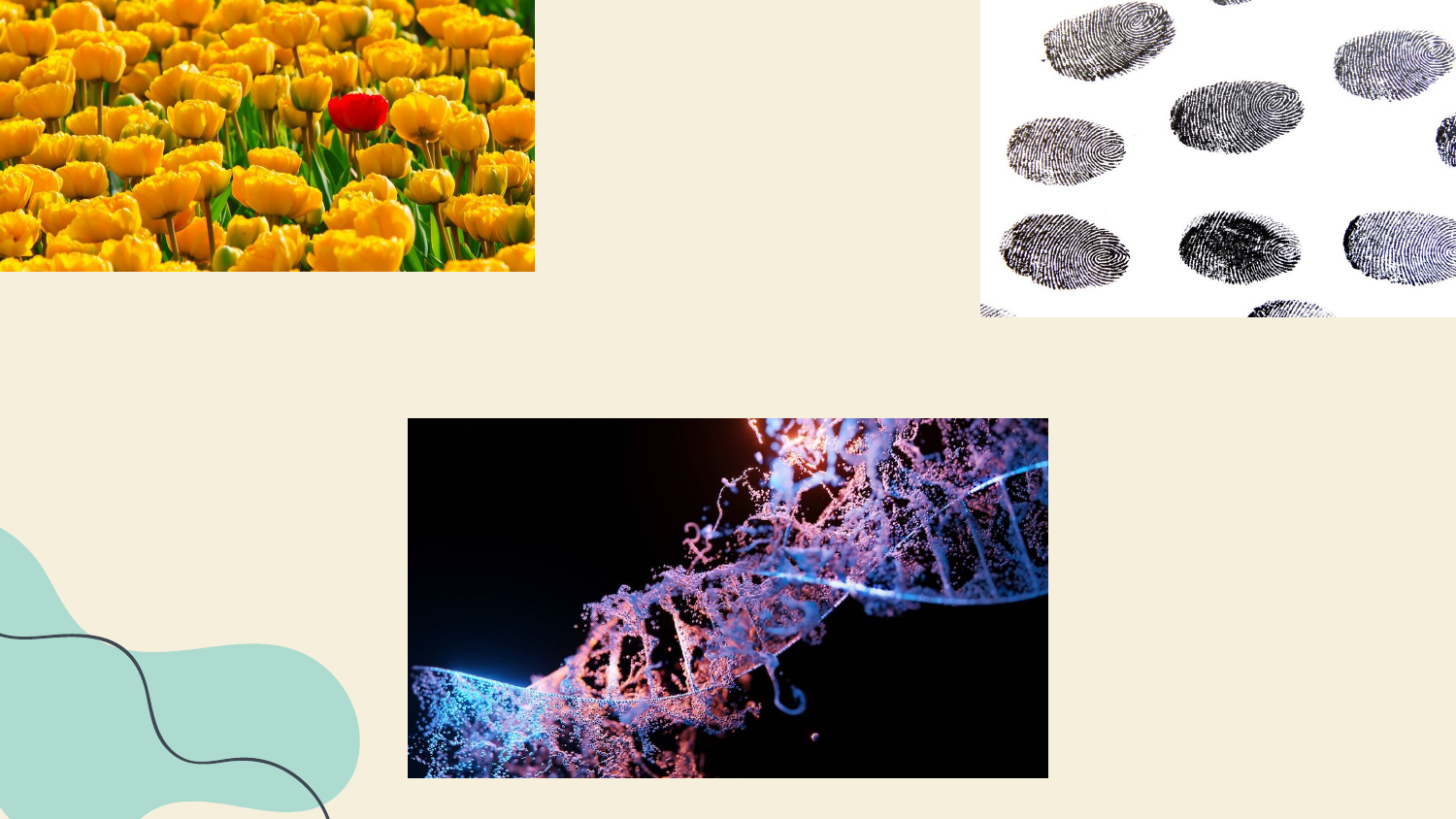

## Slide 16
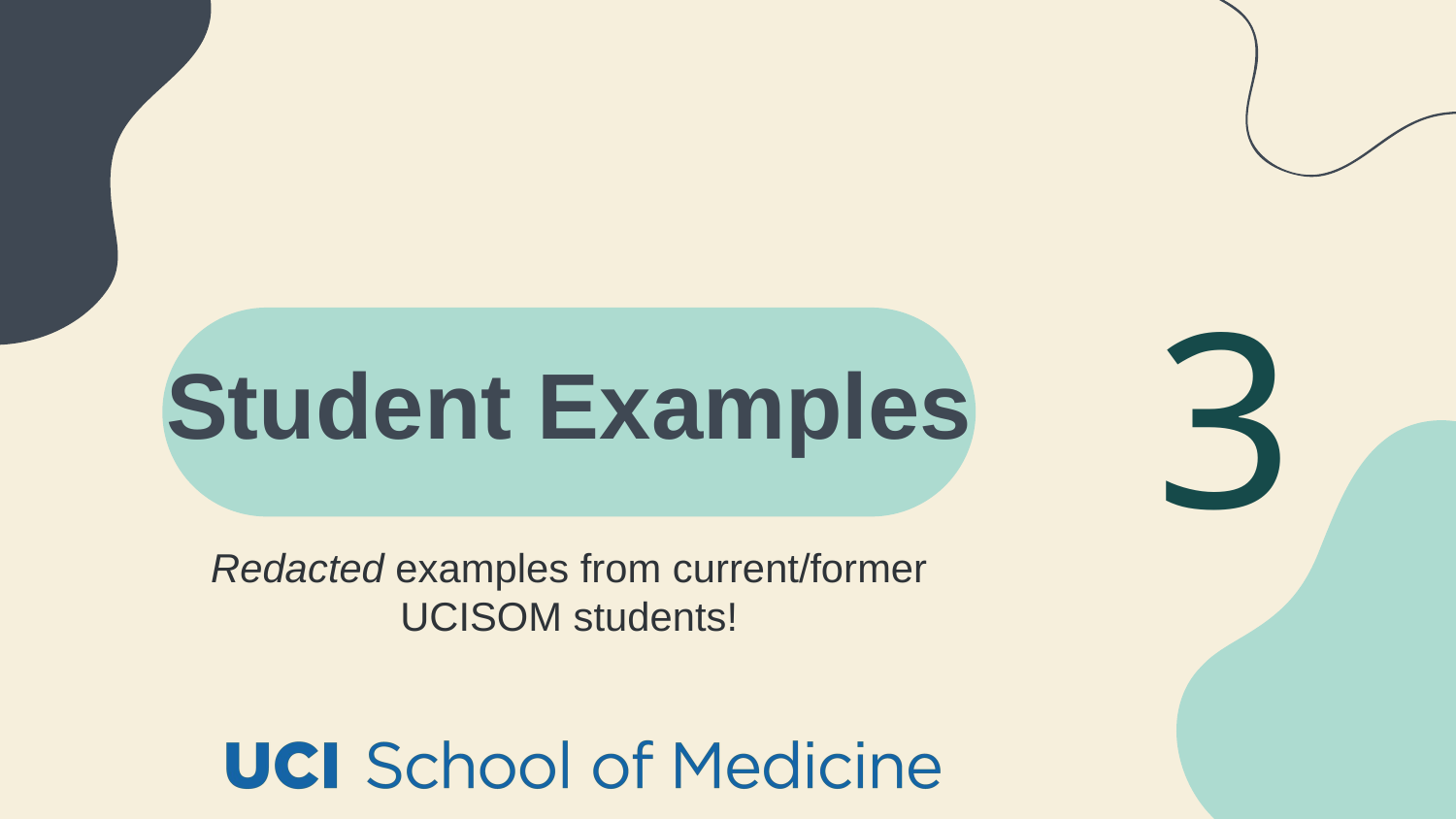

3
# Student Examples
Redacted examples from current/former UCISOM students!

## Slide 17
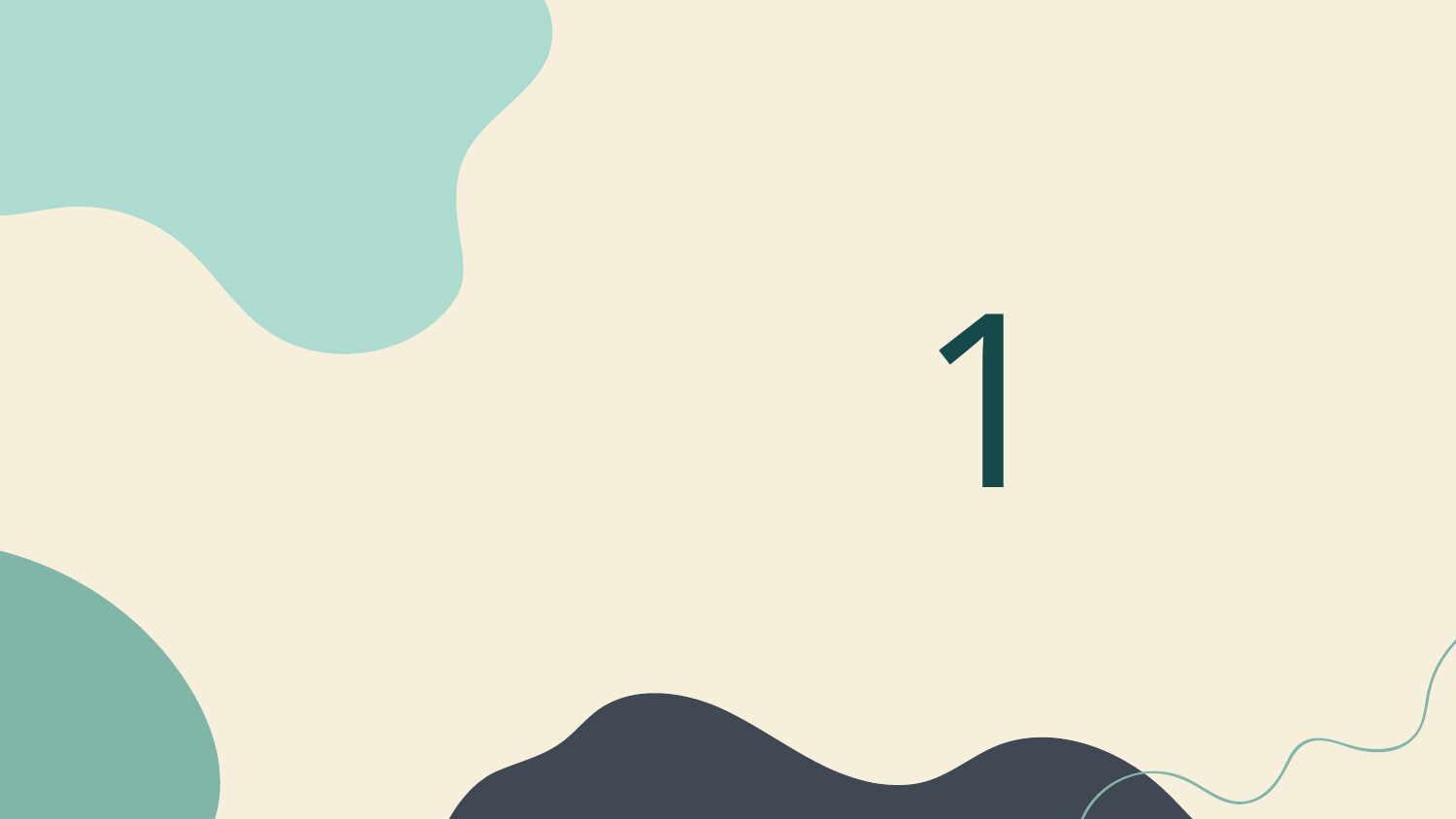

# 1

## Slide 18
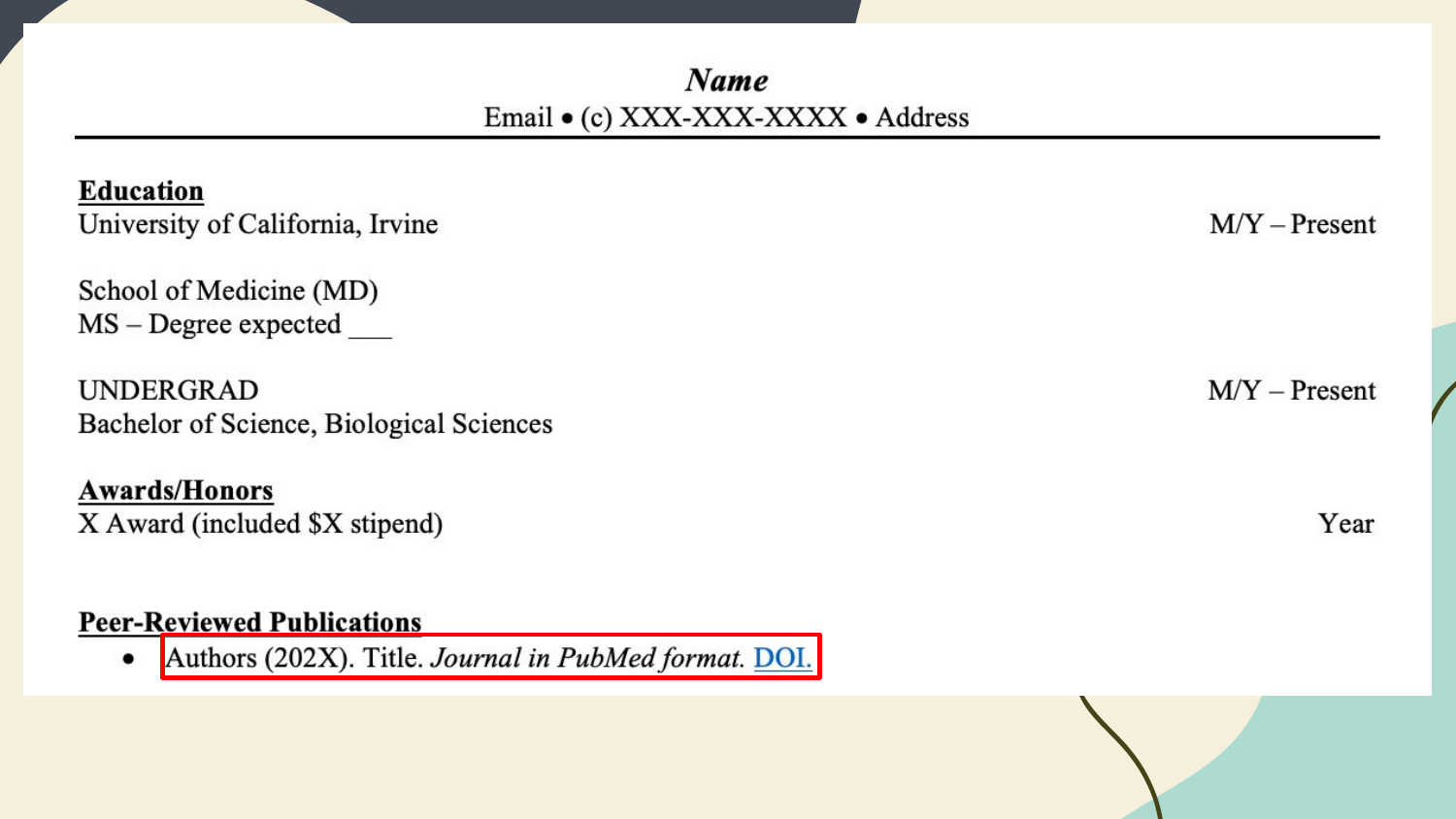

## Slide 19
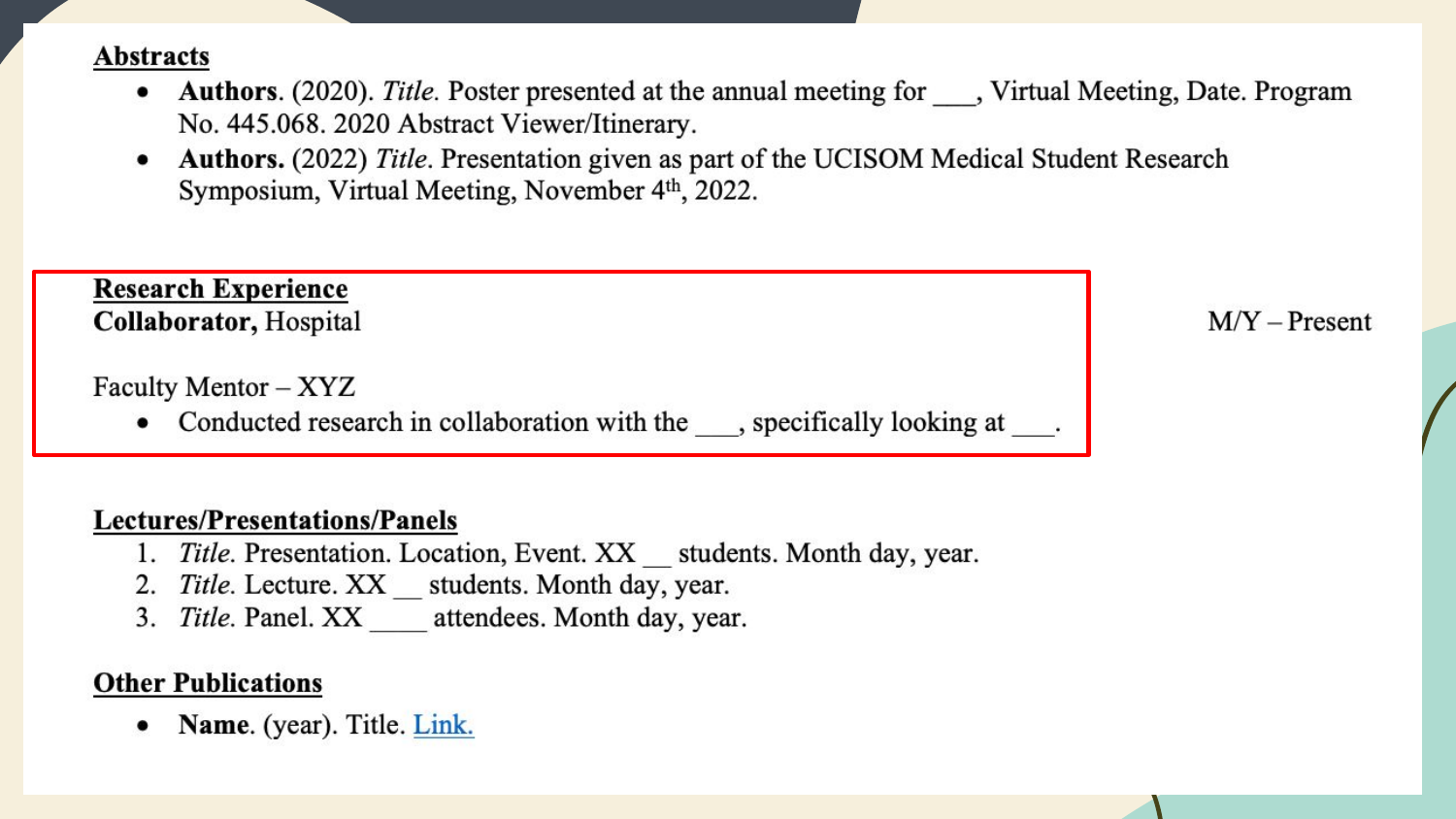

## Slide 20
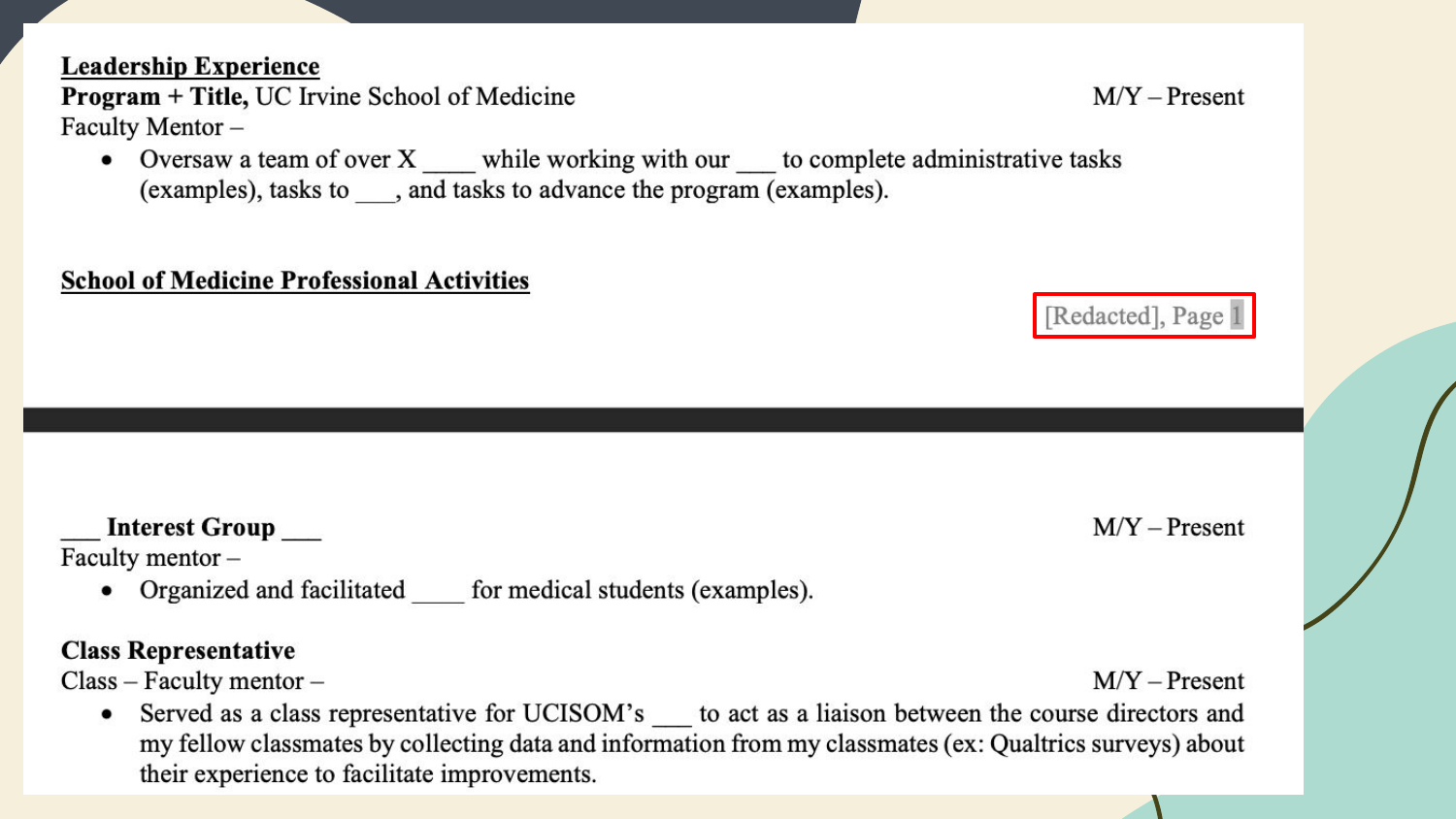

## Slide 21
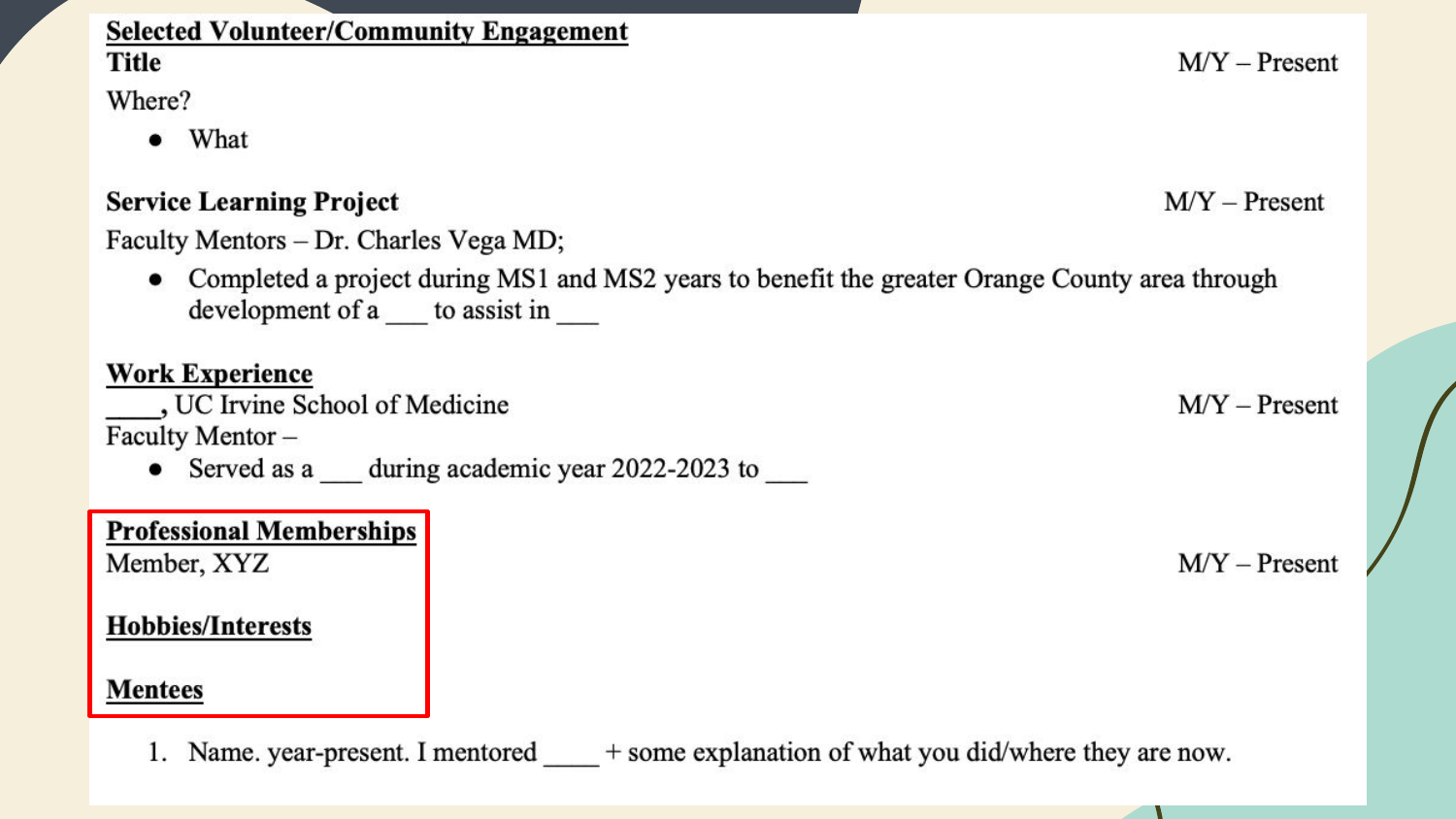

## Slide 22
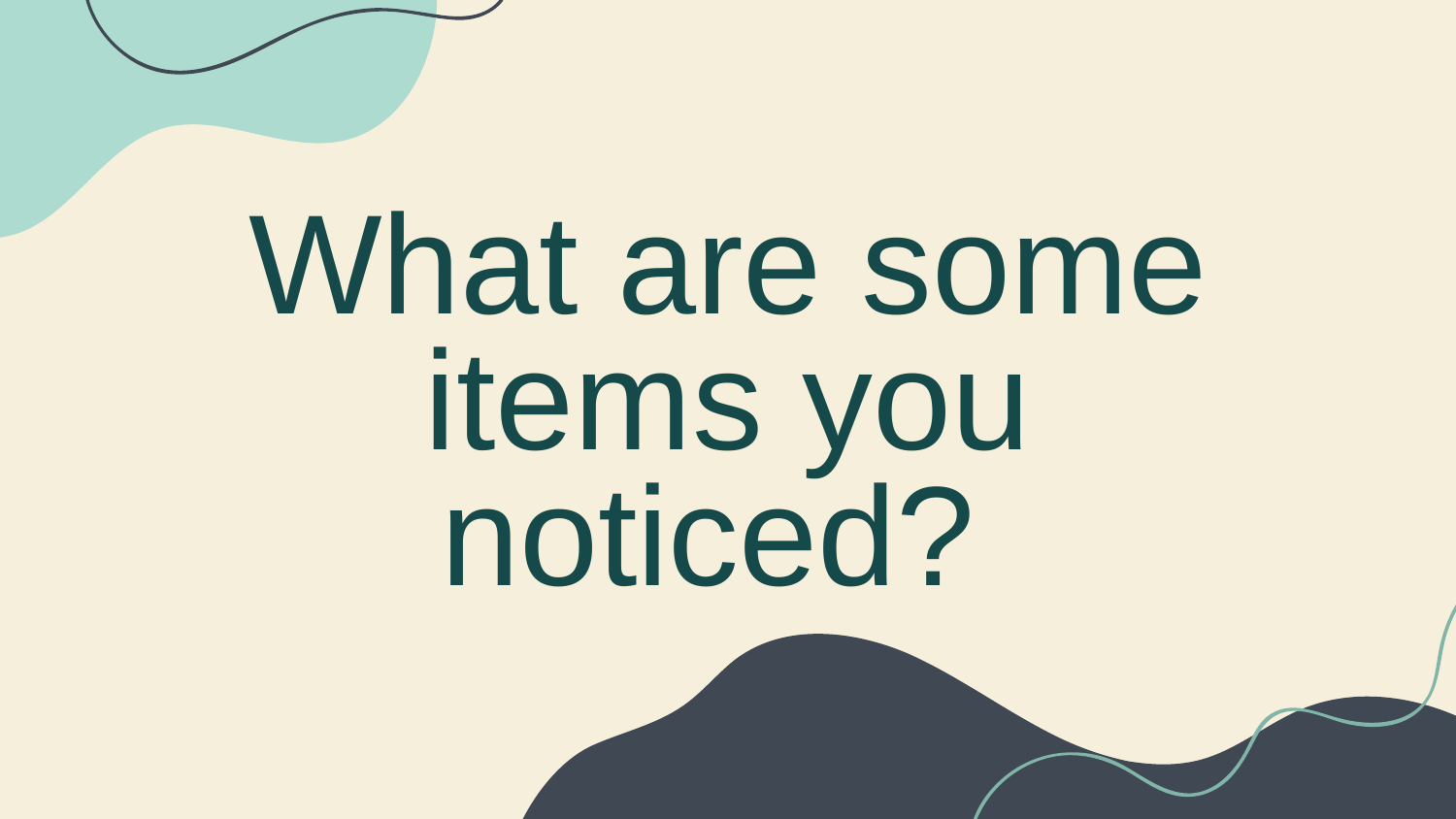

# What are some items you noticed?

## Slide 23
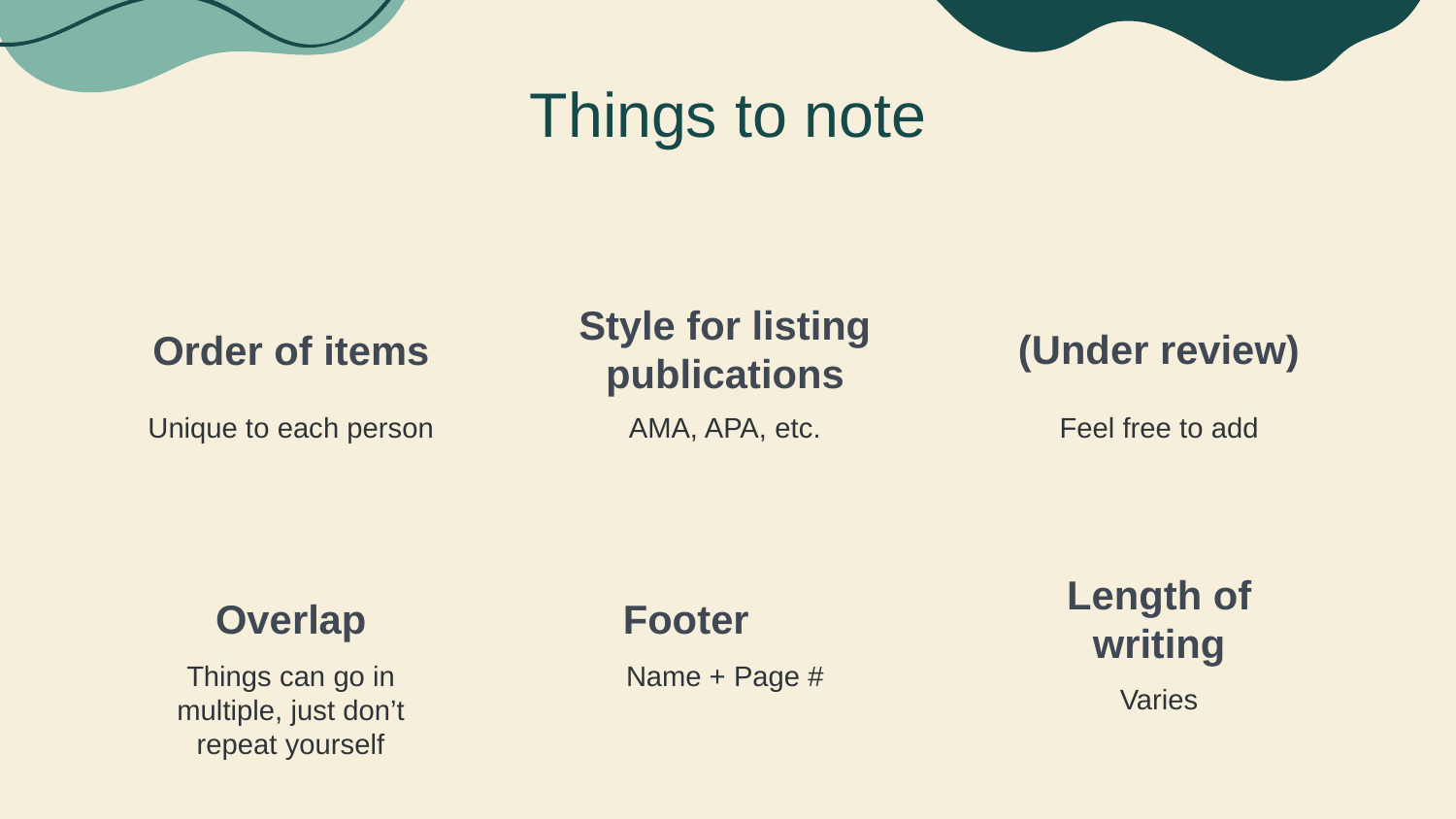

# Things to note
Style for listing publications
(Under review)
Order of items
AMA, APA, etc.
Feel free to add
Unique to each person
Overlap
Footer
Length of writing
Things can go in multiple, just don’t repeat yourself
Name + Page #
Varies

## Slide 24
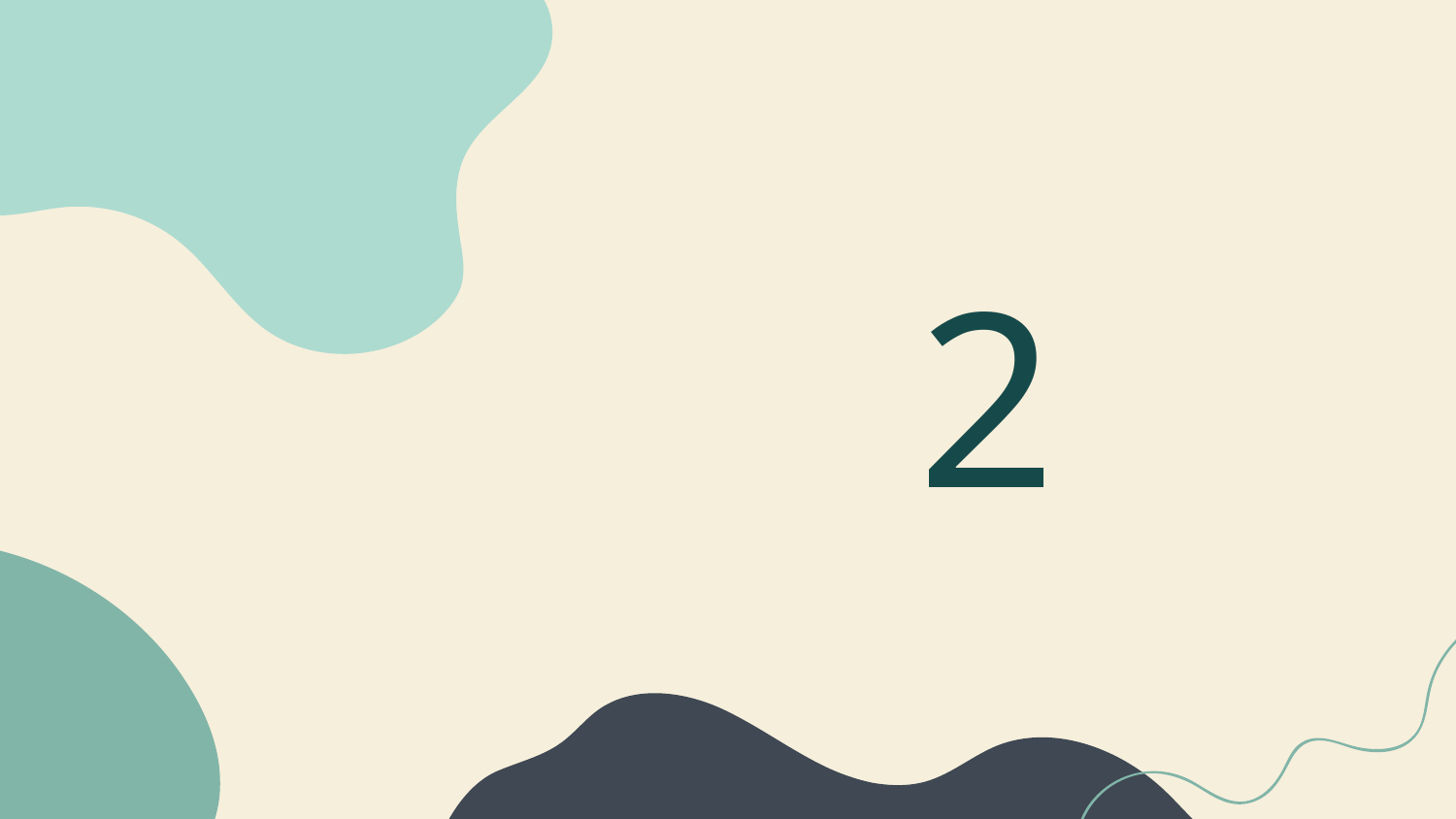

# 2

## Slide 25
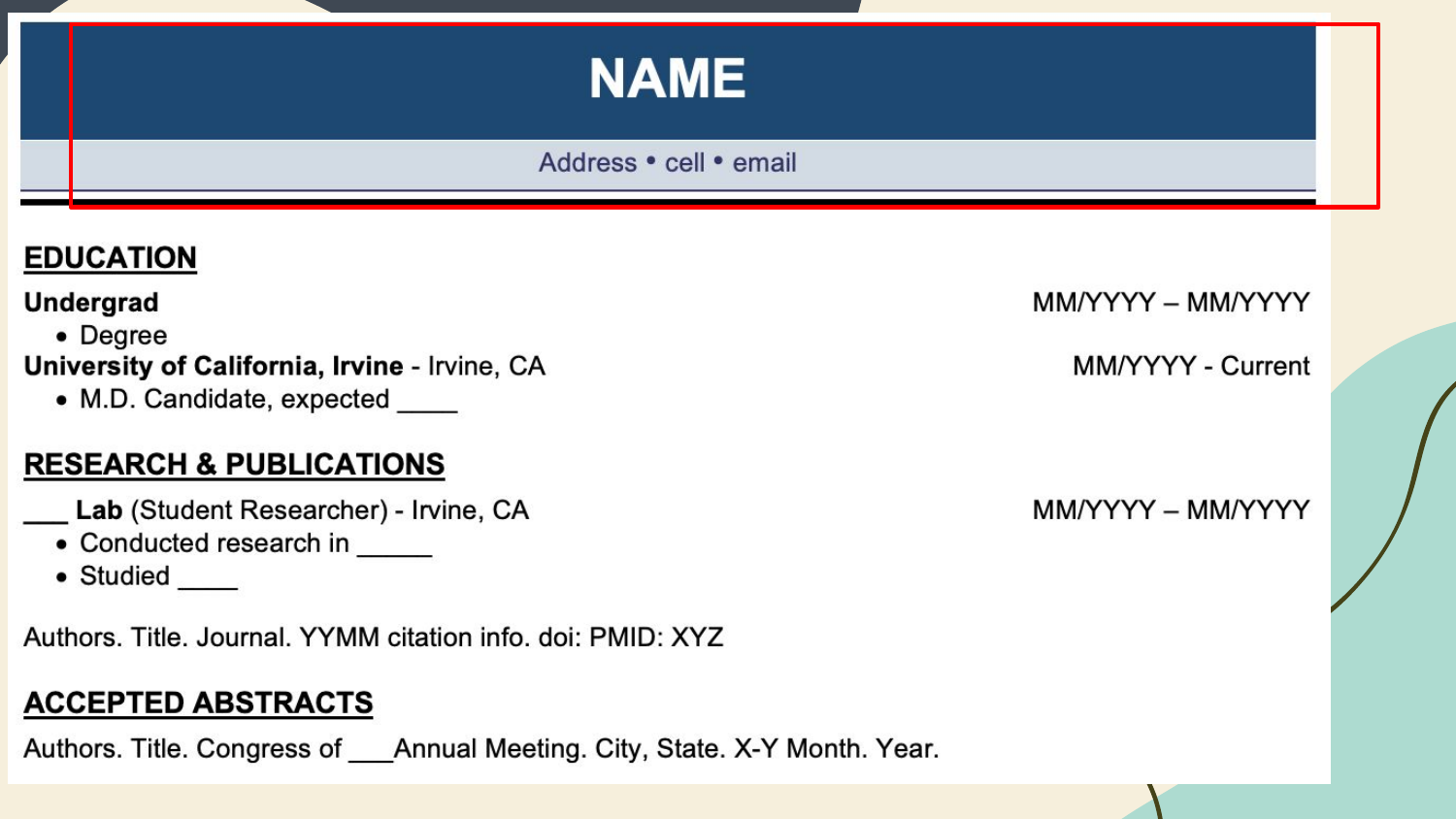

## Slide 26
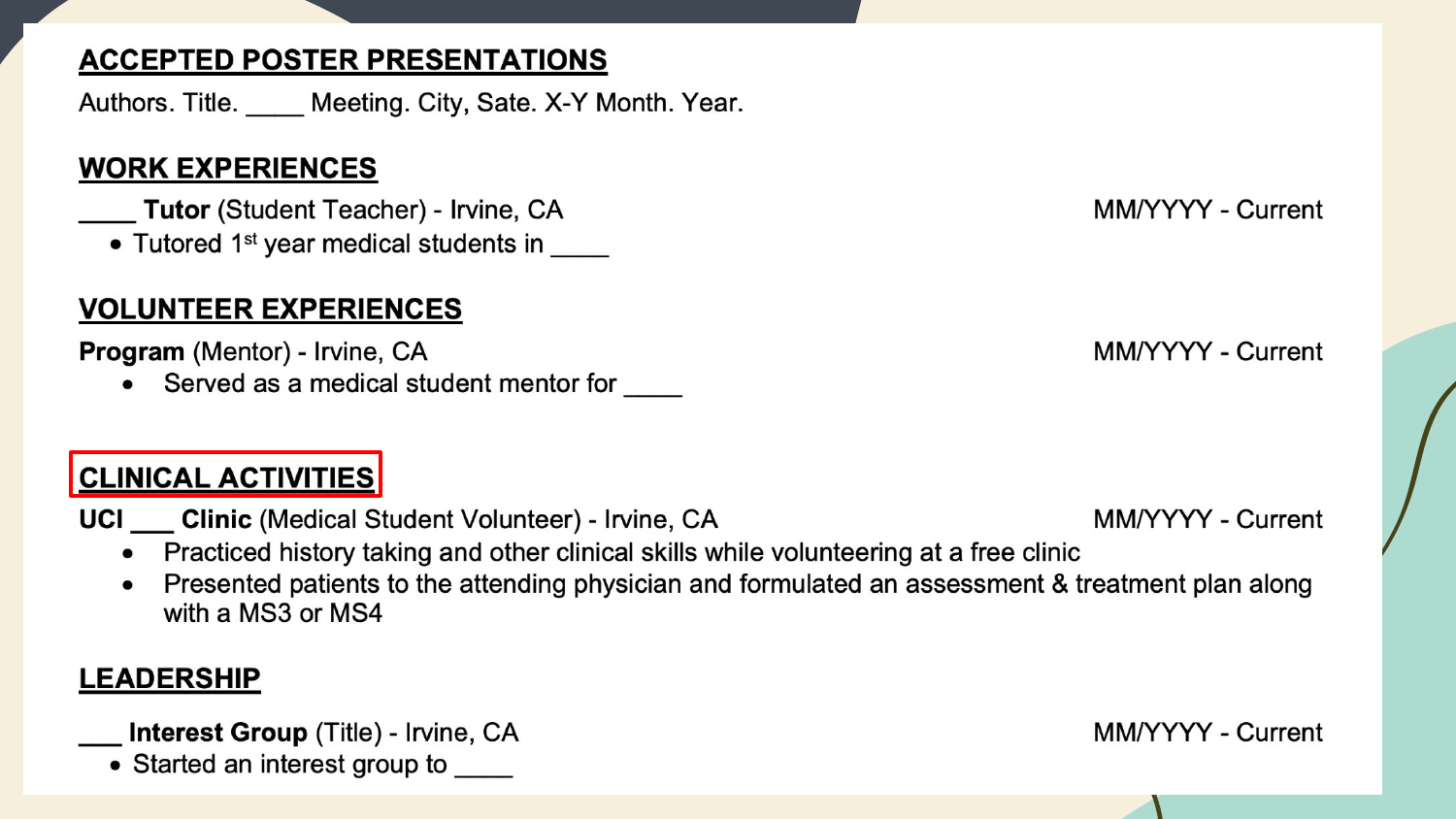

## Slide 27
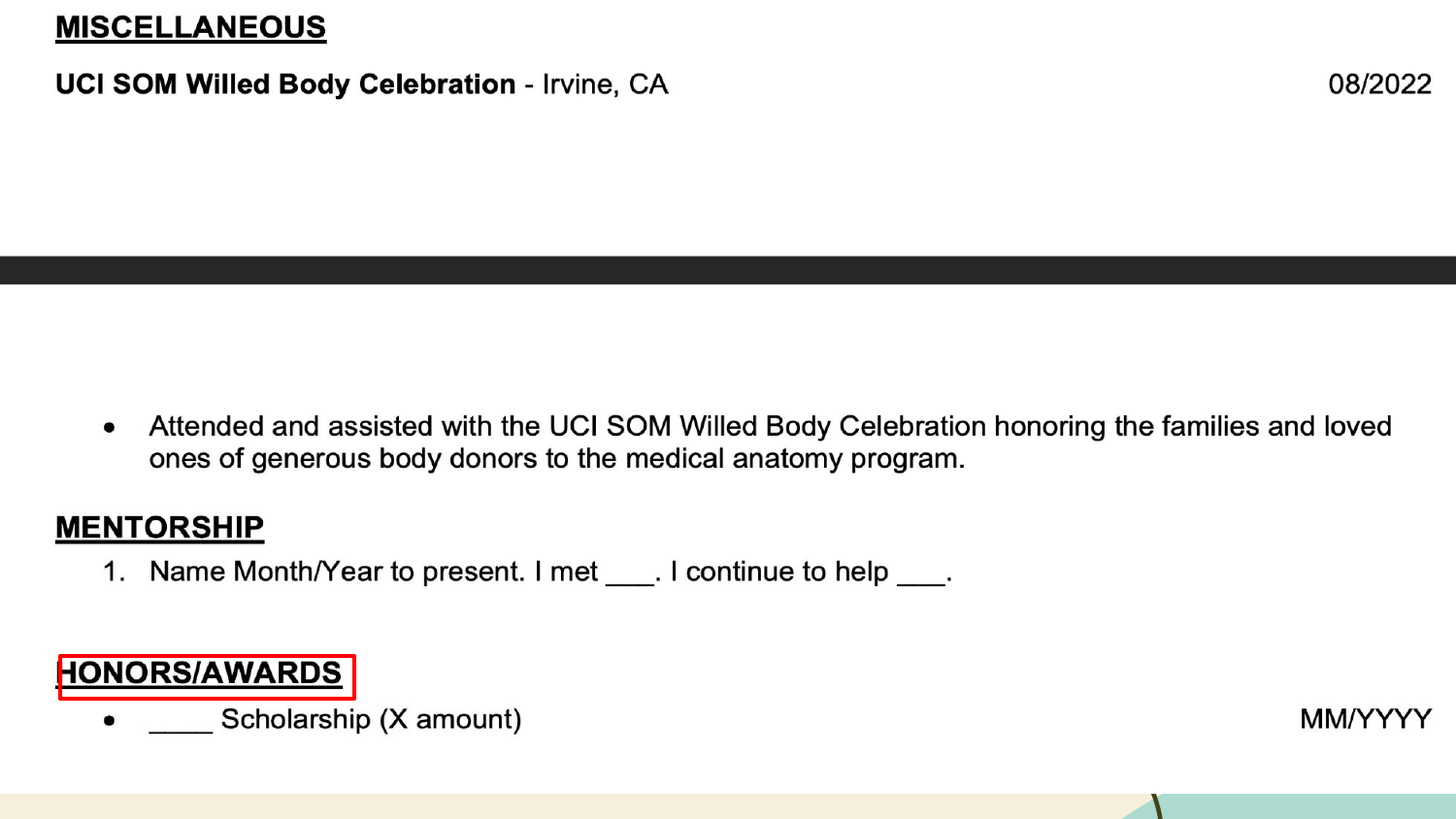

## Slide 28
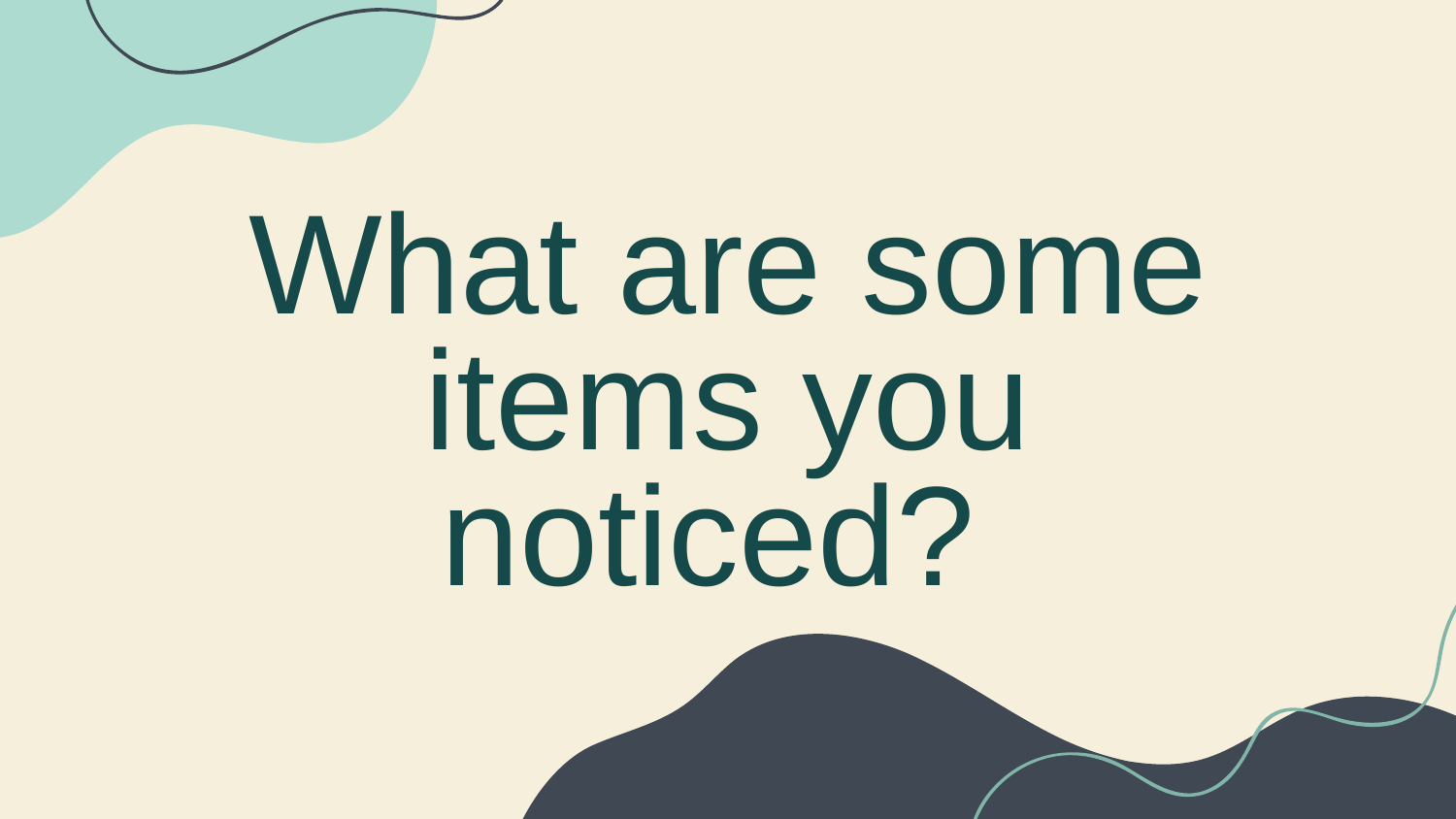

# What are some items you noticed?

## Slide 29
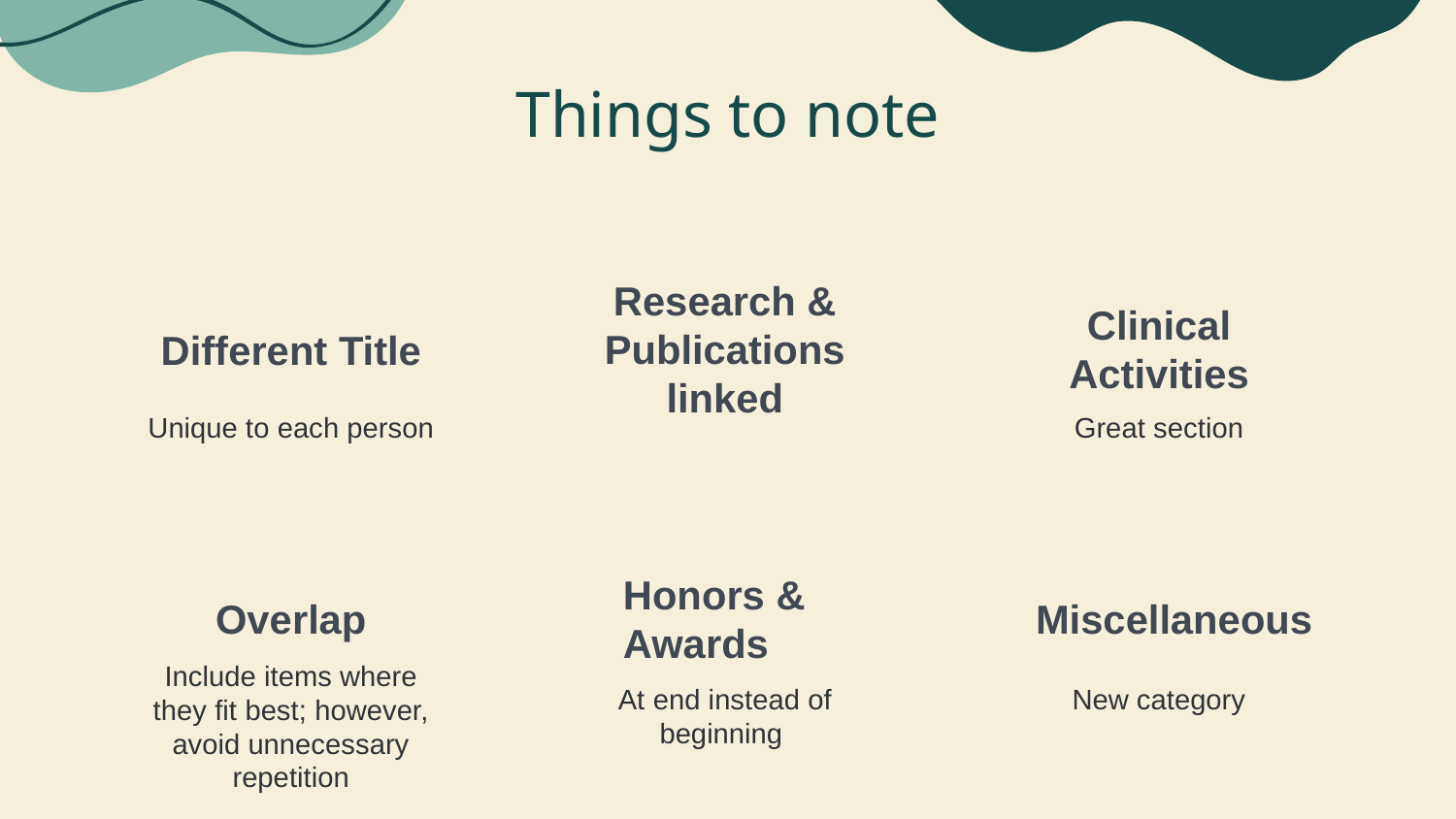

# Things to note
Research & Publications linked
Clinical Activities
Different Title
Great section
Unique to each person
Overlap
Honors & Awards
Miscellaneous
Include items where they fit best; however, avoid unnecessary repetition
At end instead of beginning
New category

## Slide 30
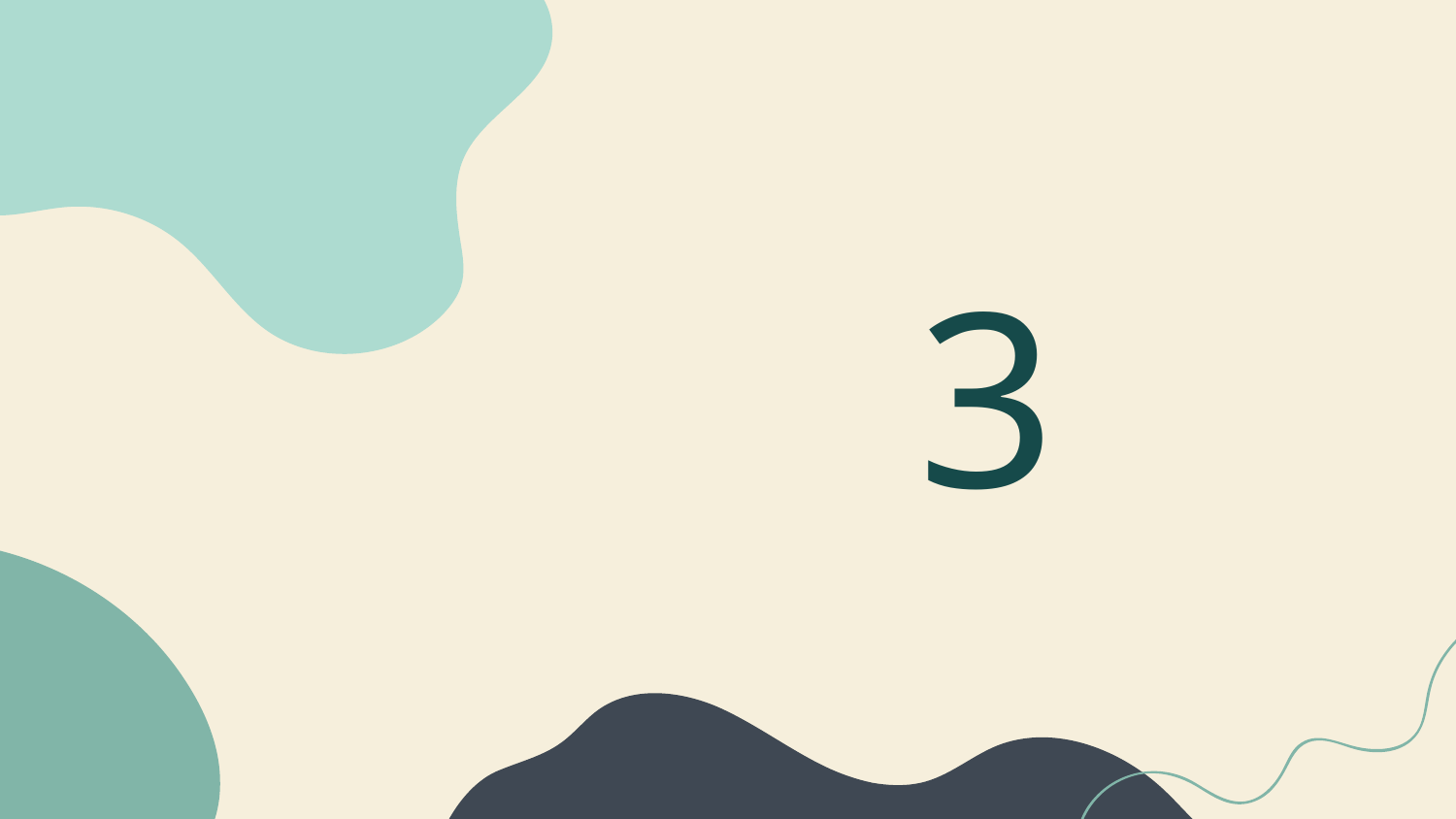

# 3

## Slide 31
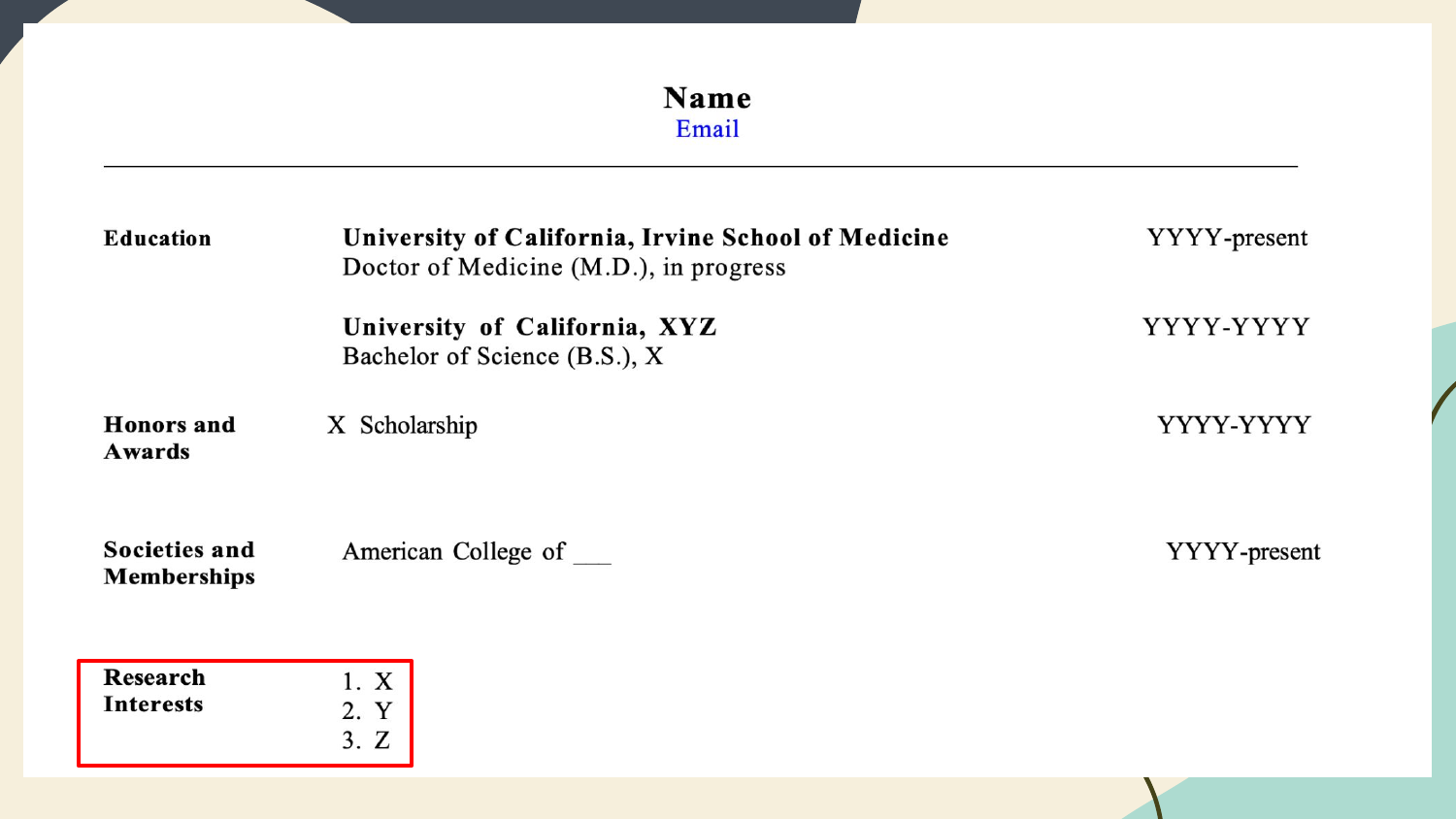

## Slide 32
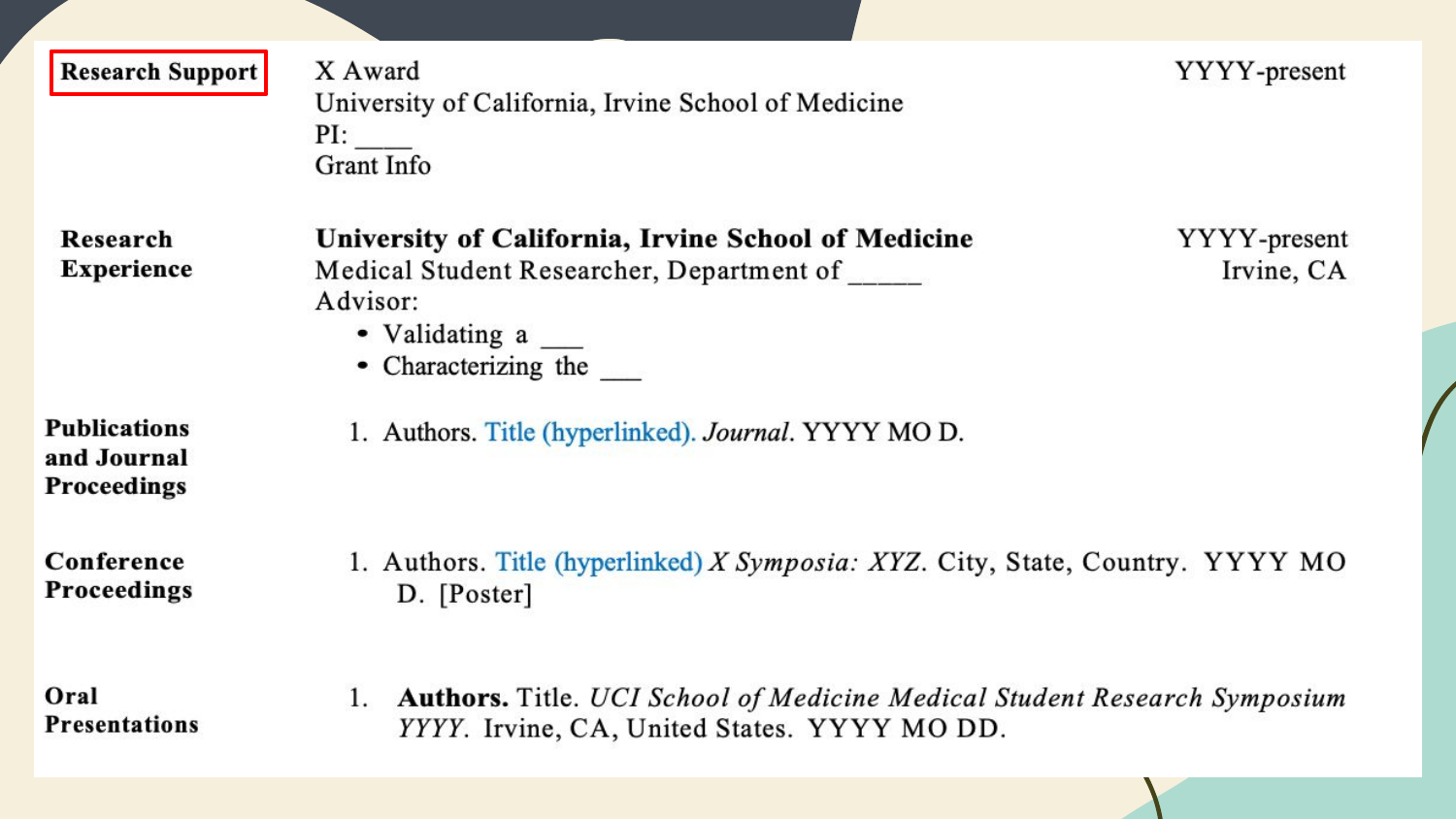

## Slide 33
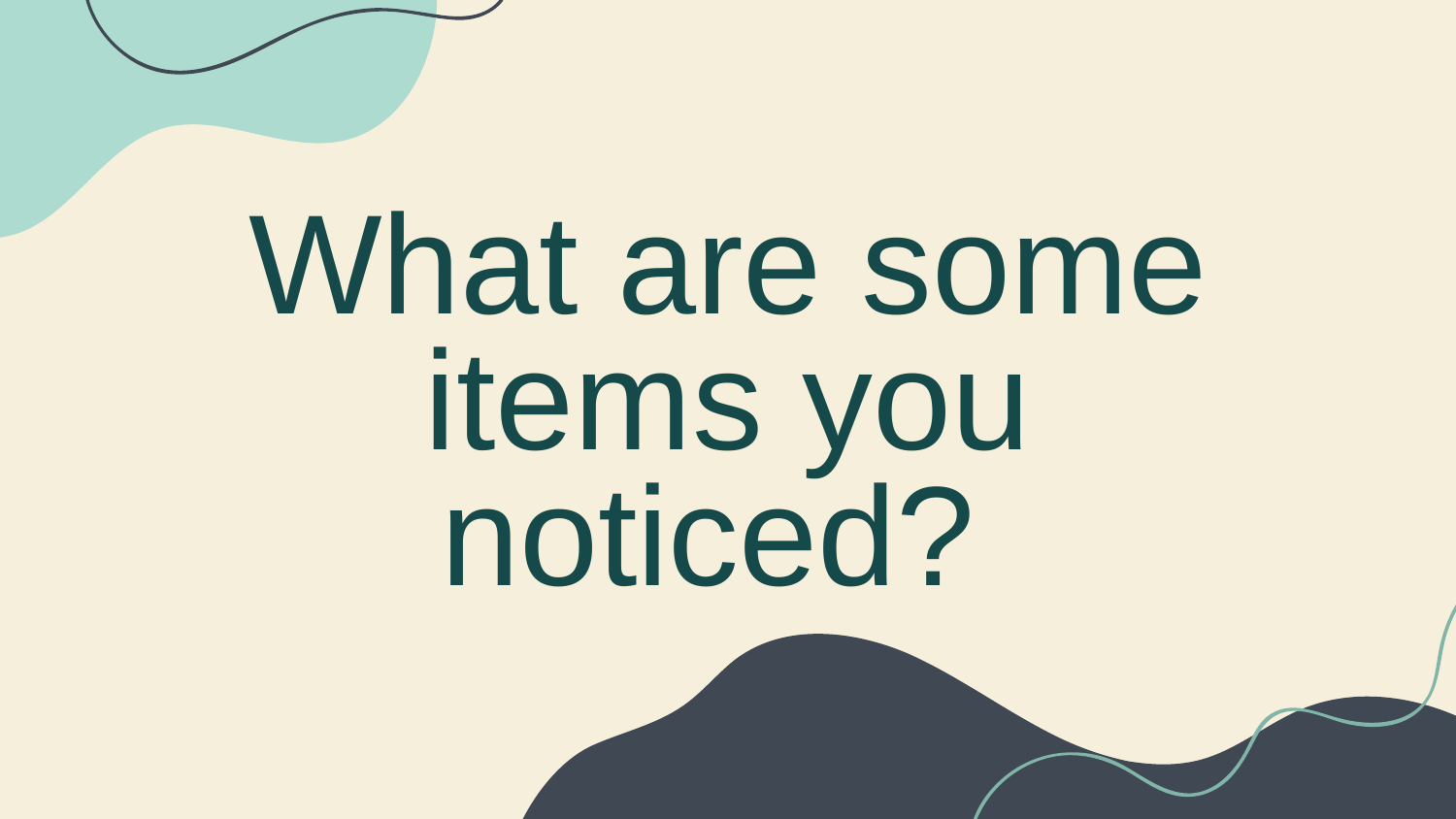

# What are some items you noticed?

## Slide 34
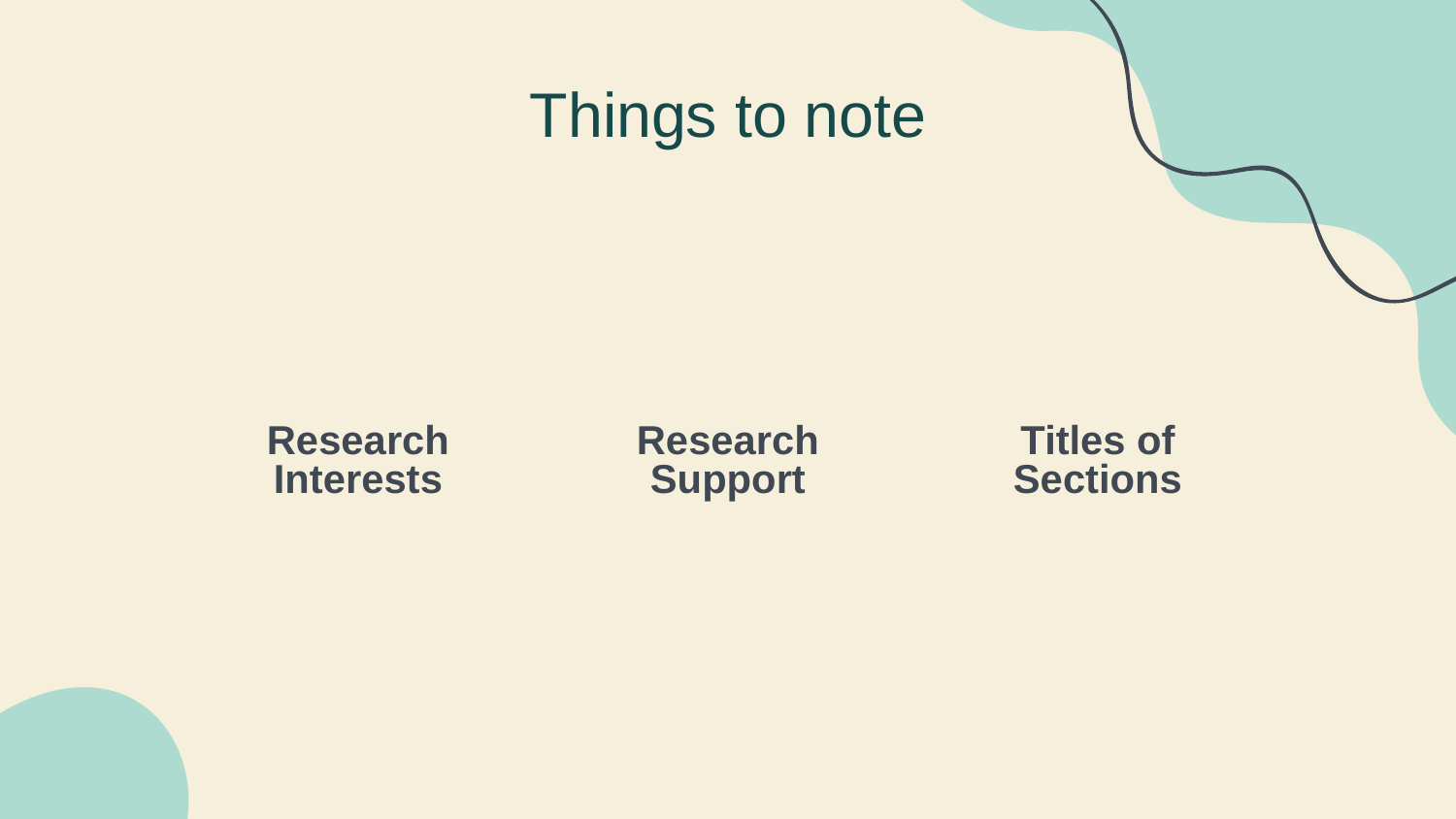

# Things to note
Research Interests
Research Support
Titles of Sections

## Slide 35
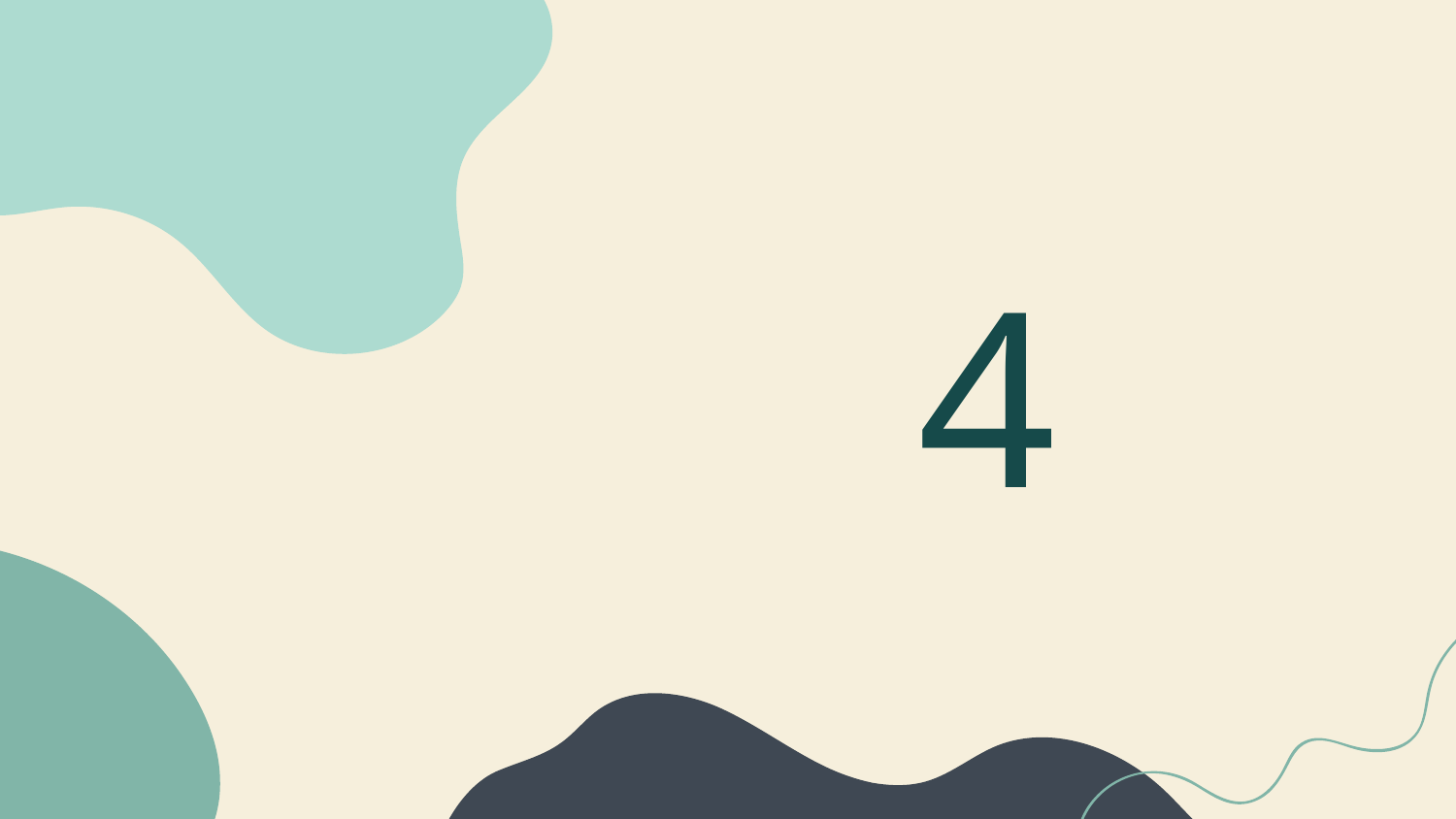

# 4

## Slide 36
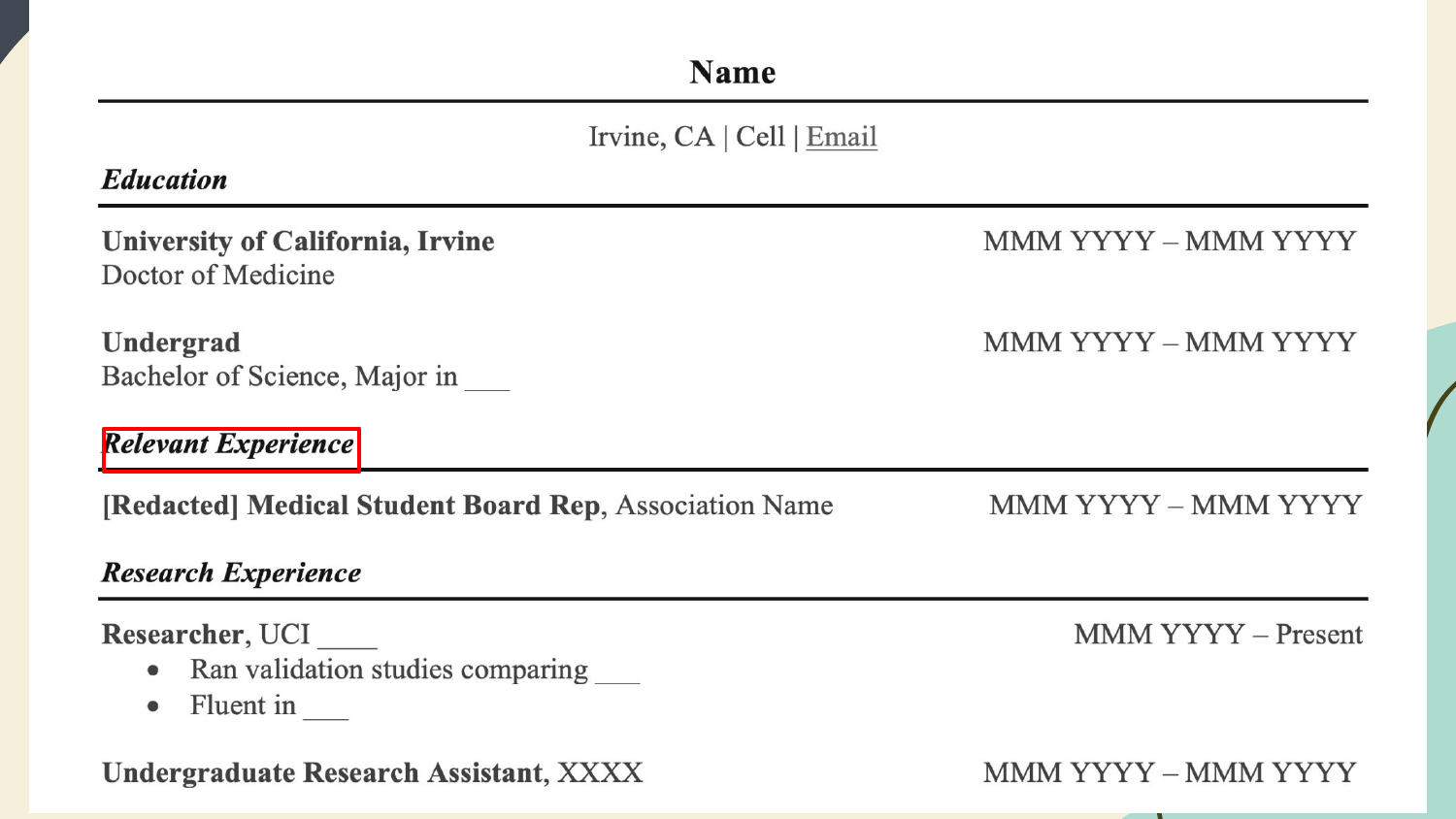

## Slide 37
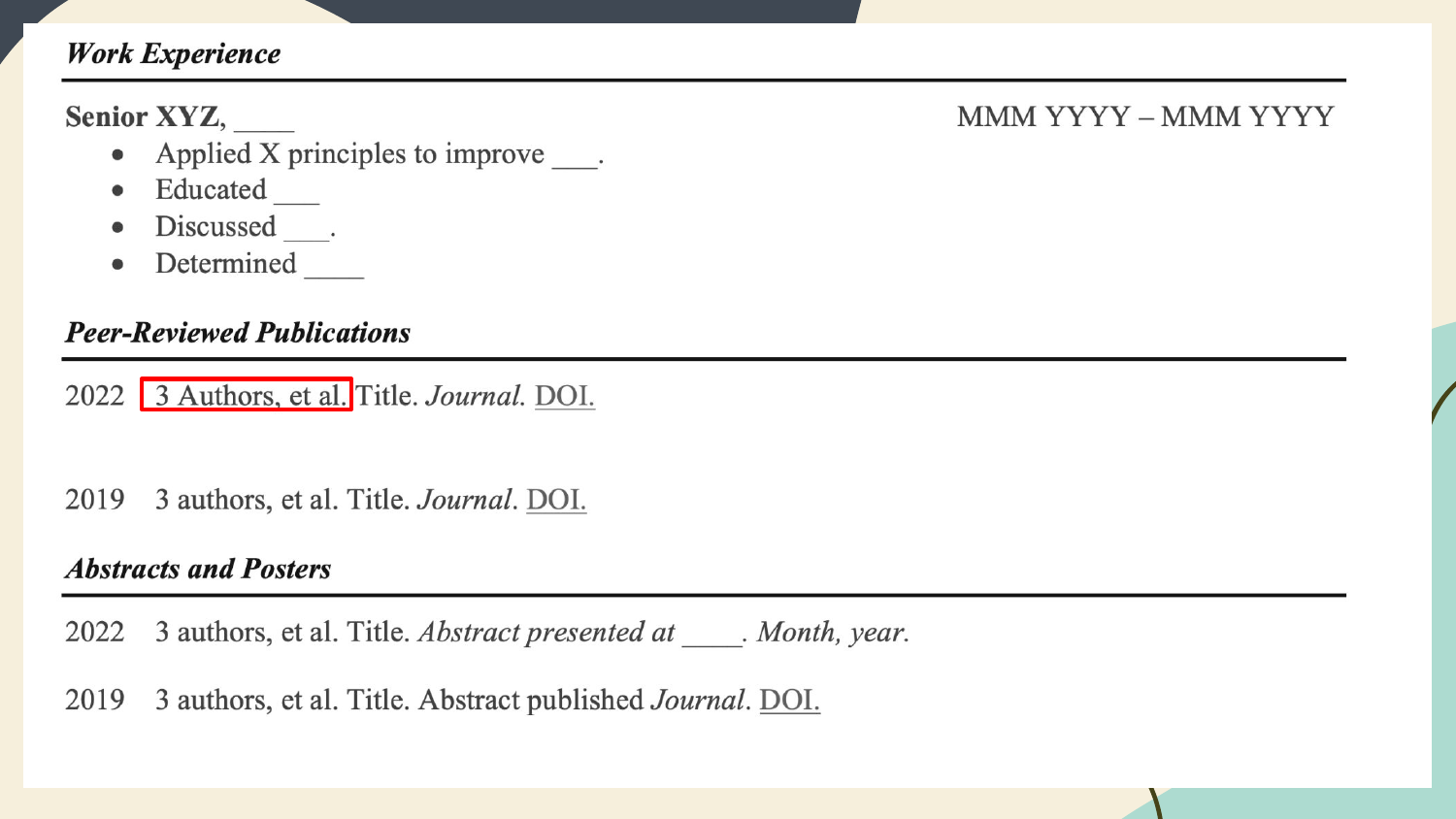

## Slide 38
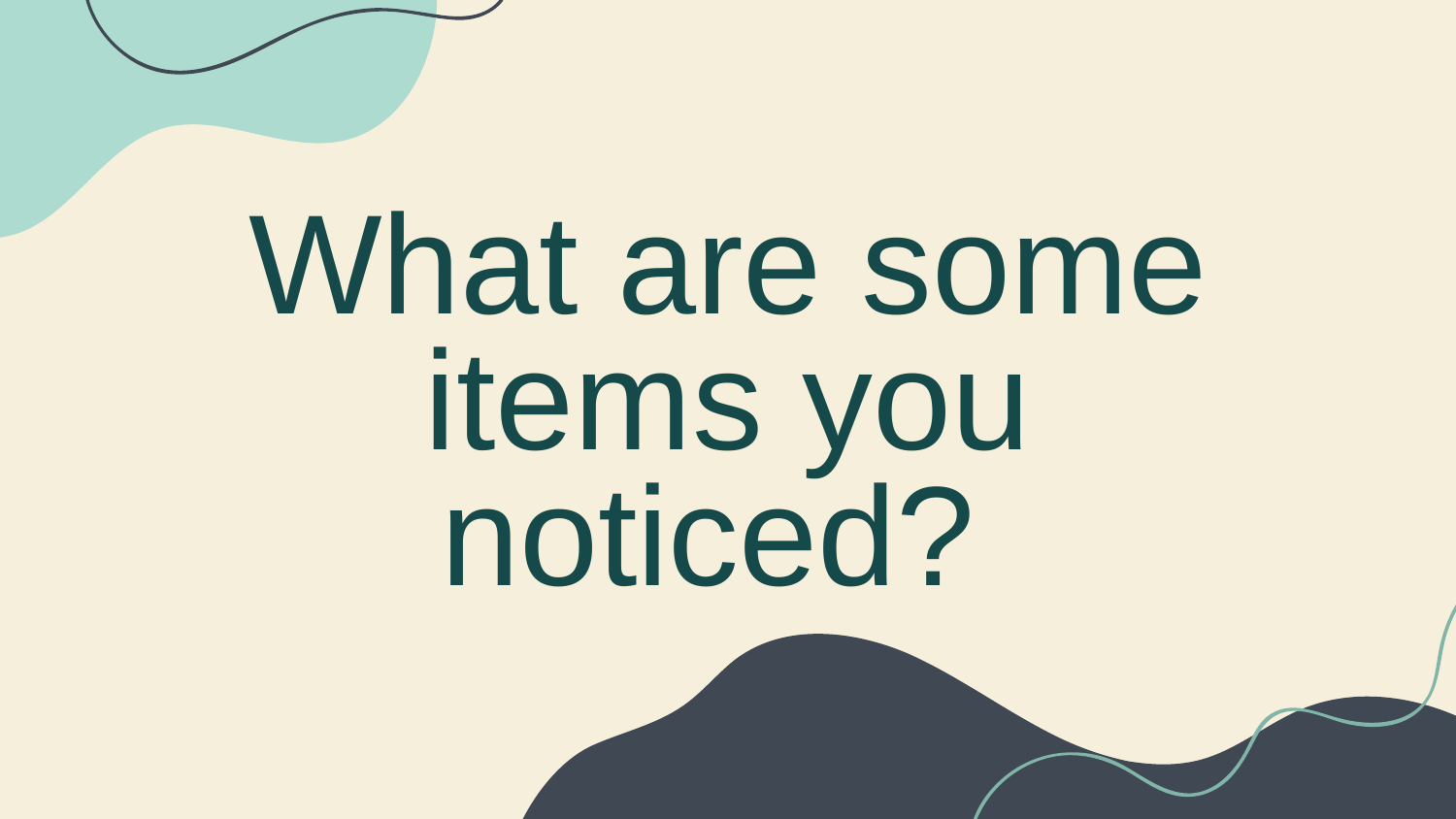

# What are some items you noticed?

## Slide 39
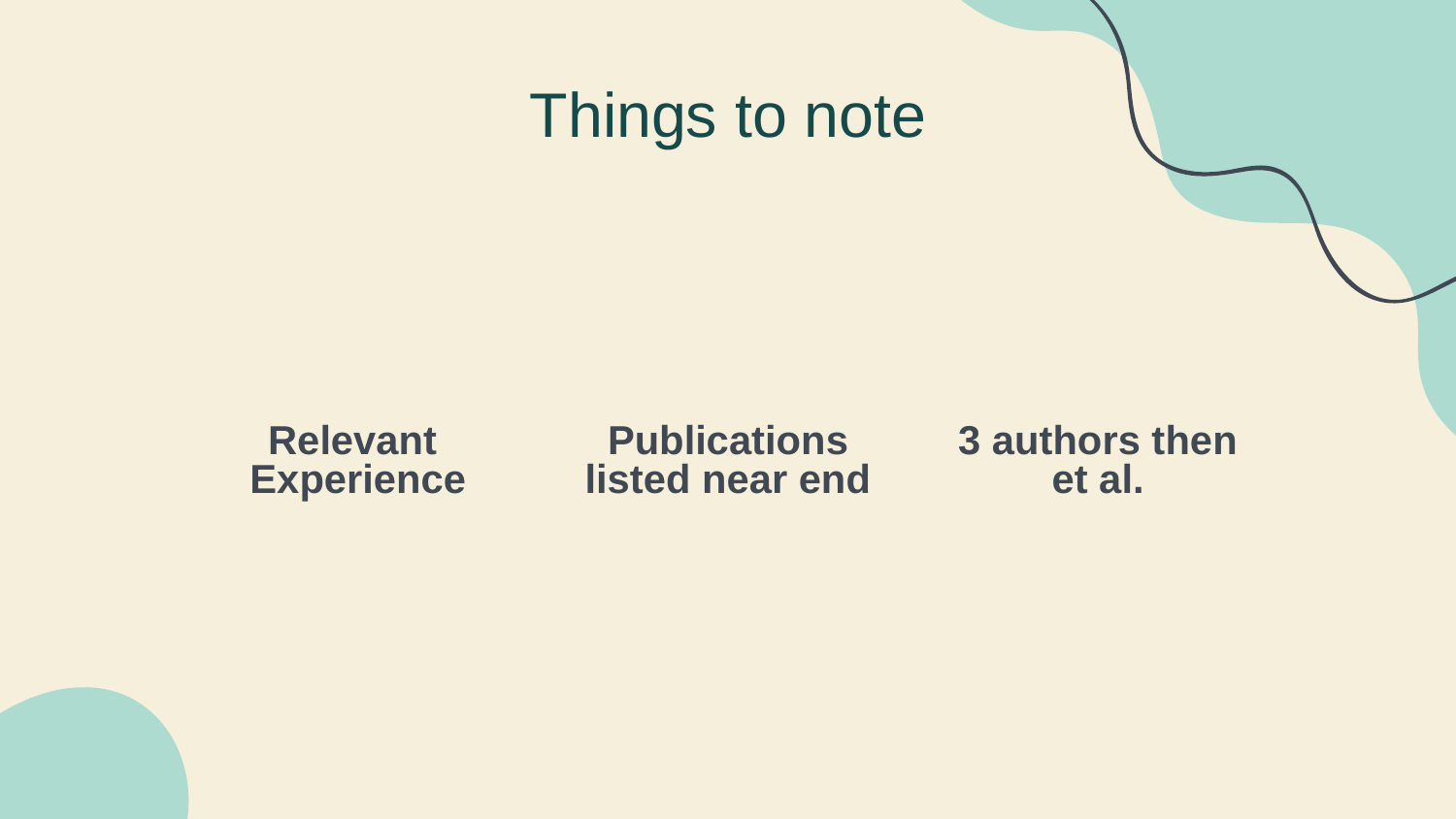

# Things to note
Relevant Experience
Publications listed near end
3 authors then et al.

## Slide 40
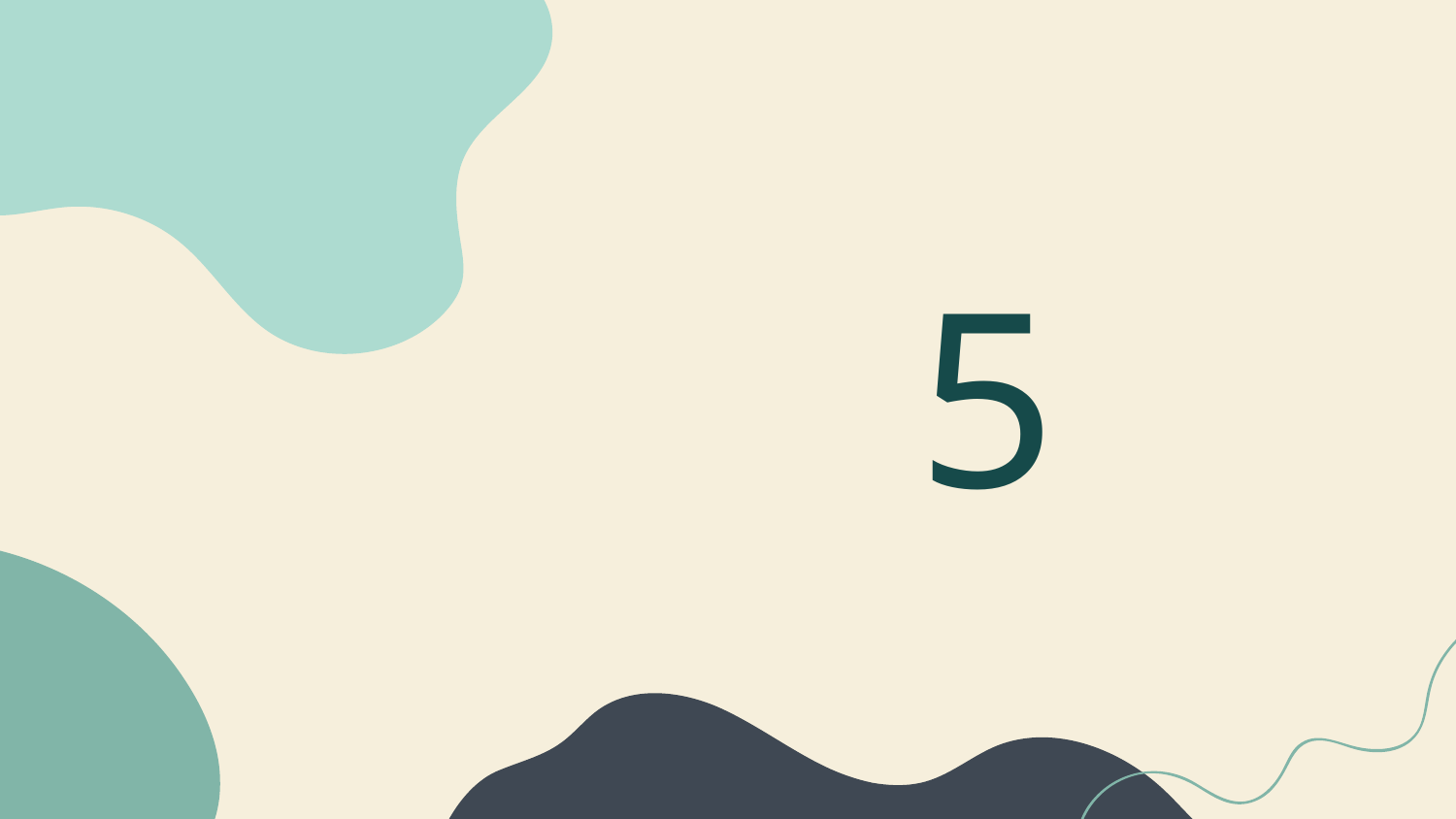

# 5

## Slide 41
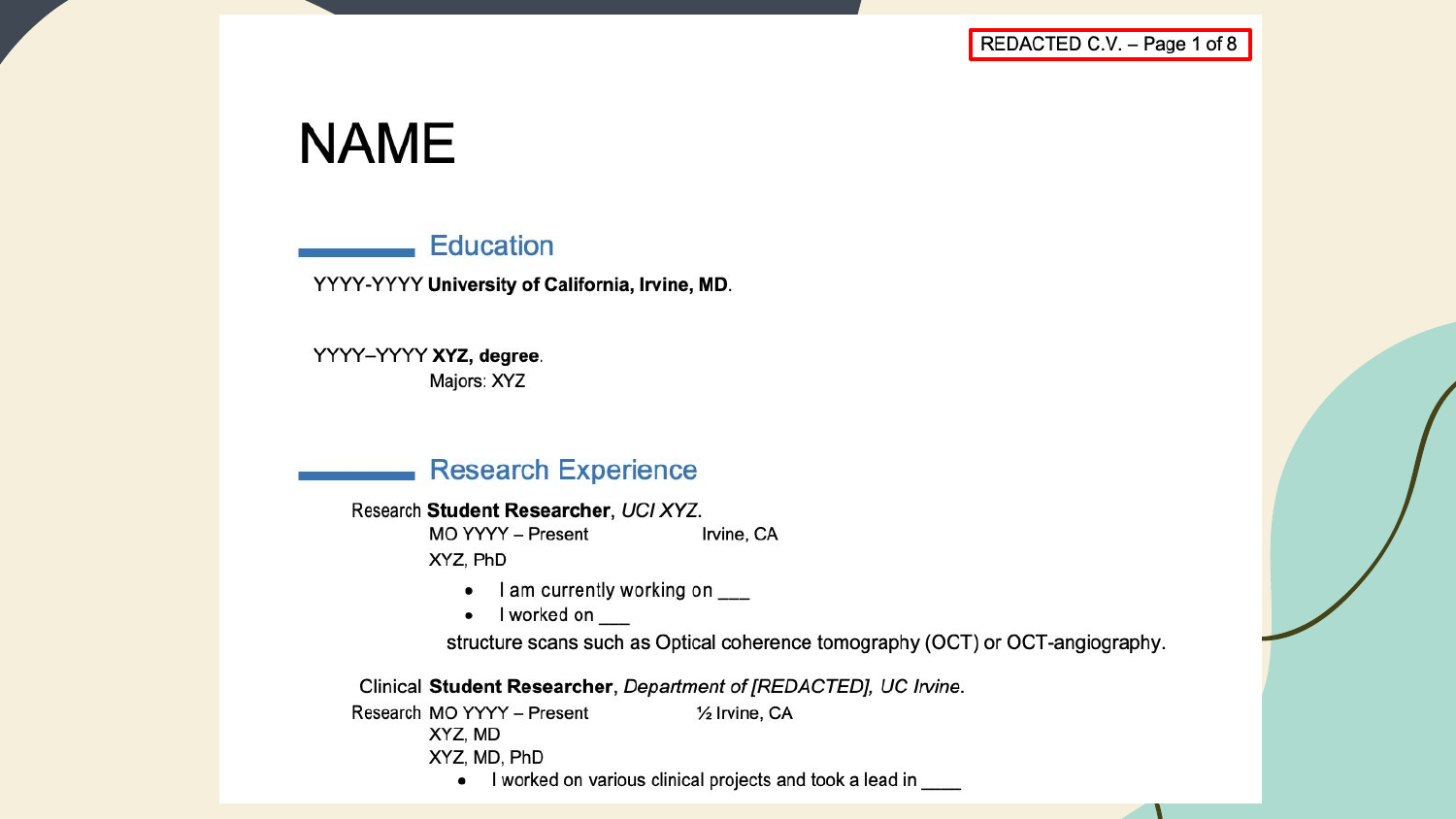

## Slide 42
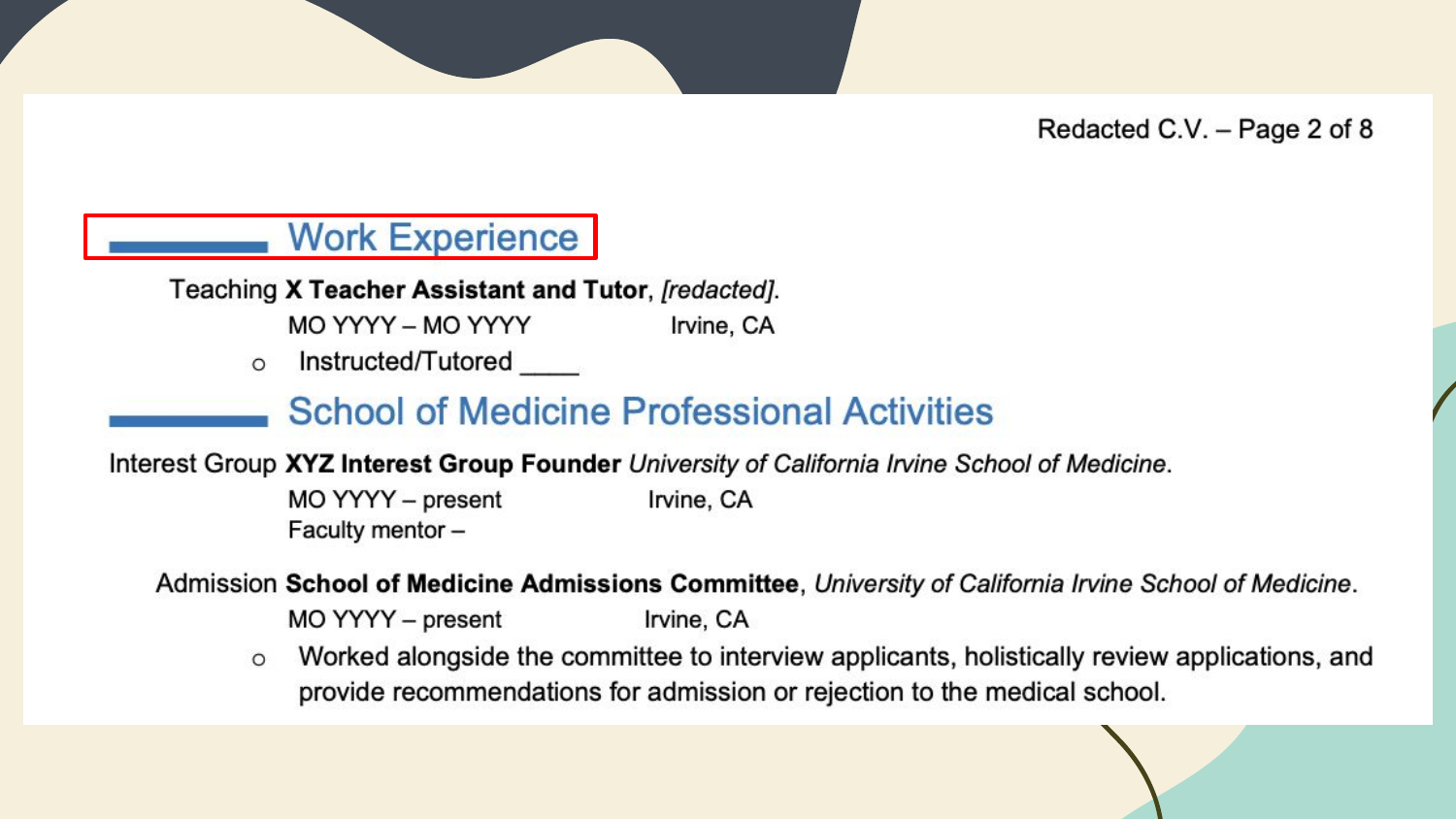

## Slide 43
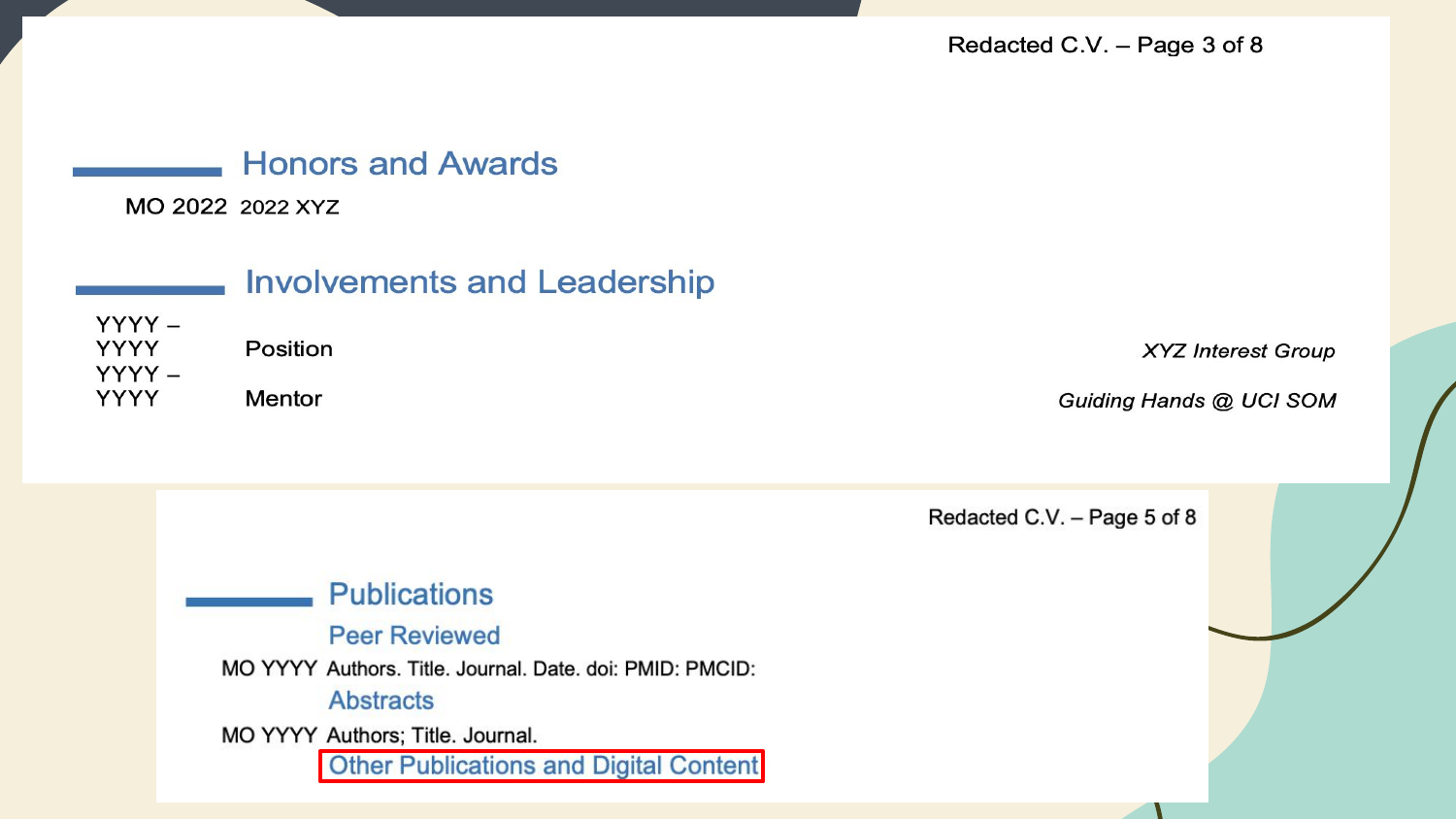

## Slide 44
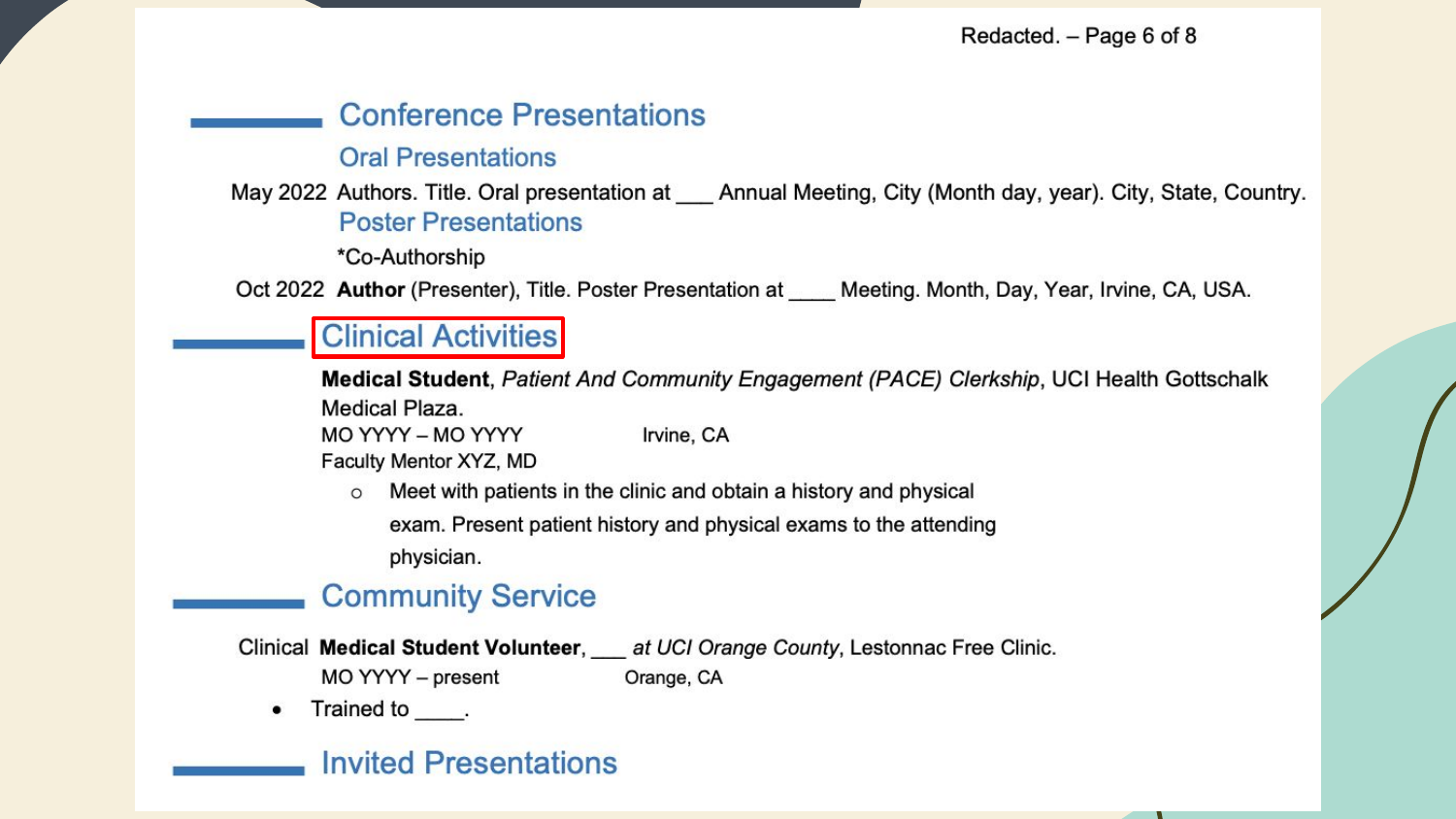

## Slide 45
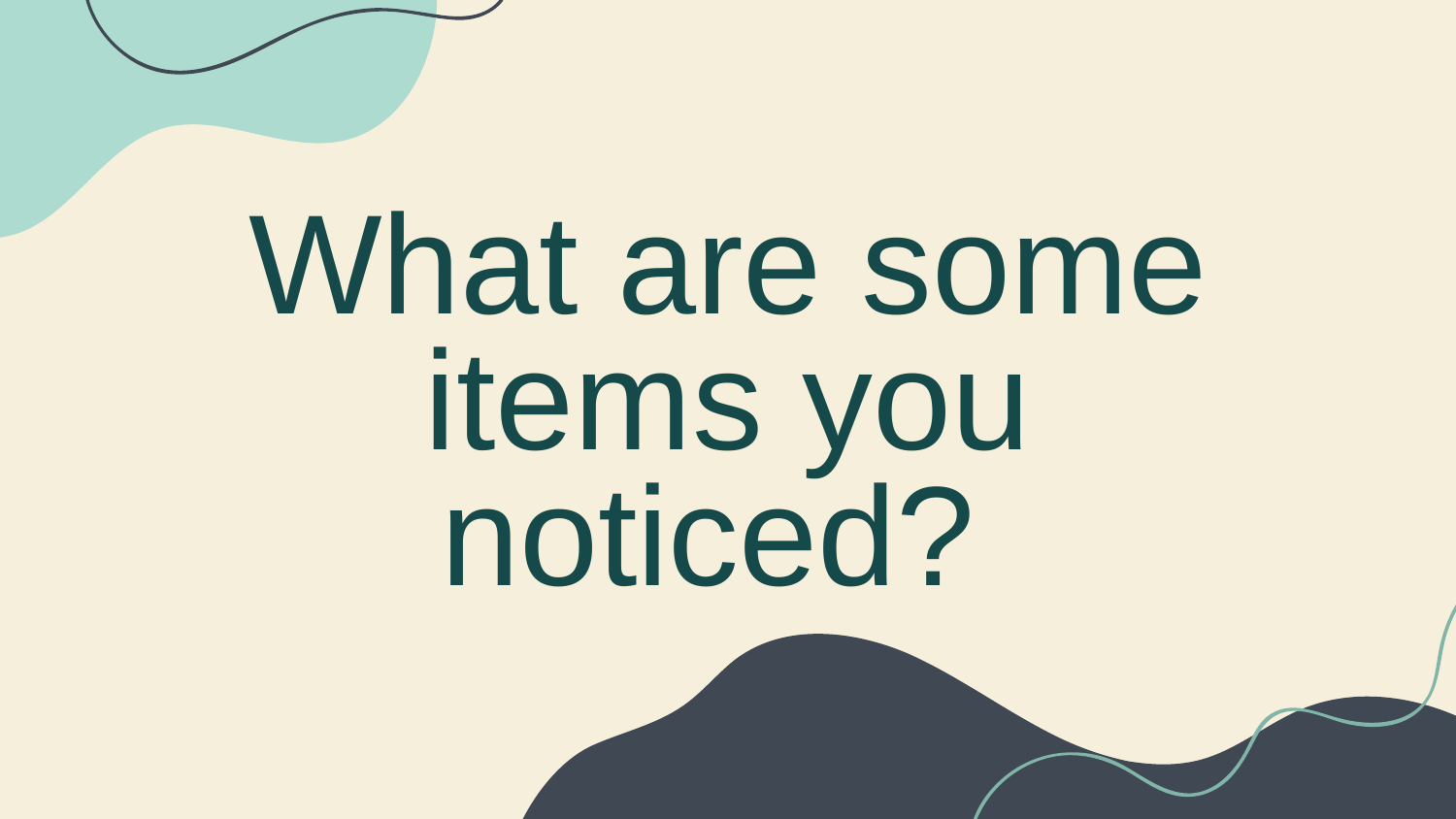

# What are some items you noticed?

## Slide 46
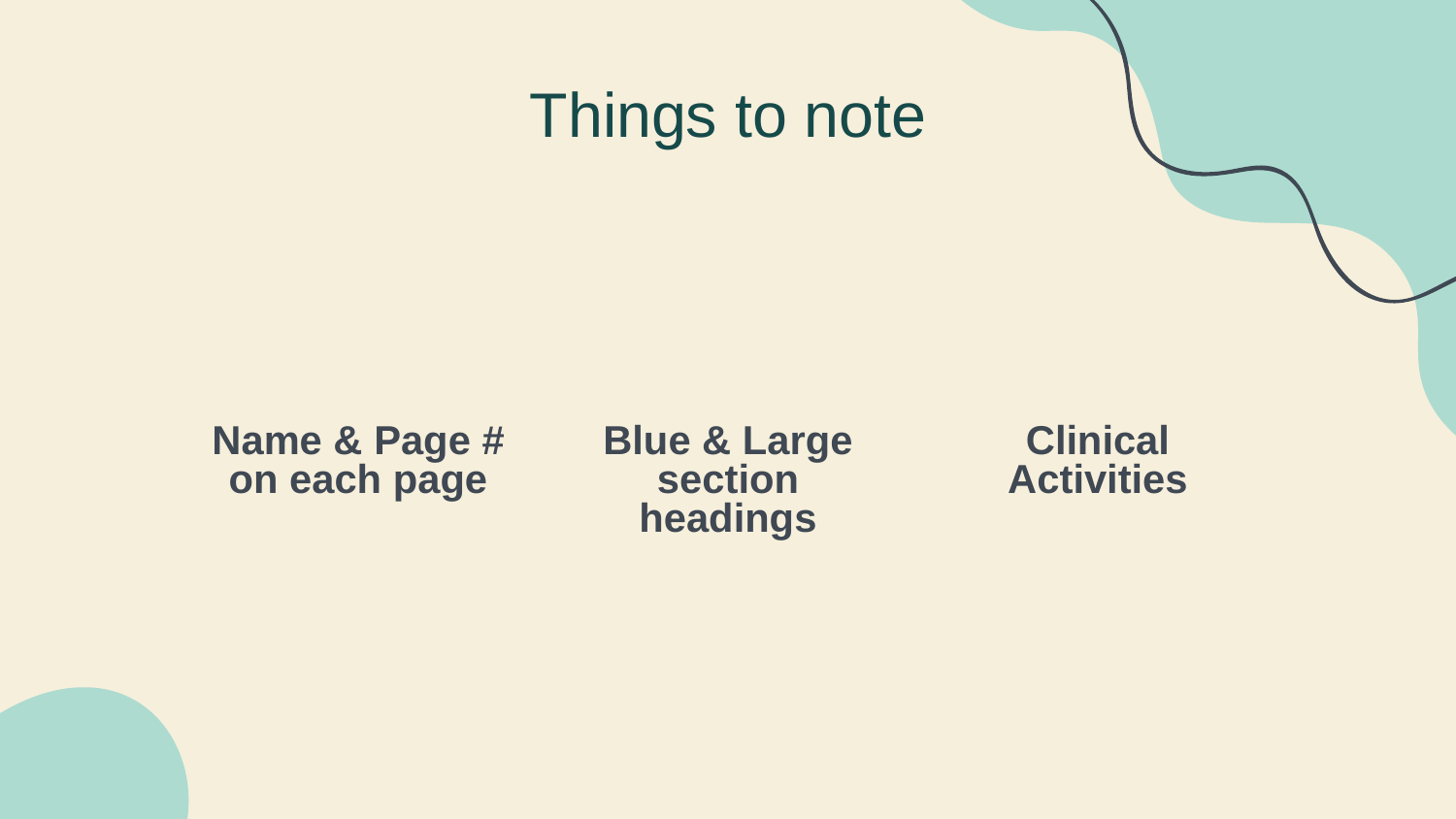

# Things to note
Name & Page # on each page
Blue & Large section headings
Clinical Activities

## Slide 47
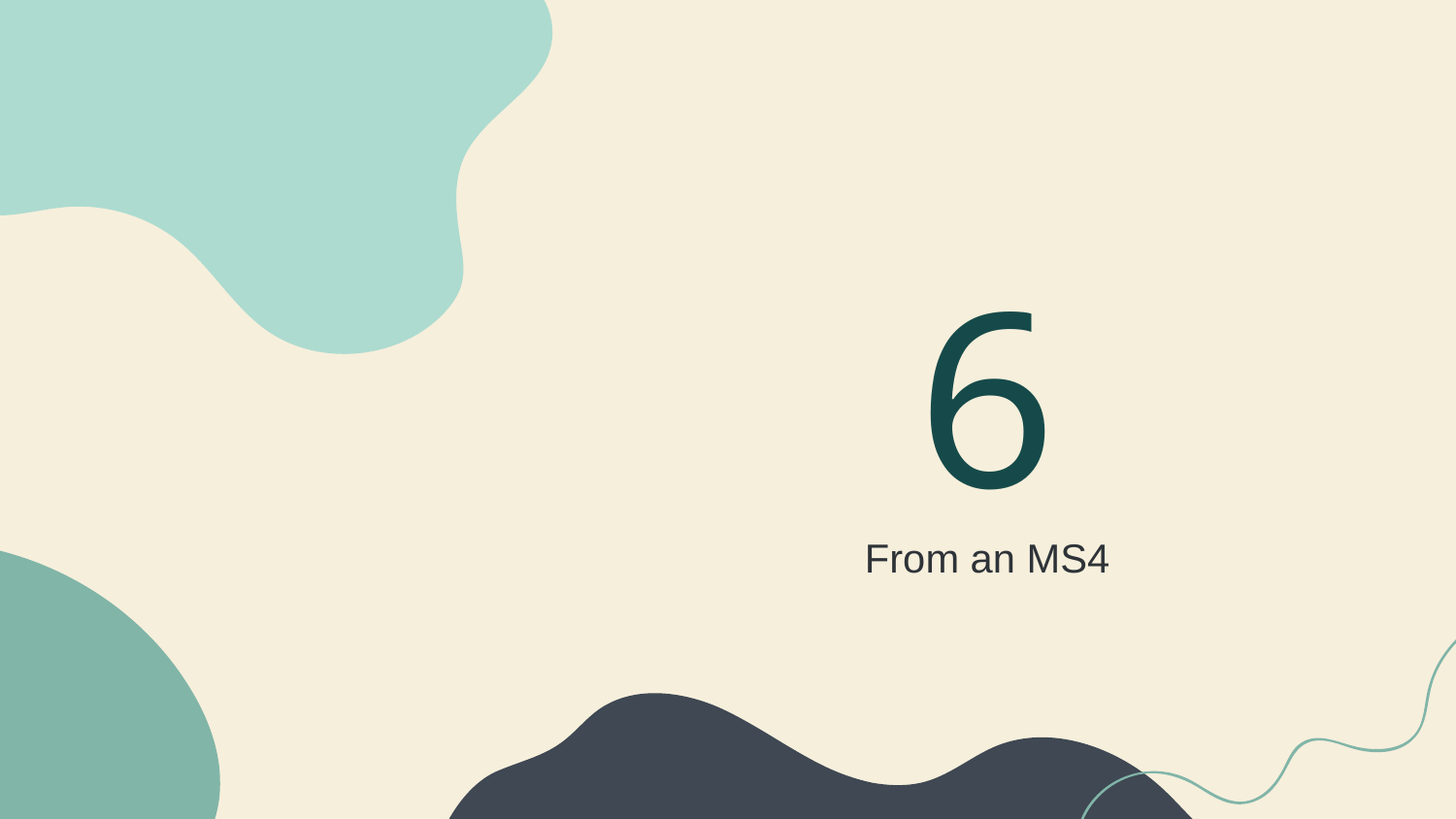

# 6
From an MS4

## Slide 48
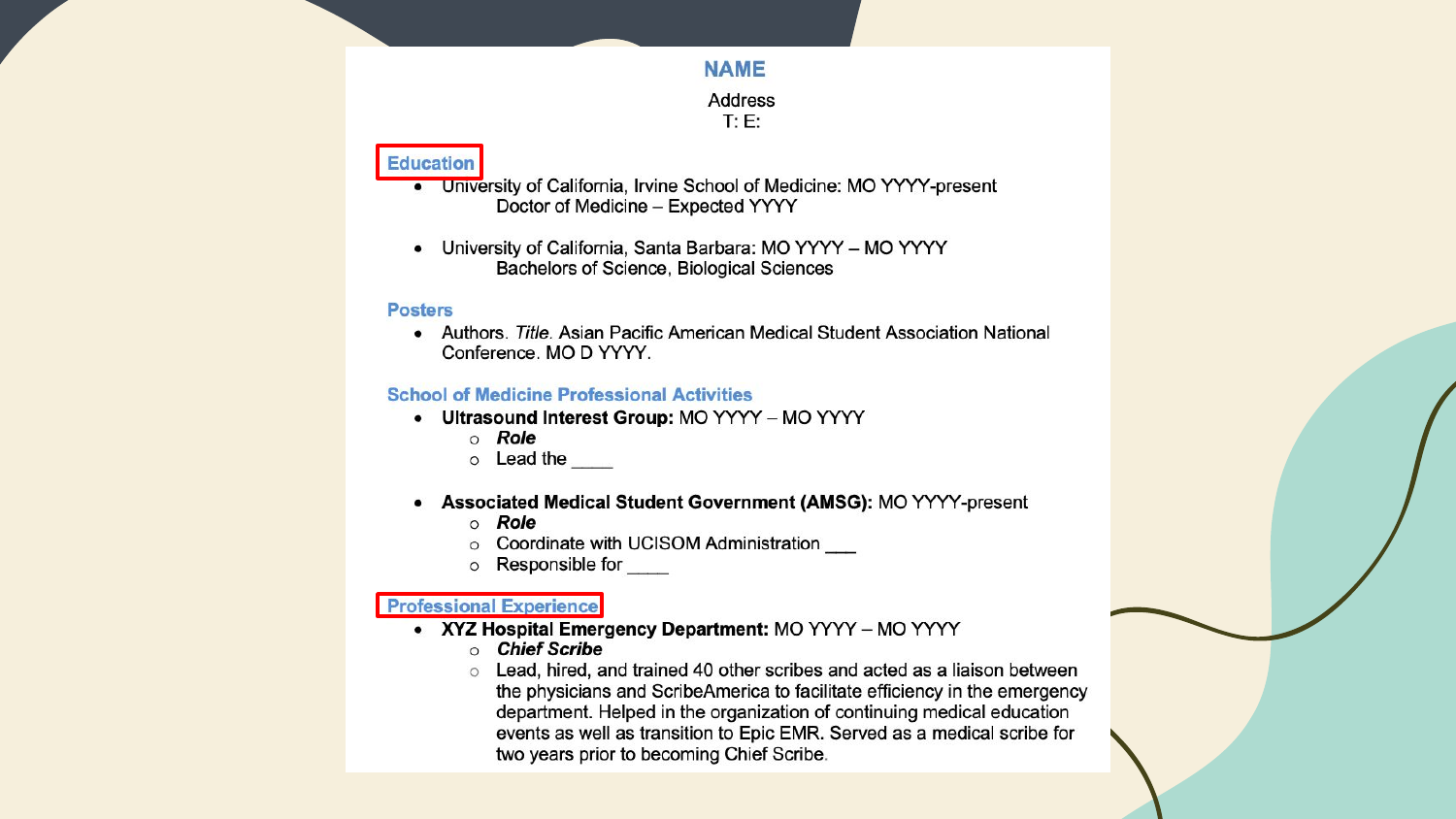

## Slide 49
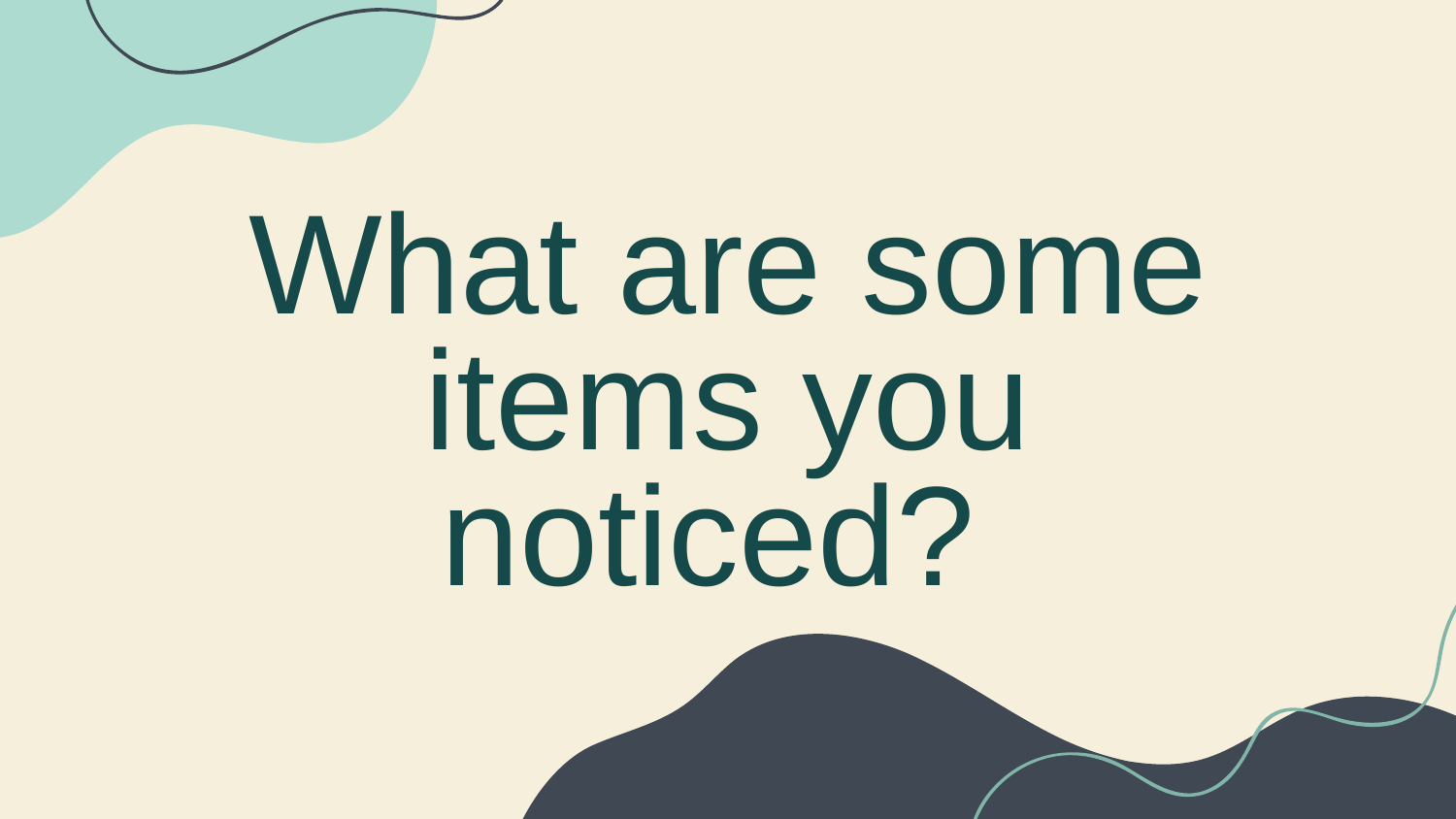

# What are some items you noticed?

## Slide 50
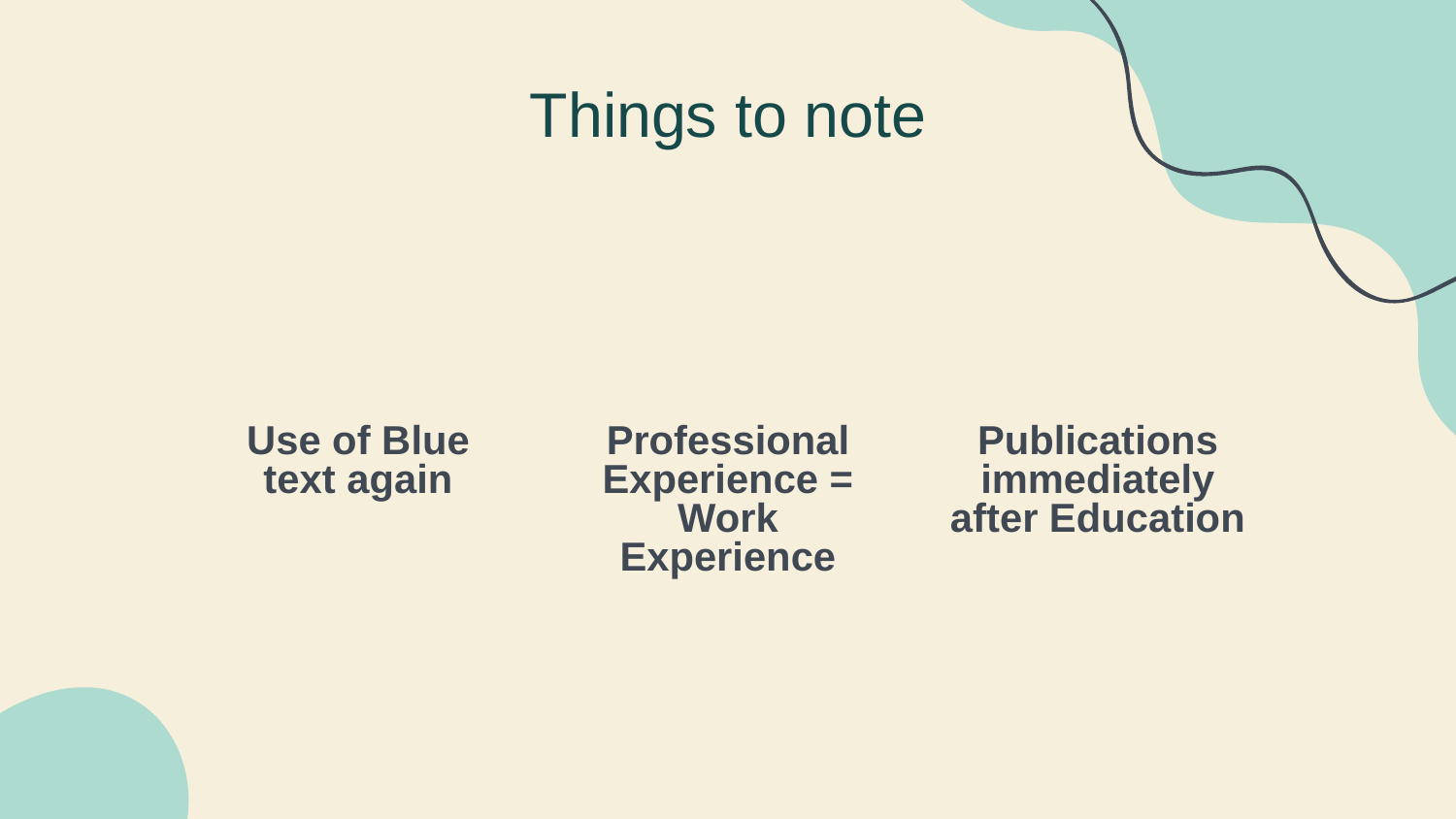

# Things to note
Use of Blue text again
Professional Experience = Work Experience
Publications immediately after Education

## Slide 51
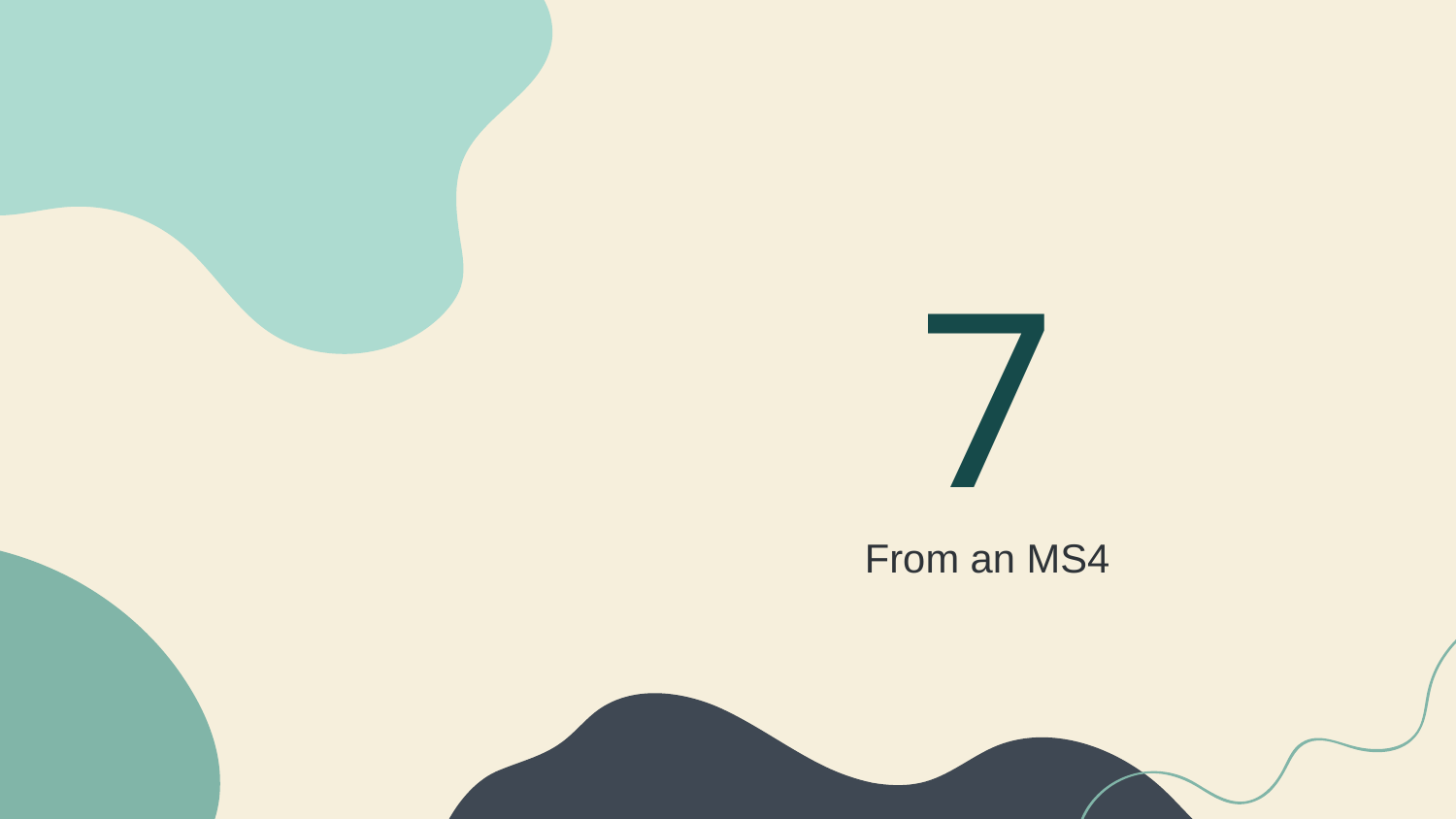

# 7
From an MS4

## Slide 52
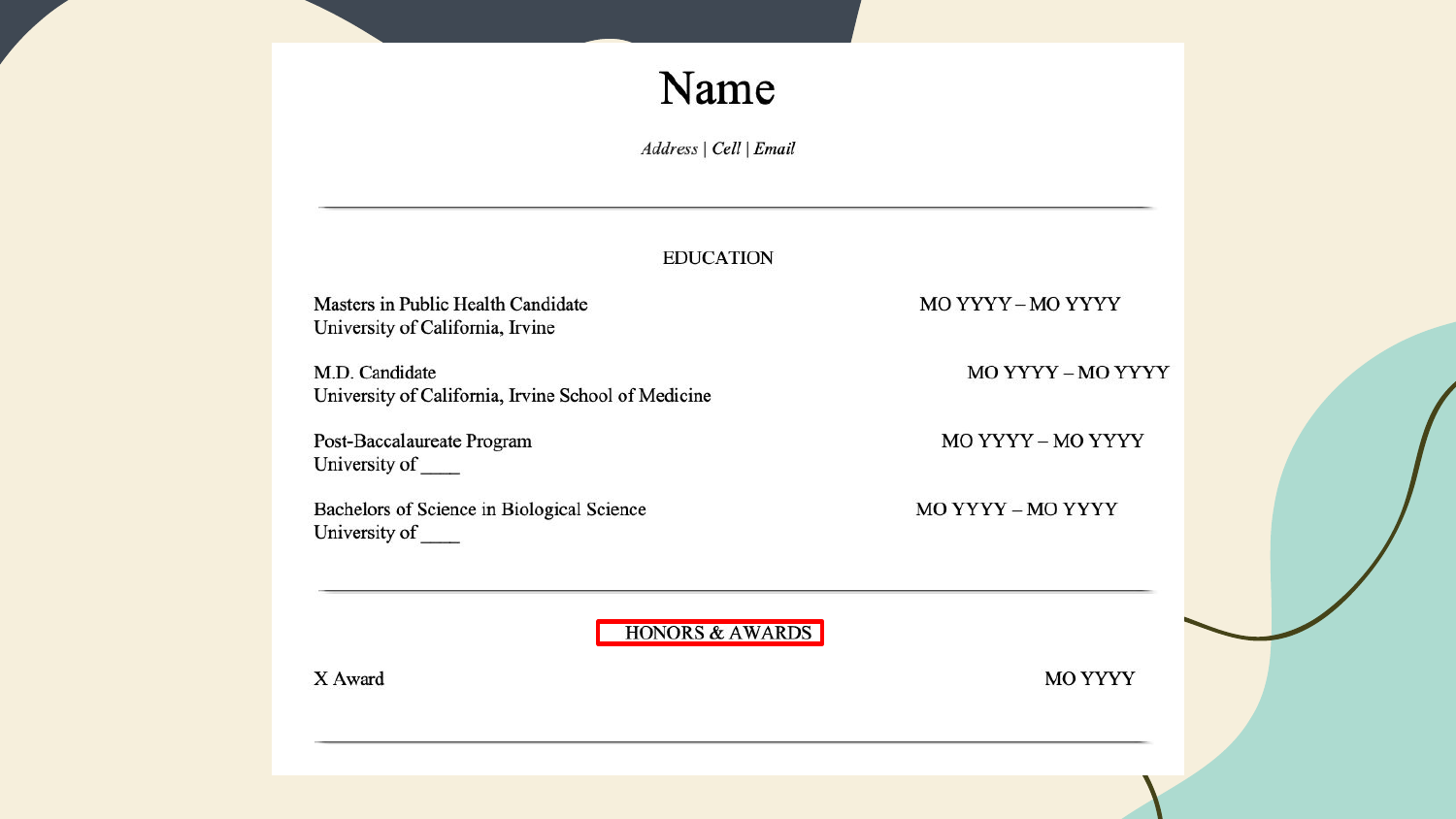

## Slide 53
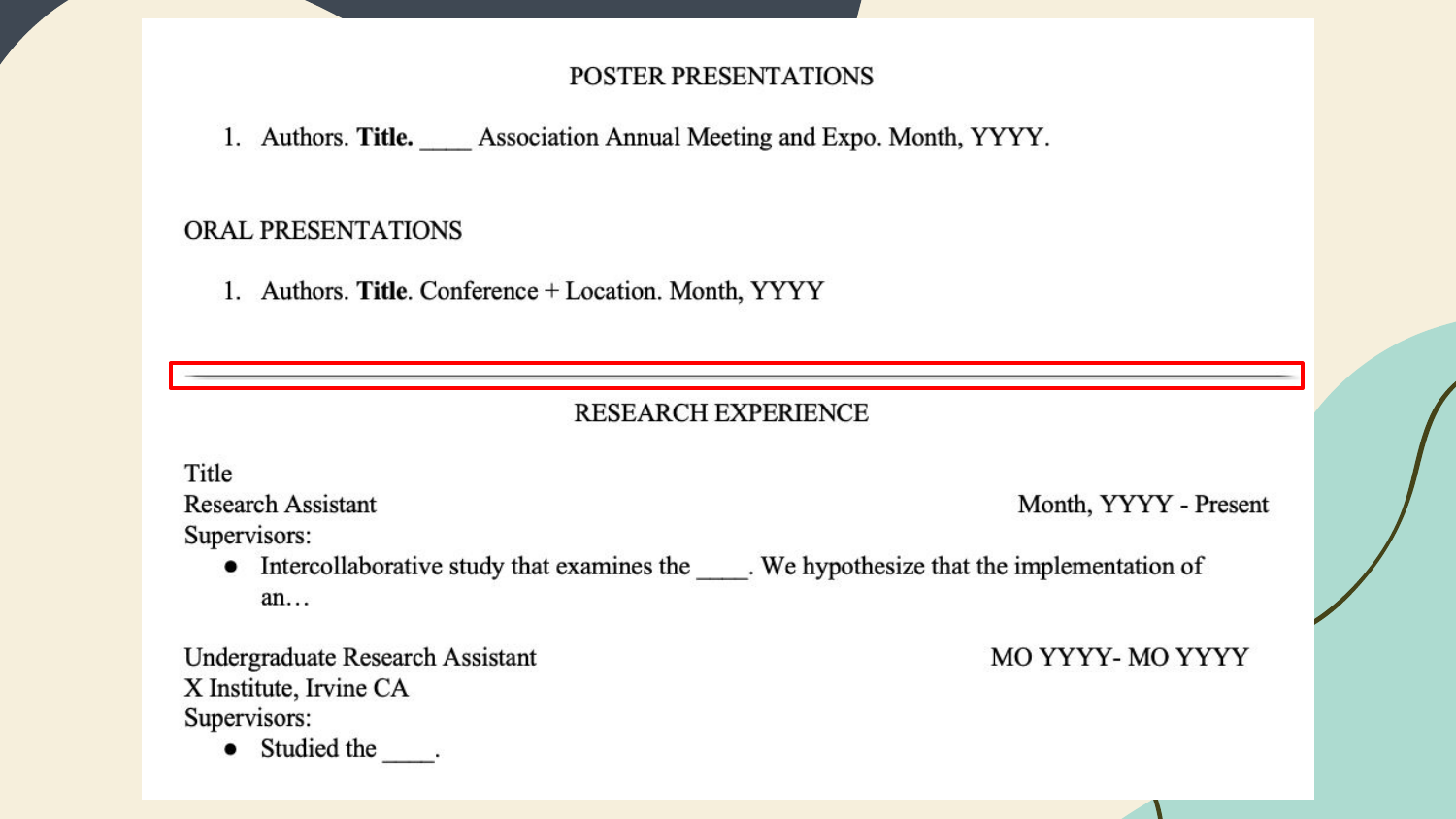

## Slide 54
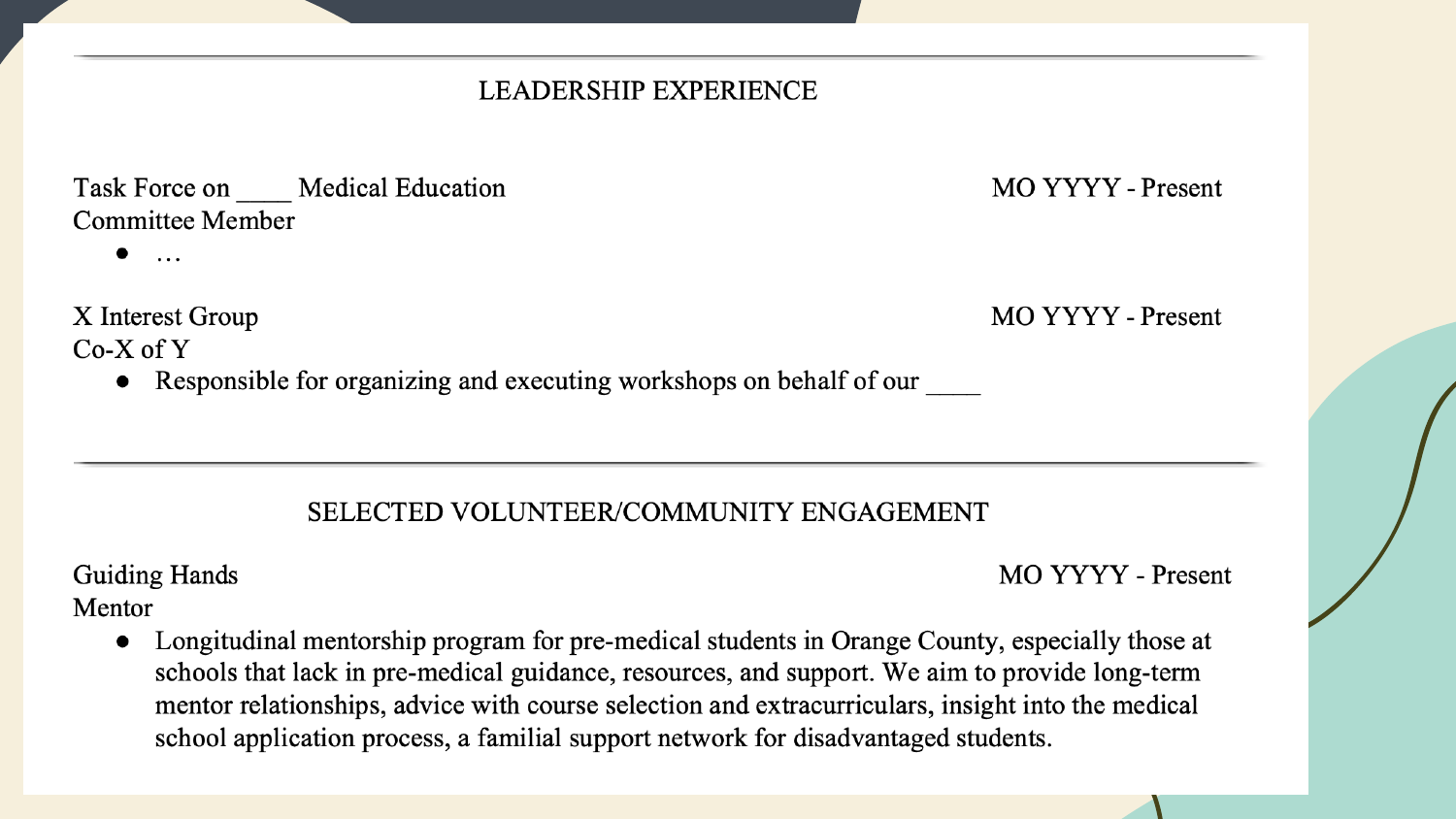

## Slide 55
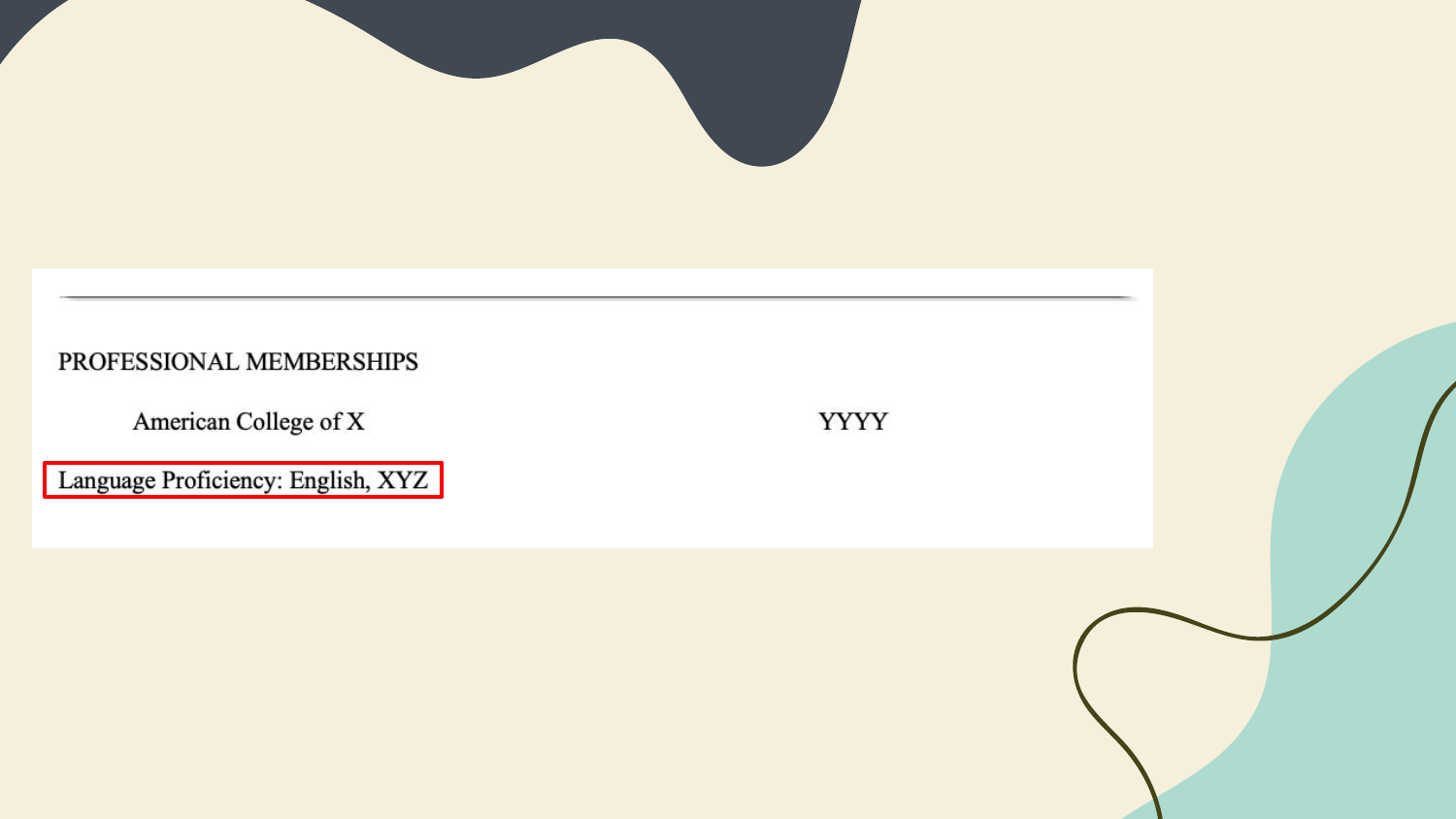

## Slide 56
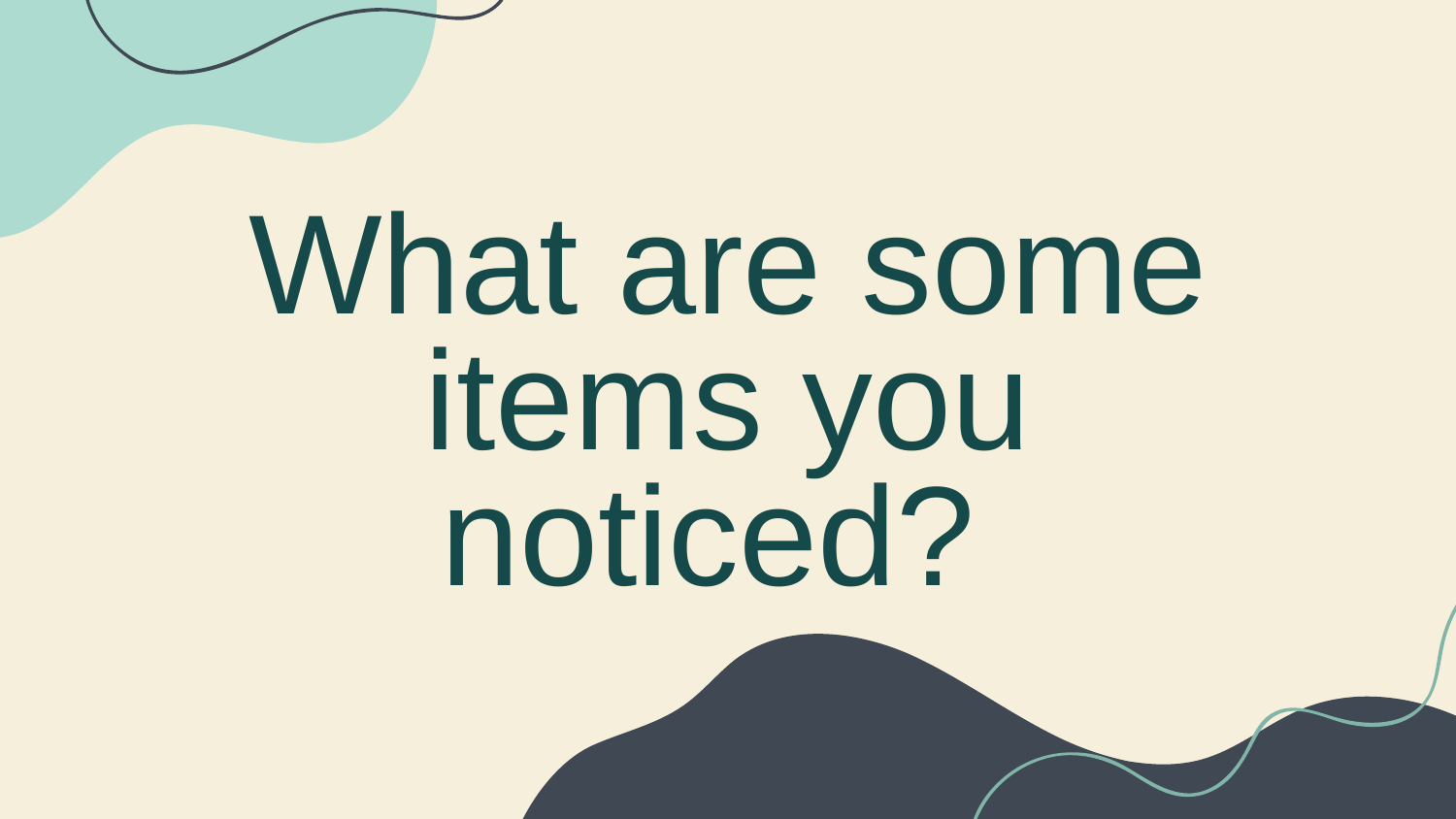

# What are some items you noticed?

## Slide 57
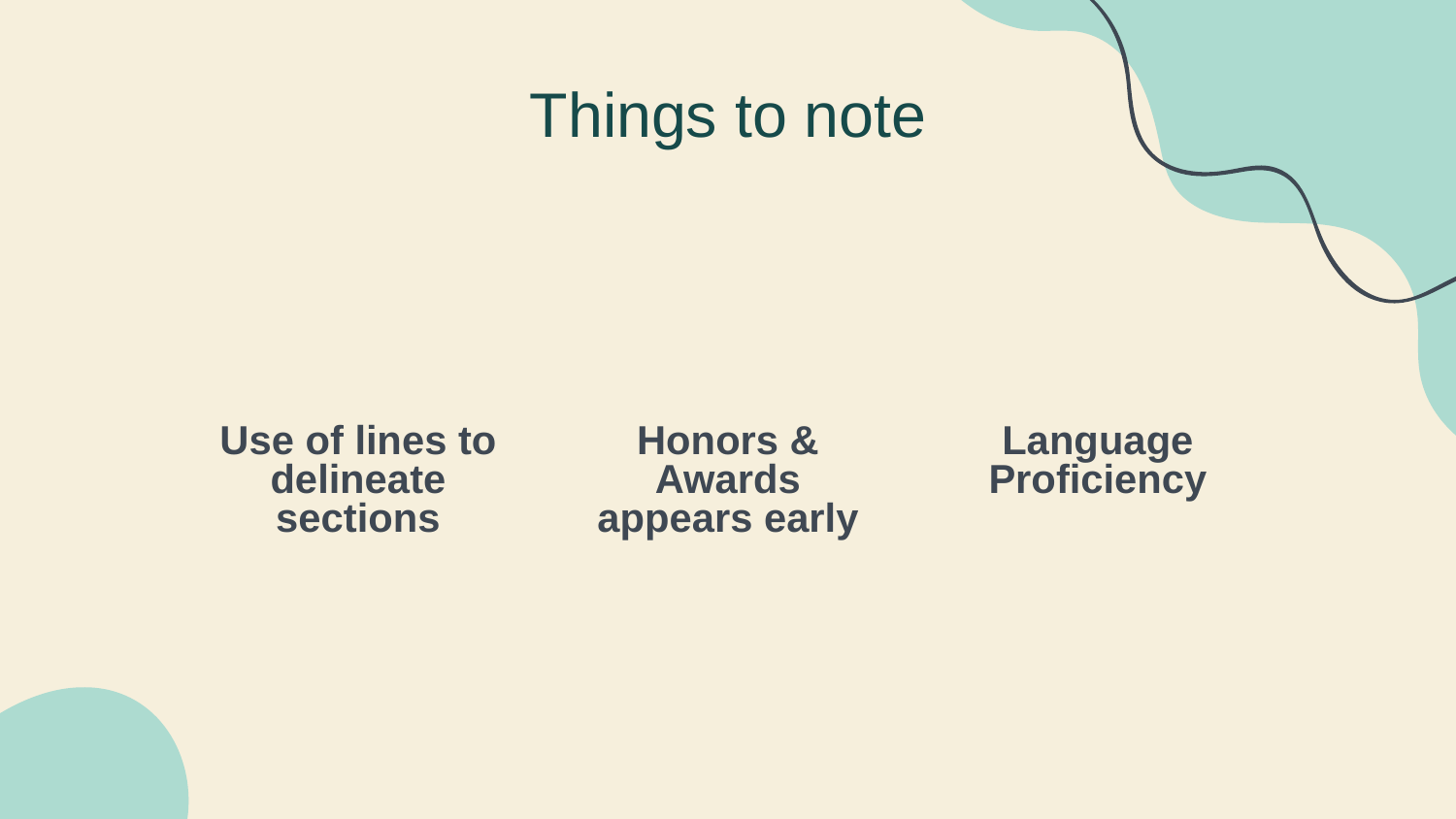

# Things to note
Use of lines to delineate sections
Honors & Awards appears early
Language Proficiency

## Slide 58
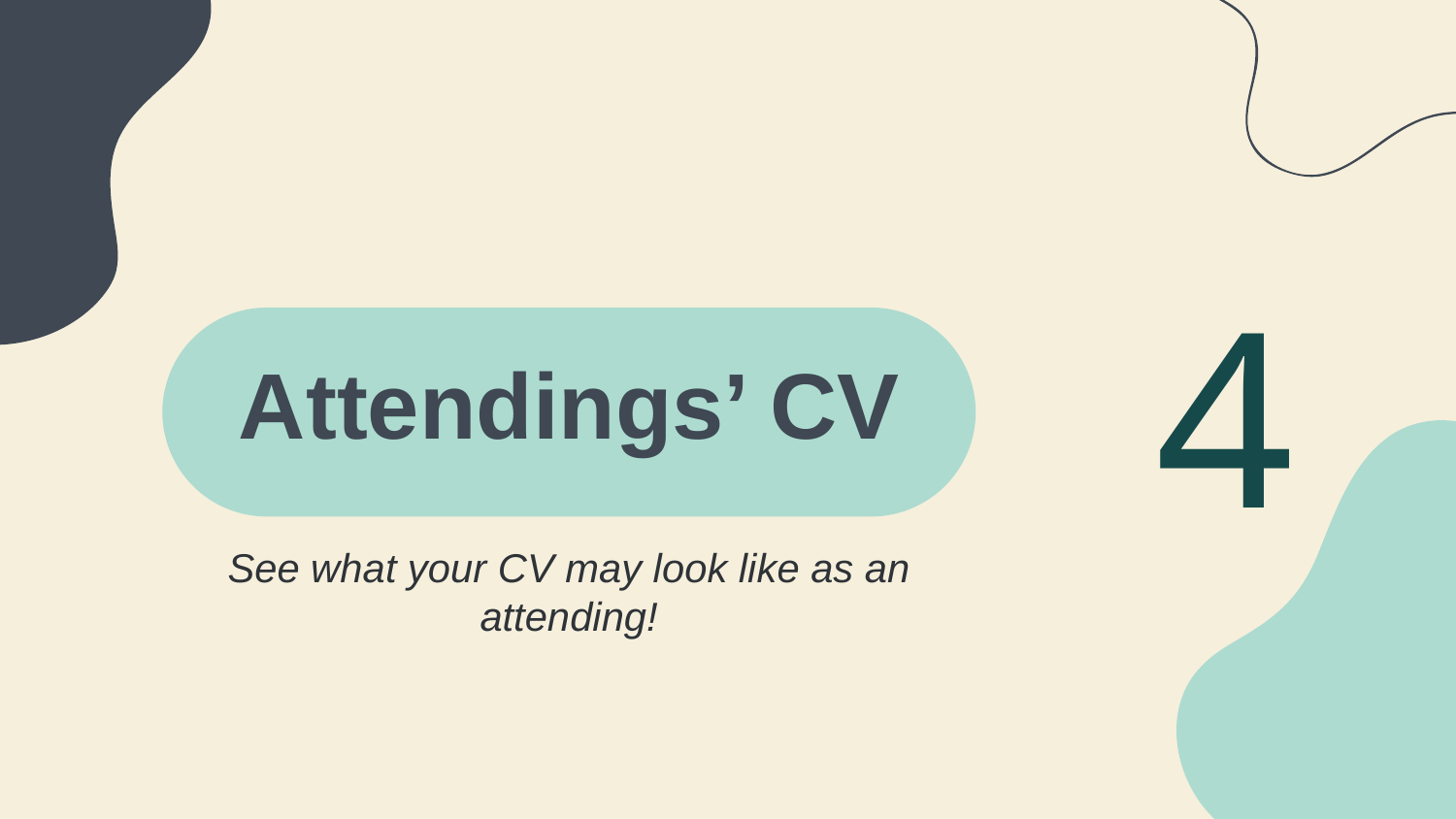

4
# Attendings’ CV
See what your CV may look like as an attending!

## Slide 59
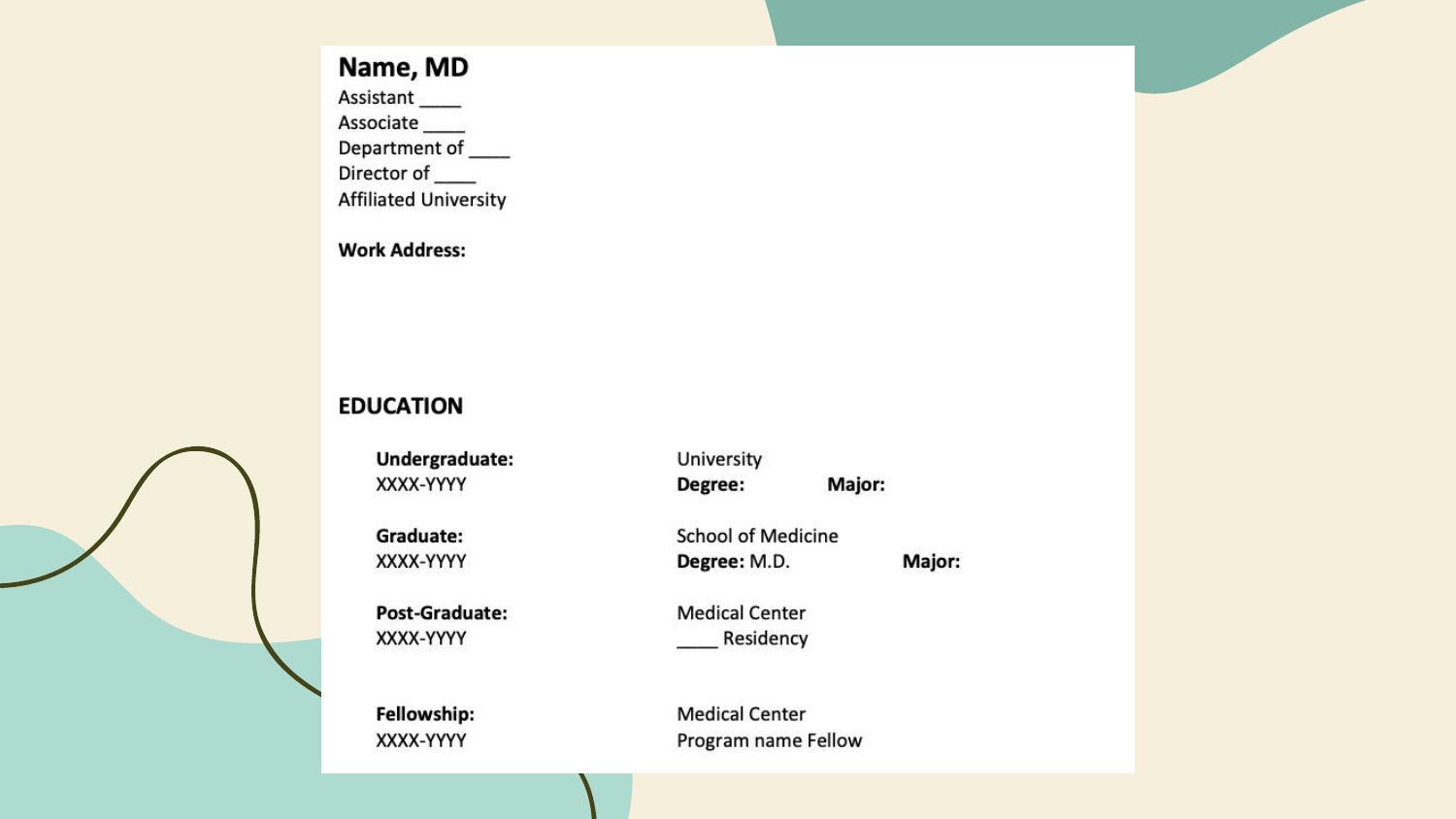

## Slide 60
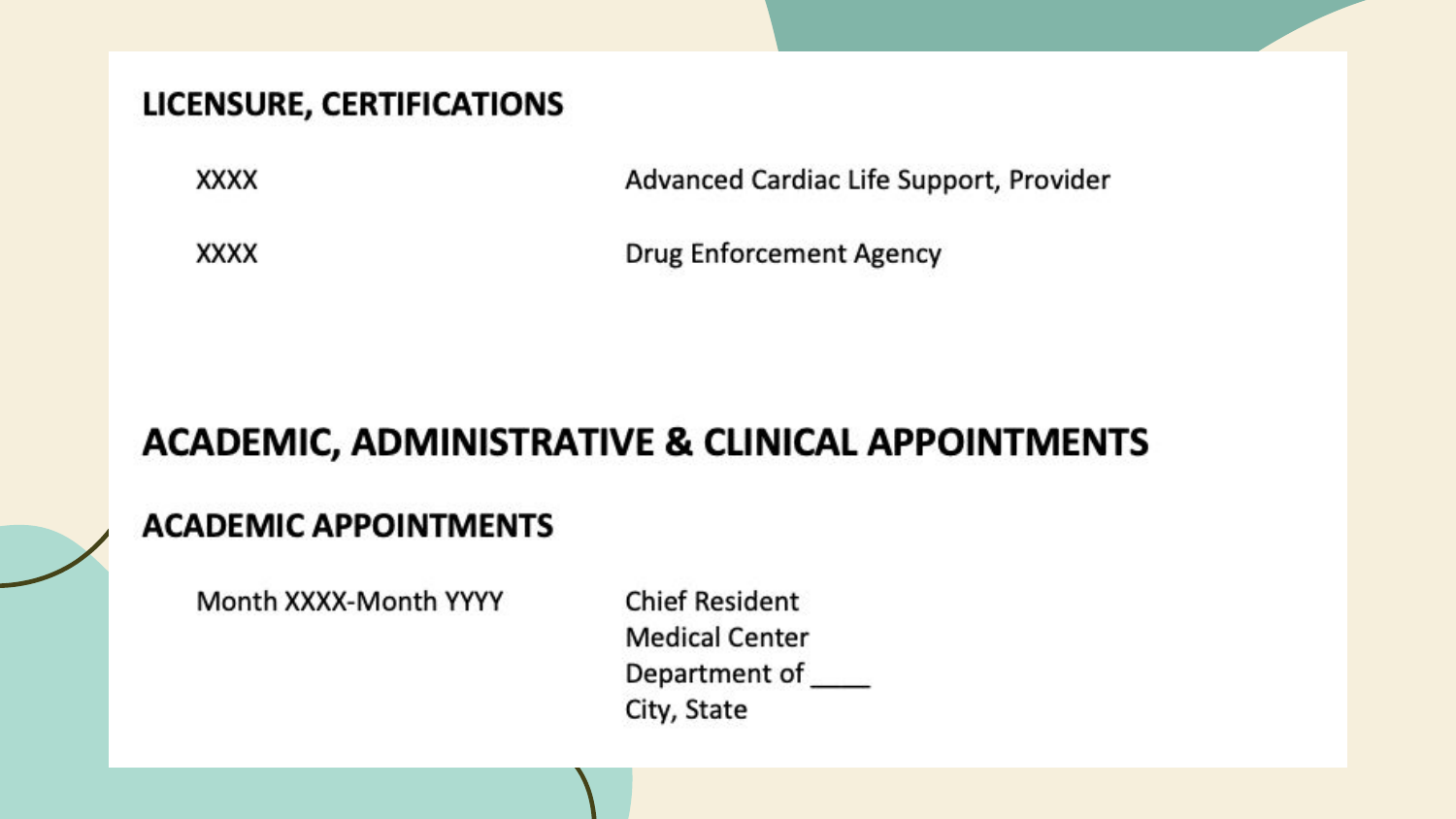

## Slide 61
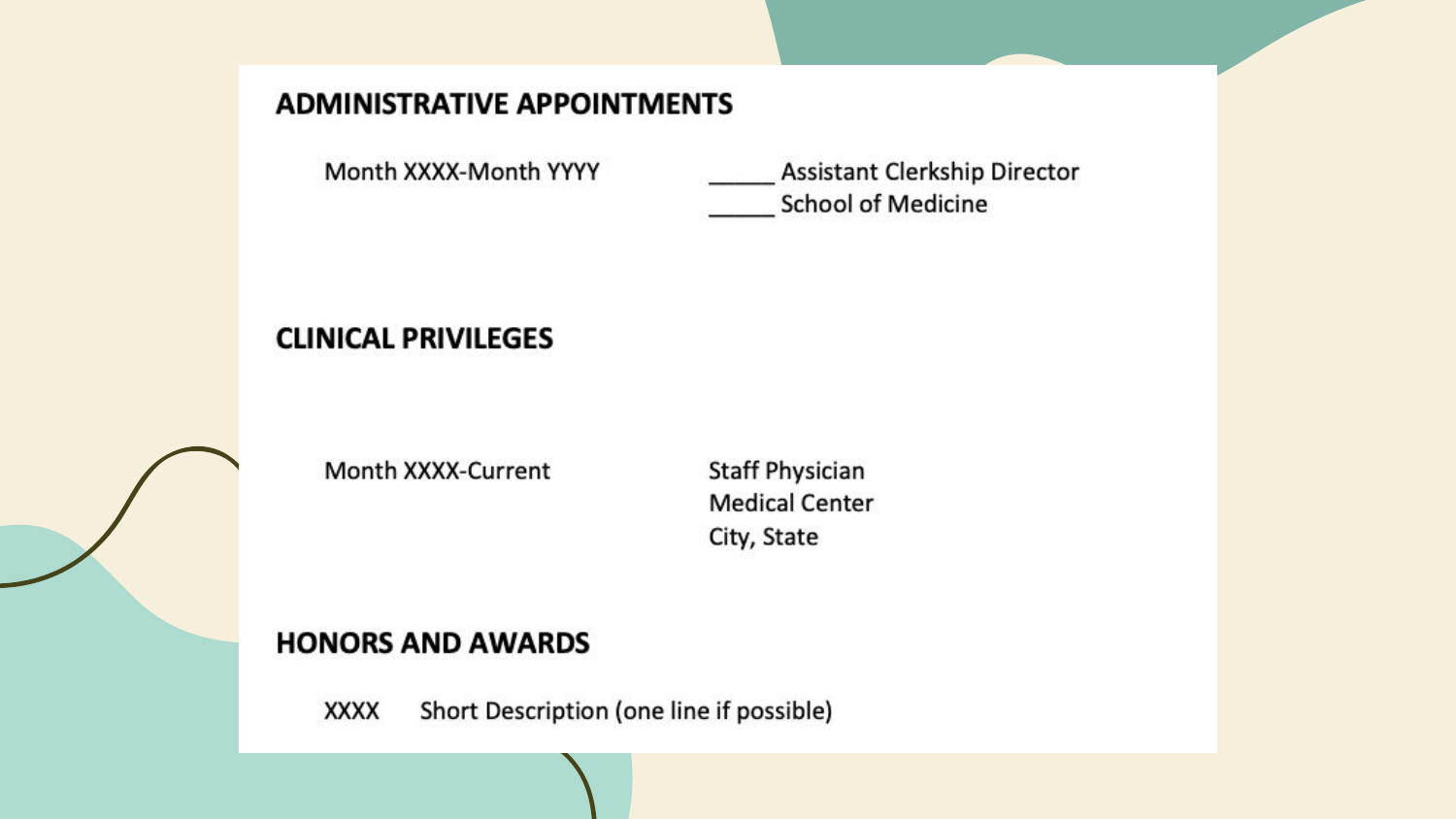

## Slide 62
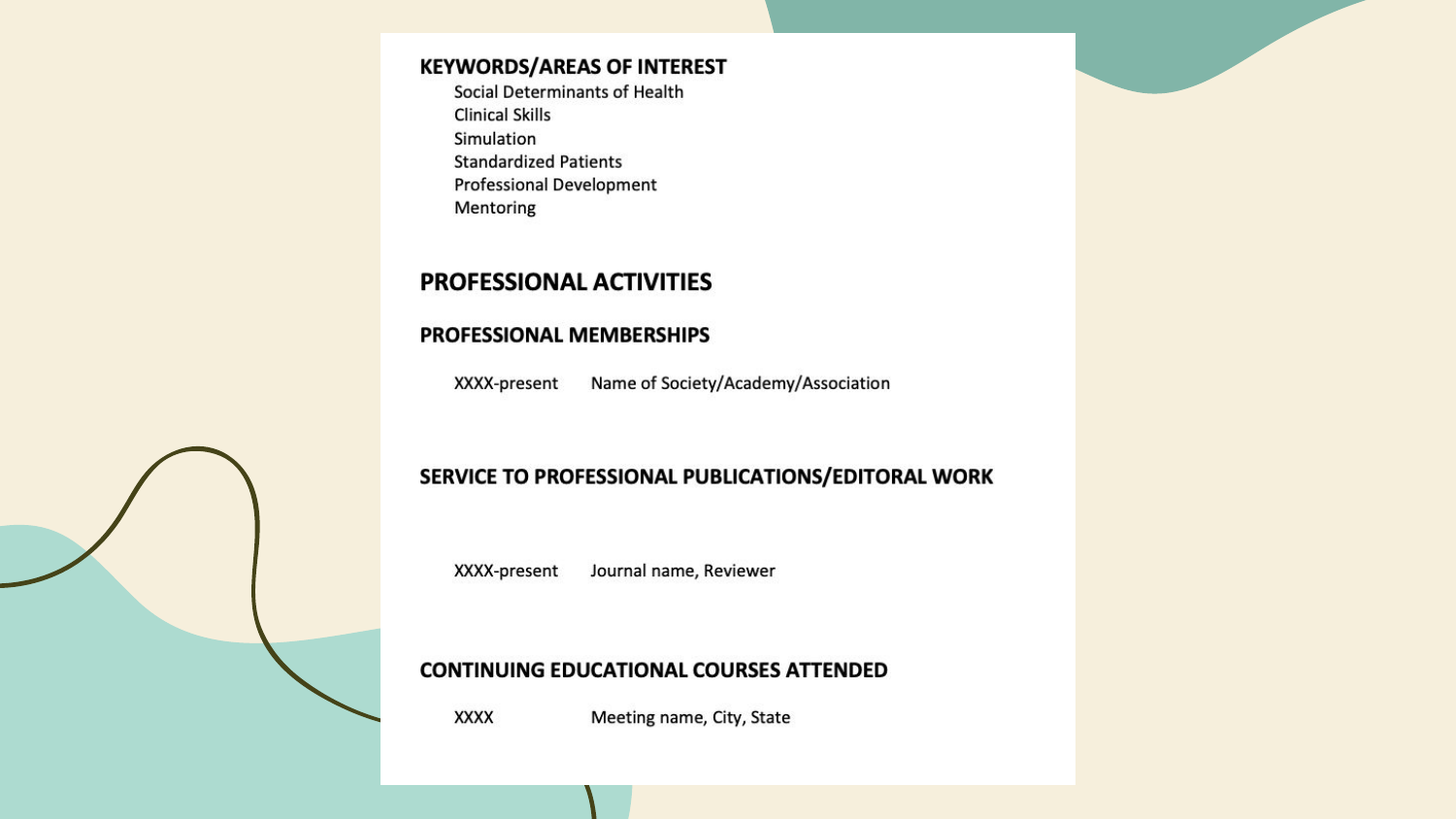

## Slide 63
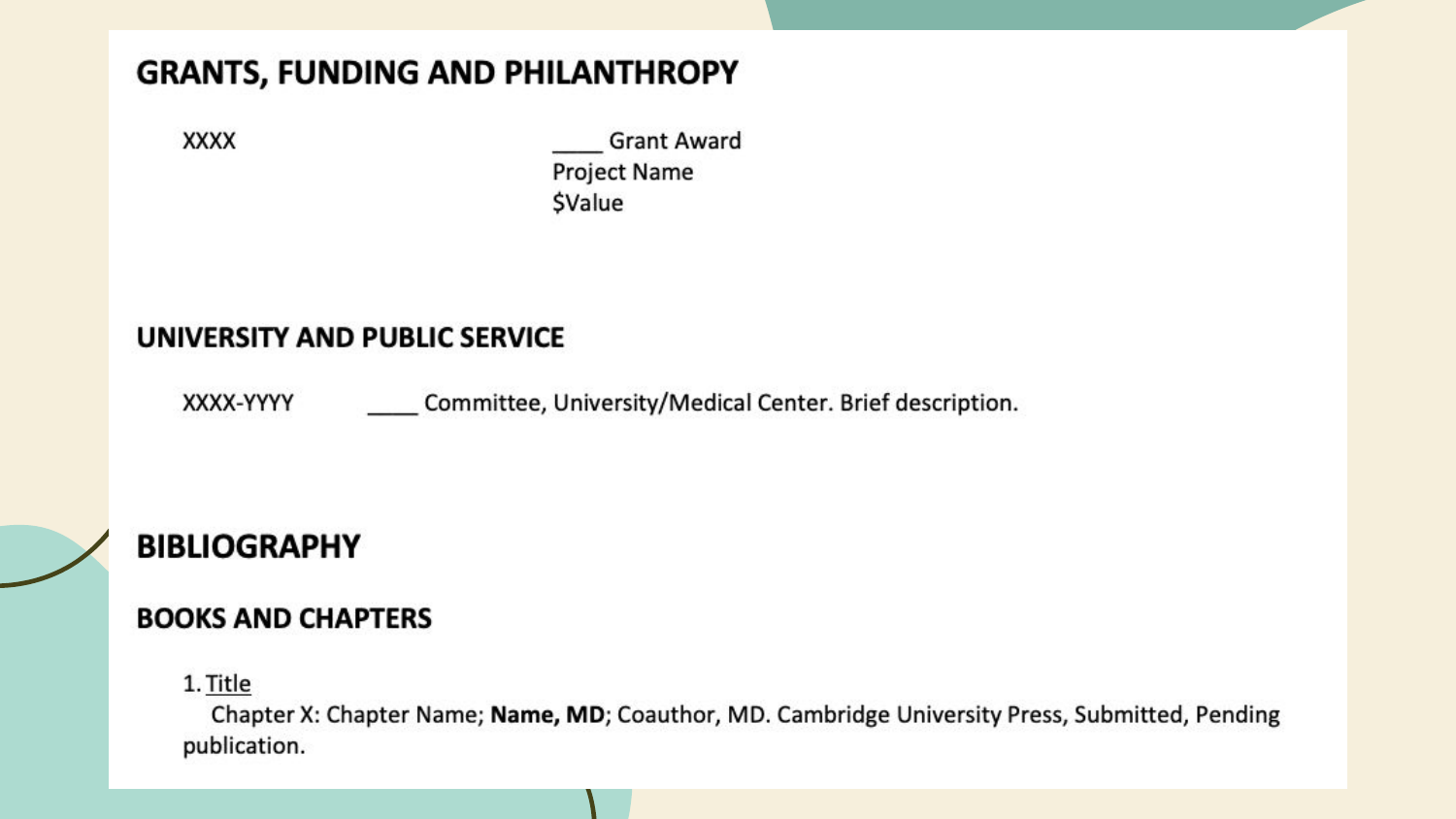

## Slide 64
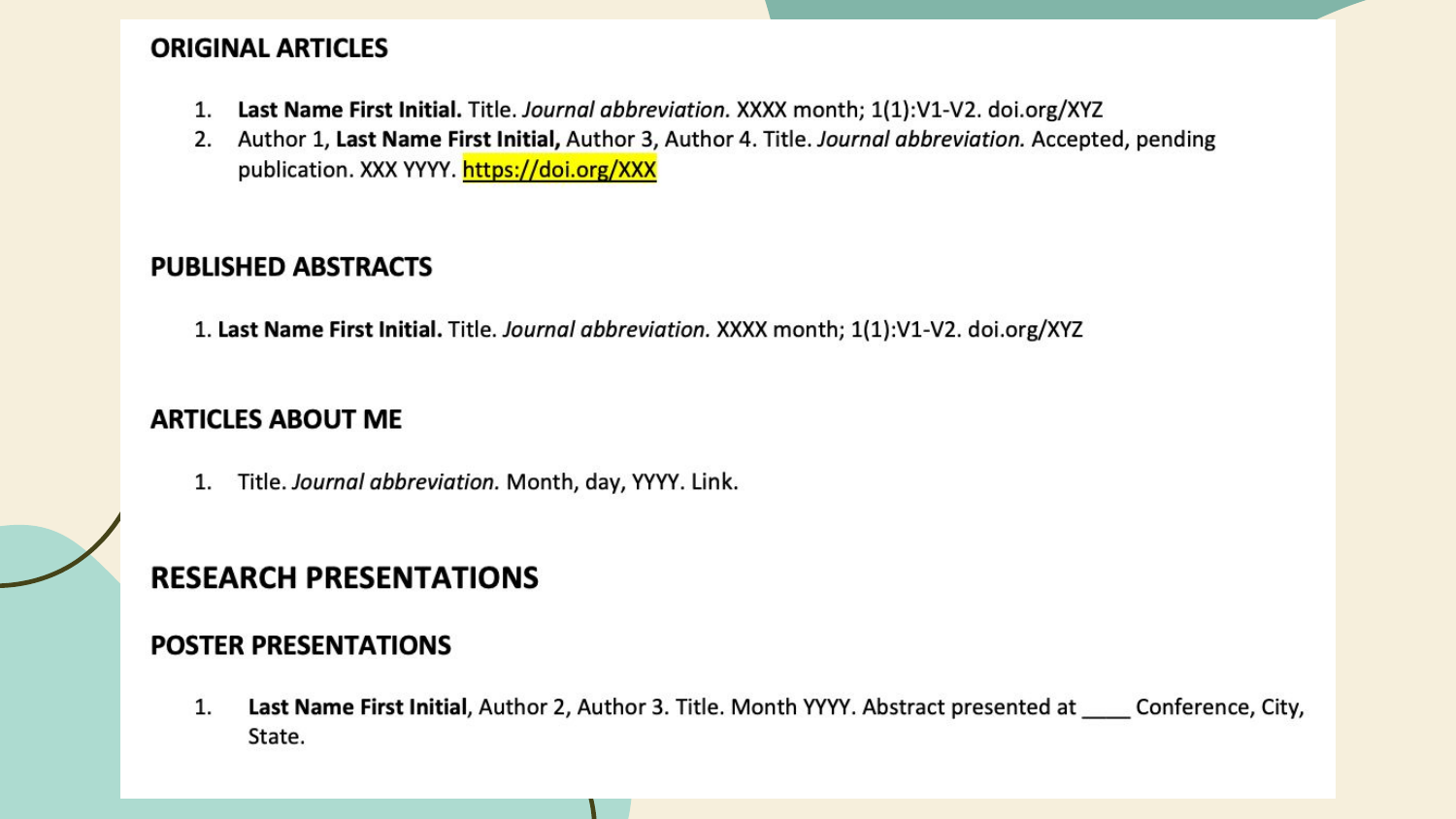

## Slide 65
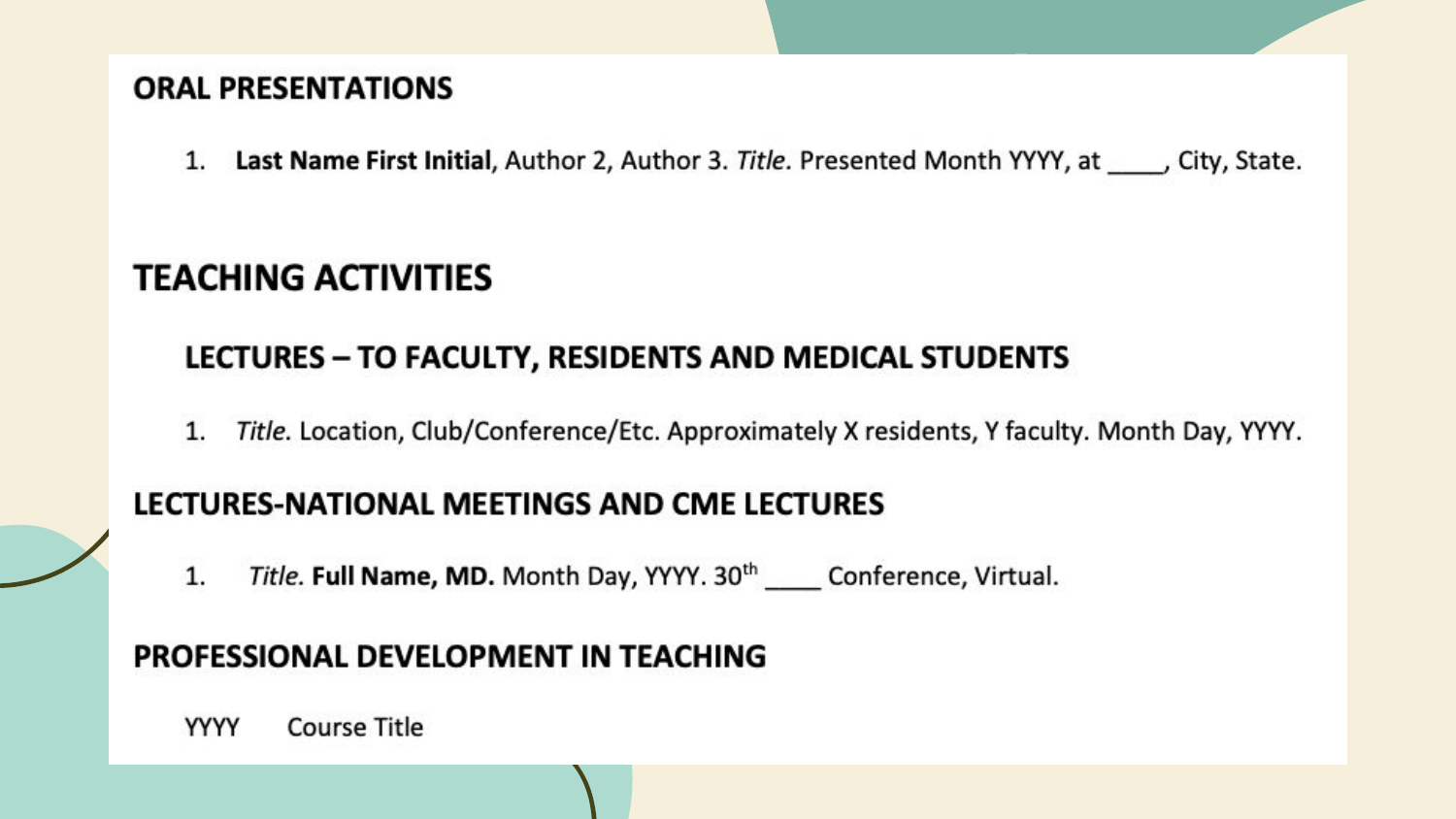

## Slide 66
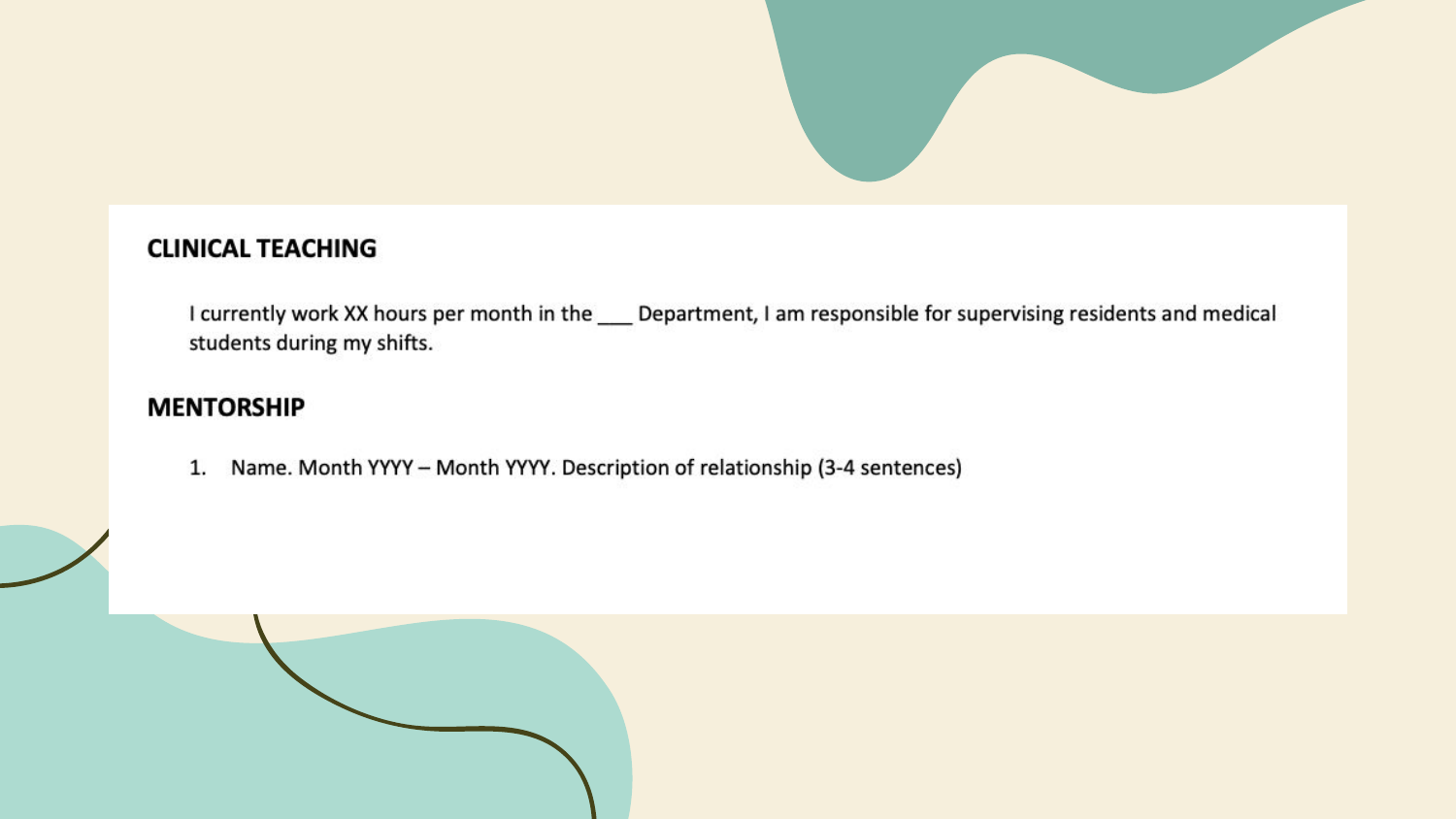

## Slide 67
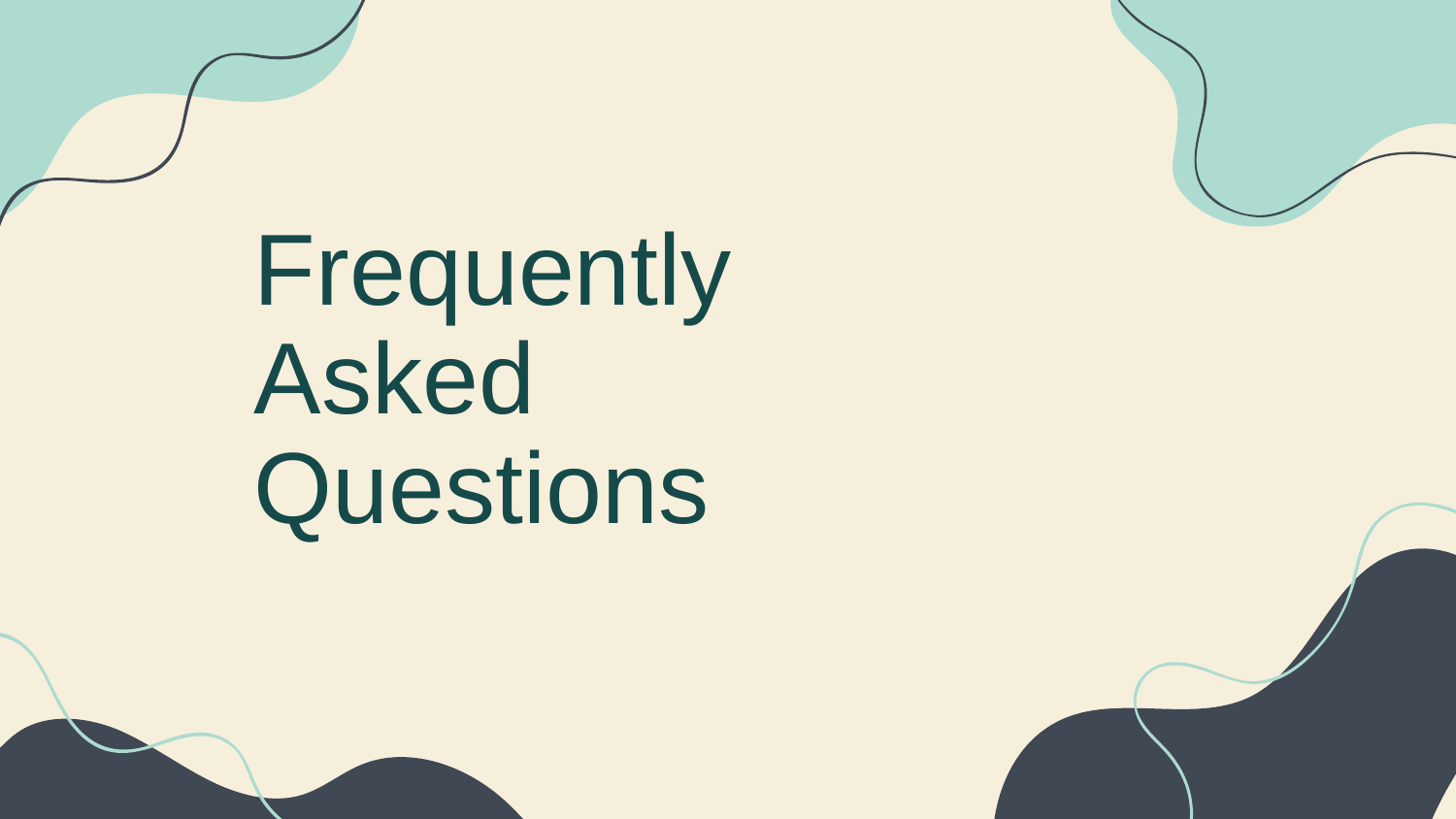

# Frequently Asked Questions

## Slide 68
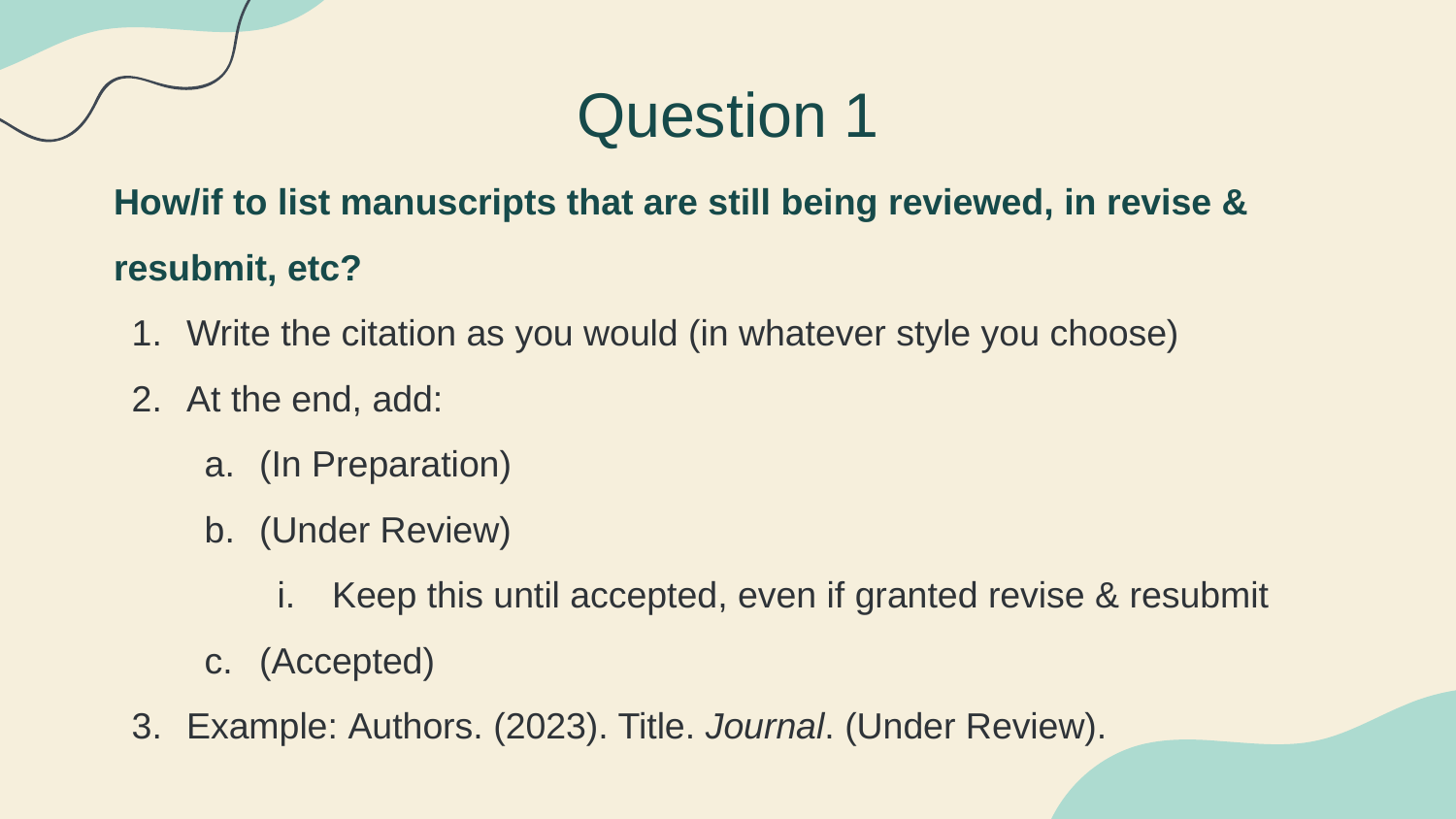

# Question 1
How/if to list manuscripts that are still being reviewed, in revise & resubmit, etc?
Write the citation as you would (in whatever style you choose)
At the end, add:
(In Preparation)
(Under Review)
Keep this until accepted, even if granted revise & resubmit
(Accepted)
Example: Authors. (2023). Title. Journal. (Under Review).

## Slide 69
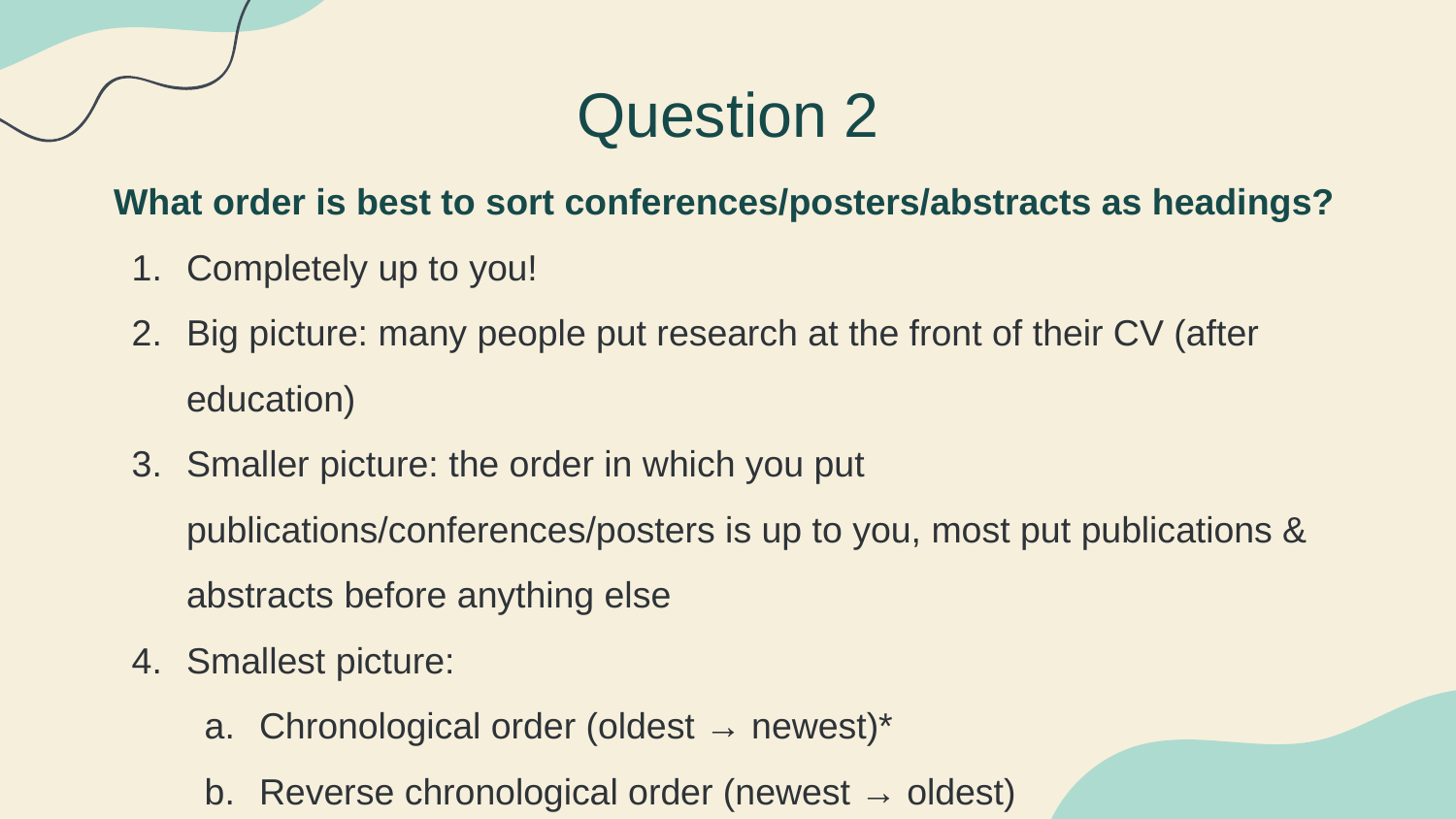

# Question 2
What order is best to sort conferences/posters/abstracts as headings?
Completely up to you!
Big picture: many people put research at the front of their CV (after education)
Smaller picture: the order in which you put publications/conferences/posters is up to you, most put publications & abstracts before anything else
Smallest picture:
Chronological order (oldest → newest)*
Reverse chronological order (newest → oldest)

## Slide 70
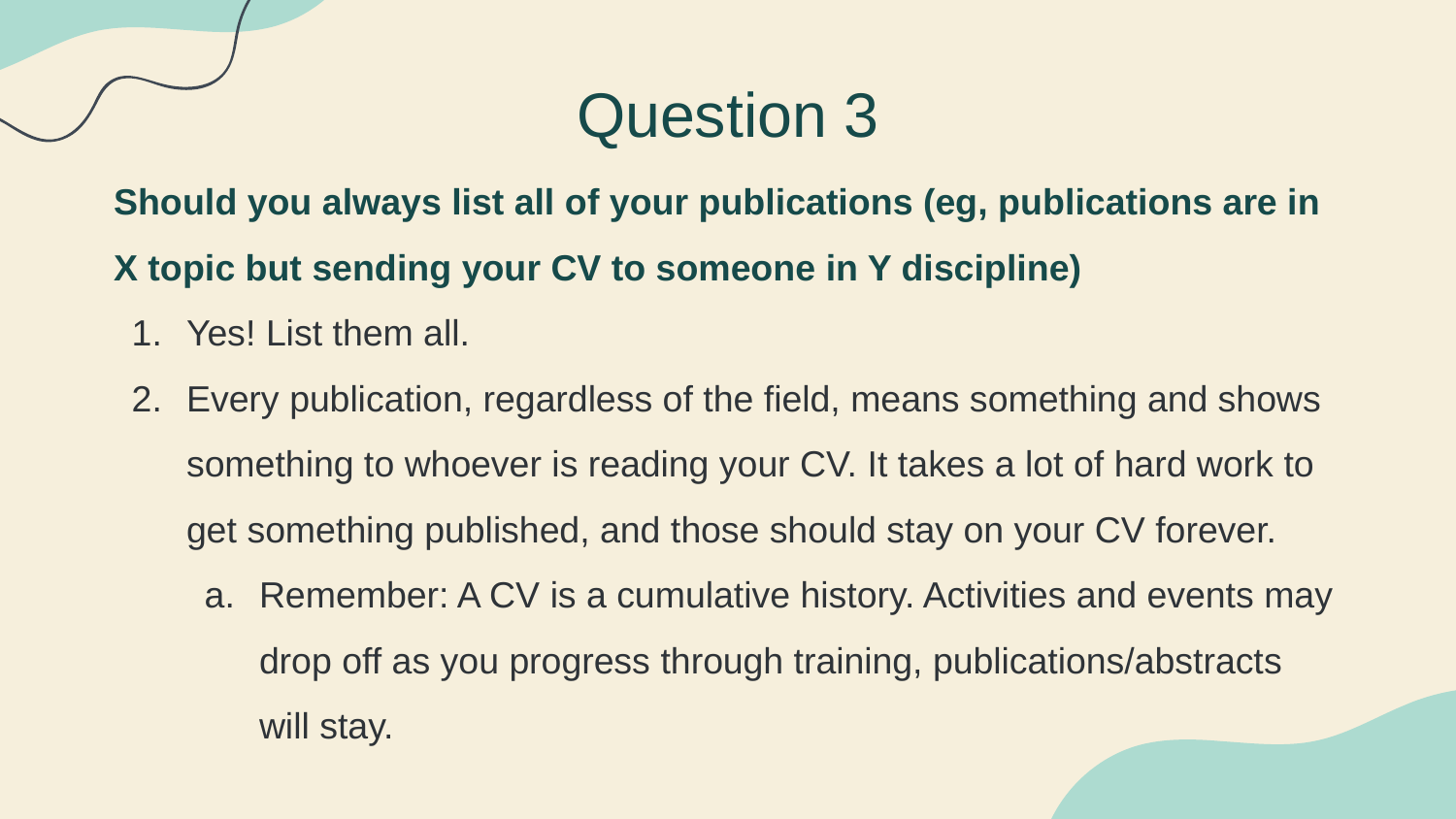

# Question 3
Should you always list all of your publications (eg, publications are in X topic but sending your CV to someone in Y discipline)
Yes! List them all.
Every publication, regardless of the field, means something and shows something to whoever is reading your CV. It takes a lot of hard work to get something published, and those should stay on your CV forever.
Remember: A CV is a cumulative history. Activities and events may drop off as you progress through training, publications/abstracts will stay.

## Slide 71
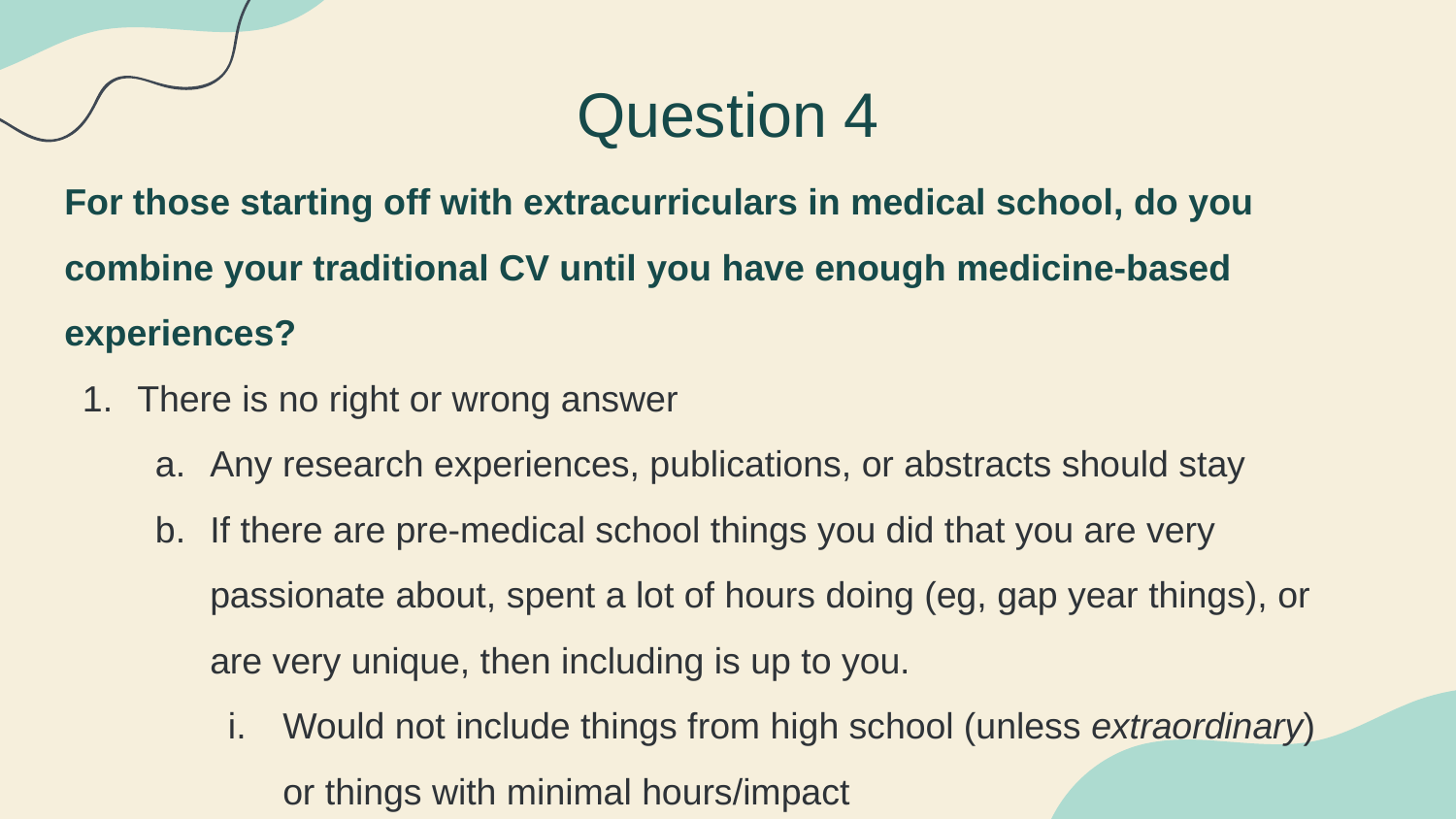

# Question 4
For those starting off with extracurriculars in medical school, do you combine your traditional CV until you have enough medicine-based experiences?
There is no right or wrong answer
Any research experiences, publications, or abstracts should stay
If there are pre-medical school things you did that you are very passionate about, spent a lot of hours doing (eg, gap year things), or are very unique, then including is up to you.
Would not include things from high school (unless extraordinary) or things with minimal hours/impact

## Slide 72
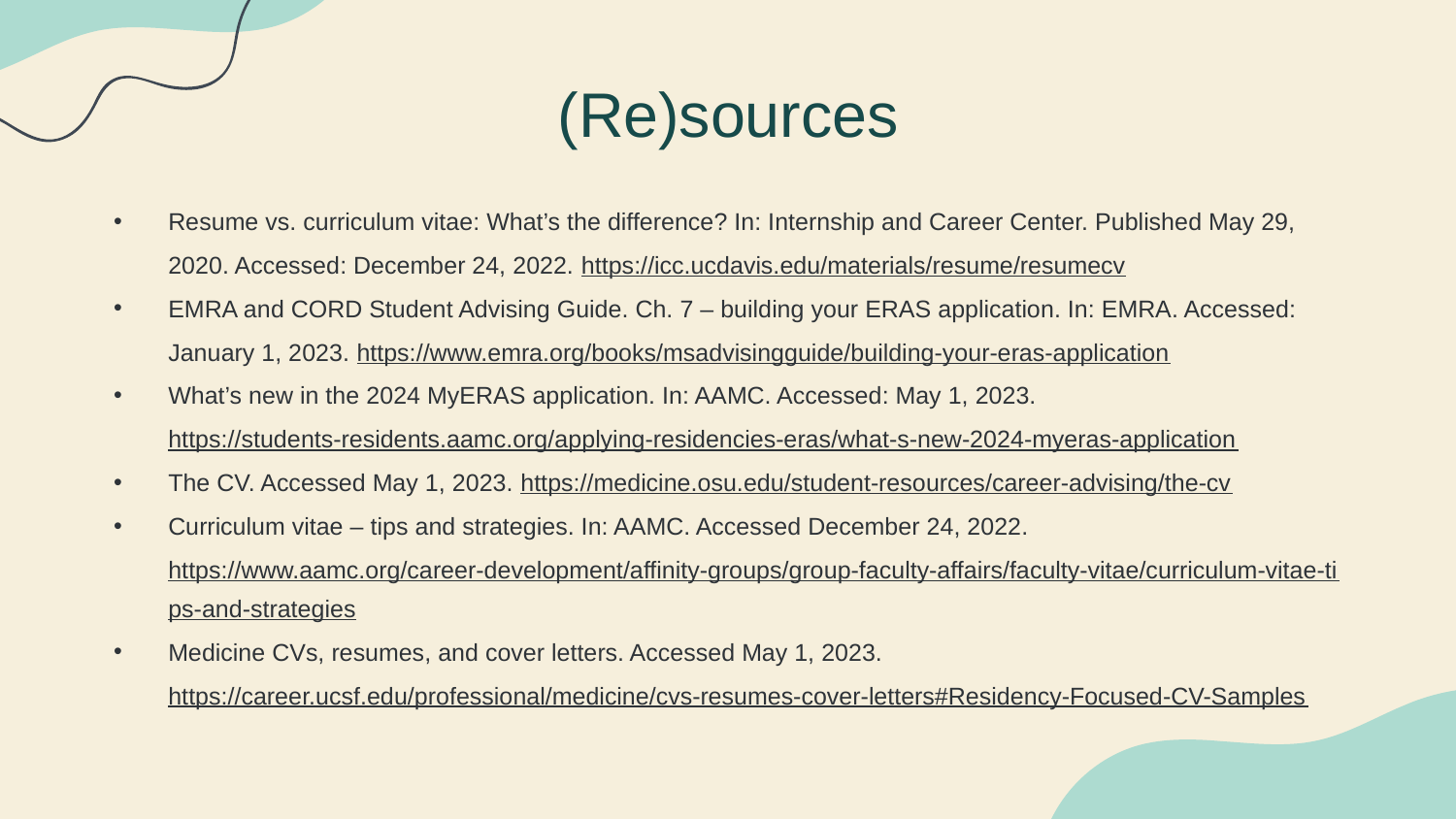

# (Re)sources
Resume vs. curriculum vitae: What’s the difference? In: Internship and Career Center. Published May 29, 2020. Accessed: December 24, 2022. https://icc.ucdavis.edu/materials/resume/resumecv
EMRA and CORD Student Advising Guide. Ch. 7 – building your ERAS application. In: EMRA. Accessed: January 1, 2023. https://www.emra.org/books/msadvisingguide/building-your-eras-application
What’s new in the 2024 MyERAS application. In: AAMC. Accessed: May 1, 2023. https://students-residents.aamc.org/applying-residencies-eras/what-s-new-2024-myeras-application
The CV. Accessed May 1, 2023. https://medicine.osu.edu/student-resources/career-advising/the-cv
Curriculum vitae – tips and strategies. In: AAMC. Accessed December 24, 2022. https://www.aamc.org/career-development/affinity-groups/group-faculty-affairs/faculty-vitae/curriculum-vitae-tips-and-strategies
Medicine CVs, resumes, and cover letters. Accessed May 1, 2023. https://career.ucsf.edu/professional/medicine/cvs-resumes-cover-letters#Residency-Focused-CV-Samples

## Slide 73
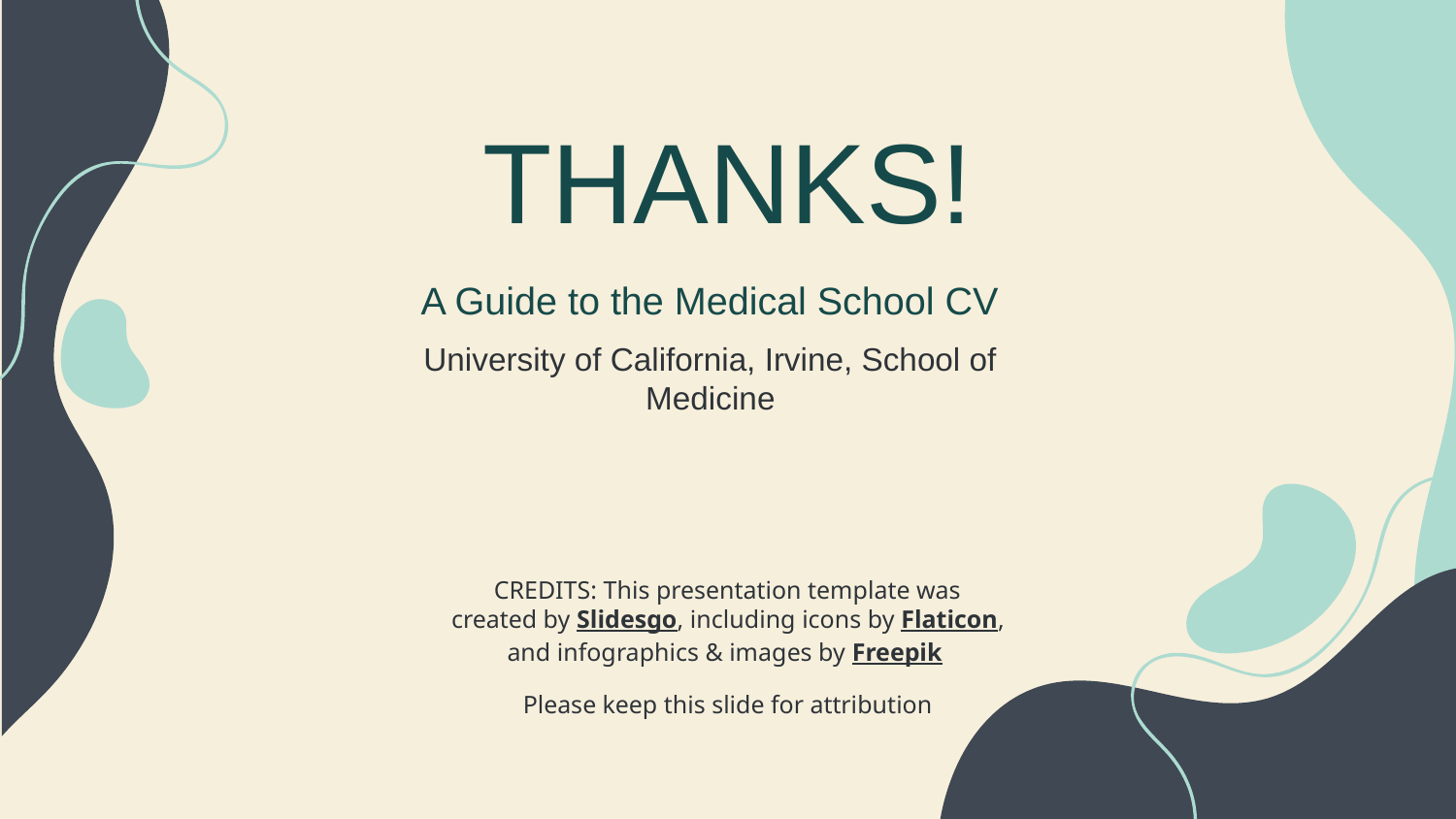

# THANKS!
A Guide to the Medical School CV
University of California, Irvine, School of Medicine
Please keep this slide for attribution
