# Supplementary material for: A Guide to the Medical School Curriculum Vitae
Source: J Educ Teach Emerg Med. 2024 Jan 31;9(1):L1–L20. doi: 10.21980/J8HH1S (PMC10854880; doi:10.21980/J8HH1S)
Supplement: Supplementary file 2 [file jetem-9-1-L1-supp2.docx]

***Name***

Email ∙ (c) XXX-XXX-XXXX ∙ Address

**Education**

University of California, Irvine M/Y – Present

School of Medicine (MD)

MS – Degree expected ___

UNDERGRAD M/Y – Present

Bachelor of Science, Biological Sciences

**Awards/Honors**

X Award (included $X stipend) Year

**Peer-Reviewed Publications**

- Authors (202X). Title. *Journal in PubMed format.* [DOI.](https://doi.org/10.1080/23794925.2021.1923090)

**Abstracts**

- **Authors**. (2020). *Title.*Poster presented at the annual meeting for ___, Virtual Meeting, Date. Program No. 445.068. 2020 Abstract Viewer/Itinerary.
- **Authors.** (2022) *Title*. Presentation given as part of the UCISOM Medical Student Research Symposium, Virtual Meeting, November 4^th^, 2022.

**Research Experience**

**Collaborator,** Hospital M/Y – Present

Faculty Mentor – XYZ

- Conducted research in collaboration with the ___, specifically looking at ___.

**Lectures/Presentations/Panels**

1. *Title.* Presentation. Location, Event. XX __ students. Month day, year.
2. *Title.* Lecture. XX __ students. Month day, year.
3. *Title.* Panel. XX ____ attendees. Month day, year.

**Other Publications**

- **Name**. (year). Title. [Link.](https://www.emergencymed.uci.edu/features/spotlight-osborn.asp)

**Leadership Experience**

**Program + Title,** UC Irvine School of Medicine M/Y – Present

Faculty Mentor –

- Oversaw a team of over X ____ while working with our ___ to complete administrative tasks (examples), tasks to ___, and tasks to advance the program (examples).

**School of Medicine Professional Activities**

**___ Interest Group ___** M/Y – Present

Faculty mentor –

- Organized and facilitated ____ for medical students (examples).

**Class Representative**

Class – Faculty mentor – M/Y – Present

- Served as a class representative for UCISOM’s ___ to act as a liaison between the course directors and my fellow classmates by collecting data and information from my classmates (ex: Qualtrics surveys) about their experience to facilitate improvements.

**Selected Volunteer/Community Engagement**

**Title** M/Y – Present

Where?

- What

**Service Learning Project** M/Y – Present

Faculty Mentors – Dr. Charles Vega MD

- Completed a project during MS1 and MS2 years to benefit the greater Orange County area through development of a ___ to assist in ___.

**Work Experience**

**____,** UC Irvine School of Medicine M/Y – Present

Faculty Mentor –

- Served as a ___ during academic year 2022-2023 to ___.

**Professional Memberships**

Member, XYZ M/Y – Present

**Hobbies/Interests**

**Mentees**

1. Name, year-present. I mentored ____ + some explanation of what you did/where they are now.
