# Supplementary material for: A Guide to the Medical School Curriculum Vitae
Source: J Educ Teach Emerg Med. 2024 Jan 31;9(1):L1–L20. doi: 10.21980/J8HH1S (PMC10854880; doi:10.21980/J8HH1S)
Supplement: Supplementary file 3 [file jetem-9-1-L1-supp3.docx]

**Name**

Address • cell • email

**Education**

Undergrad MM/YYYY – MM/YYYY

- Degree

University of California, Irvine - Irvine, CA MM/YYYY - Current

- M.D. Candidate, expected ____

**Research & Publications**

___ Lab (Student Researcher) - Irvine, CA MM/YYYY – MM/YYYY

- Conducted research in _____
- Studied ____

Authors. Title. Journal. YYMM citation info. doi: PMID: XYZ

**ACCEPTED Abstracts**

Authors. Title. Congress of ___Annual Meeting. City, State. X-Y Month. Year.

**ACCepted Poster presentations**

Authors. Title. ____ Meeting. City, State. X-Y Month. Year.

**Work Experience­s­**

____ Tutor (Student Teacher) - Irvine, CA MM/YYYY - Current

- Tutored 1^st^ year medical students in ____

**Volunteer Experience­s­**

Program (Mentor) - Irvine, CA MM/YYYY - Current

- Served as a medical student mentor for ____

**clinical Activities**

UCI ___ Clinic (Medical Student Volunteer) - Irvine, CA MM/YYYY - Current

- Practiced history taking and other clinical skills while volunteering at a free clinic
- Presented patients to the attending physician and formulated an assessment & treatment plan along with a MS3 or MS4

**Leadership­**

___ Interest Group (Title) - Irvine, CA MM/YYYY - Current

- Started an interest group to ____

**Miscellaneous**

UCI SOM Willed Body Celebration - Irvine, CA MM/YYYY

- Attended and assisted with the UCI SOM Willed Body Celebration honoring the families and loved ones of generous body donors to the medical anatomy program.

**Mentorship**

1. Name Month/Year to present. I met ___. I continue to help ___.

**Honors/Awards**

- ____ Scholarship (X amount) MM/YYYY
