# Supplementary material for: A Guide to the Medical School Curriculum Vitae
Source: J Educ Teach Emerg Med. 2024 Jan 31;9(1):L1–L20. doi: 10.21980/J8HH1S (PMC10854880; doi:10.21980/J8HH1S)
Supplement: Supplementary file 4 [file jetem-9-1-L1-supp4.docx]

Name

[Email](mailto:yangcd1@hs.uci.edu)

**Education University of California, Irvine School of Medicine** YYYY-present Doctor of Medicine (M.D.), in progress

**University of California, XYZ** YYYY-YYYY

Bachelor of Science (B.S.), X

**Honors and Awards**

X Scholarship YYYY-YYYY

**Societies and Memberships**

American College of ___ YYYY-present

**Research Interests**

1. X
2. Y
3. Z

**Research Support** X Award YYYY-present University of California, Irvine School of Medicine

PI: ____

Grant Info

**Research Experience**

**Publications and Journal Proceedings**

**Conference Proceedings**

**Oral Presentations**

**University of California, Irvine School of Medicine** YYYY-present Medical Student Researcher, Department of _____ Irvine, CA Advisor:

- Validating a ___
- Characterizing the ___

1. Authors. Title (hyperlinked). *Journal*. YYYY MO D.
2. Authors. Title (hyperlinked) *X Symposia: XYZ*. City, State, Country. YYYY MO D. [Poster]
3. **Authors.** Title. *UCI School of Medicine Medical Student Research Symposium YYYY*. Irvine, CA, United States. YYYY MO DD.
