# Supplementary material for: A Guide to the Medical School Curriculum Vitae
Source: J Educ Teach Emerg Med. 2024 Jan 31;9(1):L1–L20. doi: 10.21980/J8HH1S (PMC10854880; doi:10.21980/J8HH1S)
Supplement: Supplementary file 5 [file jetem-9-1-L1-supp5.docx]

**Name**

Irvine, CA | Cell | [Email](file:///Users/Konnor/Desktop/Jaschlos@hs.uci.edu)

***Education***

**University of California, Irvine** MMM YYYY – MMM YYYY

Doctor of Medicine

**Undergrad** MMM YYYY – MMM YYYY Bachelor of Science, Major in ___

***Relevant Experience***

**[Redacted] Medical Student Board Rep**, Association Name MMM YYYY – MMM YYYY

***Research Experience***

**Researcher**, UCI ____ MMM YYYY – Present

- Ran validation studies comparing ___
- Fluent in ___

**Undergraduate Research Assistant**, XXXX MMM YYYY – MMM YYYY

***Work Experience***

**Senior XYZ**, ____ MMM YYYY – MMM YYYY

- Applied X principles to improve ___.
- Educated ___
- Discussed ___.
- Determined ____

***Peer-Reviewed Publications***

2022 3 Authors, et al. Title. *Journal.* [DOI.](https://doi.org/10.3389/fneur.2022.1026609)

2019 3 authors, et al. Title. *Journal*. [DOI.](https://doi.org/10.1007/s11060-019-03132-z)

***Abstracts and Posters***

2022 3 authors, et al. Title. *Abstract presented at ____. Month, year.*

2019 3 authors, et al. Title. Abstract published *Journal*. [DOI.](https://doi.org/10.1093/neuonc/noz175.717)
