# Supplementary material for: A Guide to the Medical School Curriculum Vitae
Source: J Educ Teach Emerg Med. 2024 Jan 31;9(1):L1–L20. doi: 10.21980/J8HH1S (PMC10854880; doi:10.21980/J8HH1S)
Supplement: Supplementary file 6 [file jetem-9-1-L1-supp6.docx]

NAME

REDACTED C.V. – Page 1 of [8](#page8)

Education

YYYY-YYYY **University of California, Irvine, MD**.

YYYY–YYYY **XYZ, degree**.

Majors: XYZ

Research Experience

| Research | **Student Researcher**, *UCI XYZ*. | |
| --- | --- | --- |
|  | MO YYYY – Present | Irvine, CA |
|  | XYZ, PhD |  |
|  | - I am currently working on ___. | |
|  | - I worked on ___. | |
|  |  | |
| Clinical | **Student Researcher**, *Department of [REDACTED], UC Irvine*. | |
| Research | MO YYYY – Present | ½ Irvine, CA |
|  | XYZ, MD |  |
|  | XYZ, MD, PhD |  |
|  | - I worked on various clinical projects and took a lead in ____. | |
|  |  | |

Redacted C.V. – Page 2 of [8](#page8)

Work Experience

Teaching **X Teacher Assistant and Tutor**, *[redacted]*.

MO YYYY – MO YYYY Irvine, CA

- - Instructed/Tutored ____

School of Medicine Professional Activities

Interest Group **XYZ Interest Group Founder** *University of California Irvine School of Medicine*.

MO YYYY – present Irvine, CA

Faculty mentor –

Admission **School of Medicine Admissions Committee**, *University of California Irvine School of Medicine*.

MO YYYY – present Irvine, CA

- - Worked alongside the committee to interview applicants, holistically review applications, and provide recommendations for admission or rejection to the medical school.

Redacted C.V. – Page 3 of [8](#page8)

Honors and Awards

MO 2022 2022 XYZ

|  |
| --- |

Involvements and Leadership

| YYYY –YYYY | Position | *XYZ Interest Group* | |
| --- | --- | --- | --- |
| YYYY – YYYY | Mentor |  | *Guiding Hands @ UCI SOM* |

Redacted C.V. – Page 5 of [8](#page8)

Publications

Peer Reviewed

MO YYYY Authors. Title. Journal. Date. PMID: PMCID: doi:

Abstracts

MO YYYY Authors; Title. Journal.

Other Publications and Digital Content

Redacted. – Page 6 of [8](#page8)

Conference Presentations

Oral Presentations

May 2022 Authors. Title. Oral presentation at ___ Annual Meeting, City (Month day, year). City, State, Country.

Poster Presentations

*Co-Authorship

Oct 2022 **Author** (Presenter), Title. Poster Presentation at ____ Meeting. Month, Day, Year, Irvine, CA, USA.

Clinical Activities

**Medical Student**, *Patient And Community Engagement (PACE) Clerkship*

MO YYYY – MO YYYY Irvine, CA

Faculty Mentor XYZ, MD

- - Meet with patients in the clinic and obtain a history and physical exam. Present patient history and physical exams to the attending physician.

Community Service

Clinical **Medical Student Volunteer**, *___ at UCI Orange County*, Lestonnac Free Clinic.

MO YYYY – present Orange, CA

- Trained to ____.

Invited Presentations
