# Supplementary material for: A Guide to the Medical School Curriculum Vitae
Source: J Educ Teach Emerg Med. 2024 Jan 31;9(1):L1–L20. doi: 10.21980/J8HH1S (PMC10854880; doi:10.21980/J8HH1S)
Supplement: Supplementary file 7 [file jetem-9-1-L1-supp7.docx]

**NAME**

Address

T: E:

**Education**

- University of California, Irvine School of Medicine: MO YYYY-present

Doctor of Medicine – Expected YYYY

- University of California, ____: MO YYYY – MO YYYY

Bachelors of Science, _____

**Posters**

- Authors. *Title.* Asian Pacific American Medical Student Association National Conference. MO D YYYY.

**School of Medicine Professional Activities**

- **Ultrasound Interest Group:** MO YYYY – MO YYYY
  - ***Role***
  - Led the ____.
- **Associated Medical Student Government (AMSG):** MO YYYY-present
  - ***Role***
  - Coordinate with UCISOM Administration ___.
  - Responsible for ____.

**Professional Experience**

- **XYZ Hospital Emergency Department:** MO YYYY – MO YYYY
  - ***Chief Scribe***
  - Led, hired, and trained 40 other scribes and acted as a liaison between the physicians and ScribeAmerica to facilitate efficiency in the emergency department. Helped in the organization of continuing medical education events as well as transition to Epic EMR. Served as a medical scribe for two years prior to becoming Chief Scribe.
