# Supplementary material for: A Guide to the Medical School Curriculum Vitae
Source: J Educ Teach Emerg Med. 2024 Jan 31;9(1):L1–L20. doi: 10.21980/J8HH1S (PMC10854880; doi:10.21980/J8HH1S)
Supplement: Supplementary file 8 [file jetem-9-1-L1-supp8.docx]

Name

*Address | Cell |* [*Email*](mailto:aavina1@uci.edu)


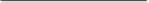


# EDUCATION

Masters in X Candidate MO YYYY – MO YYYY University of California, Irvine

M.D. Candidate. MO YYYY – MO YYYY

University of California, Irvine School of Medicine

Post-Baccalaureate Program MO YYYY – MO YYYY

University of ____

Bachelors of Science in Y MO YYYY – MO YYYY

University of ____


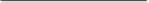


# HONORS & AWARDS

X Award MO YYYY


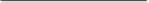


# POSTER PRESENTATIONS

1. Authors. **Title.** ____ Association Annual Meeting and Expo. Month, YYYY.

# ORAL PRESENTATIONS

1. Authors. **Title**. Conference + Location. Month, YYYY.


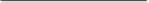


# RESEARCH EXPERIENCE

Title

Research Assistant Month, YYYY - Present

Supervisors:

- - Intercollaborative study that examines the ____. We hypothesize that the implementation of an…

Undergraduate Research Assistant MO YYYY- MO YYYY X Institute, Irvine CA

Supervisors:

- - Studied the ____.


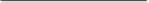


# LEADERSHIP EXPERIENCE

Task Force on ____ Medical Education MO YYYY - Present Committee Member

- - …

X Interest Group MO YYYY - Present Co-X of Y

- - Responsible for organizing and executing workshops on behalf of our ____.


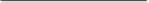


# SELECTED VOLUNTEER/COMMUNITY ENGAGEMENT

Guiding Hands MO YYYY - Present

Mentor

- - Longitudinal mentorship program for pre-medical students in Orange County, especially those at schools that lack in pre-medical guidance, resources, and support. We aim to provide long-term mentor relationships, advice with course selection and extracurriculars, insight into the medical school application process, and a familial support network for disadvantaged students.


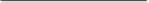


# PROFESSIONAL MEMBERSHIPS

American College of X YYYY

Language Proficiency: English
