# Supplementary material for: A Guide to the Medical School Curriculum Vitae
Source: J Educ Teach Emerg Med. 2024 Jan 31;9(1):L1–L20. doi: 10.21980/J8HH1S (PMC10854880; doi:10.21980/J8HH1S)
Supplement: Supplementary file 9 [file jetem-9-1-L1-supp9.docx]

**Name, MD**

Assistant ____

Associate ____

Department of ____

Director of ____

Affiliated University

**Work Address:**

**EDUCATION**

**Undergraduate:** University

XXXX-YYYY **Degree:** **Major:**

**Graduate:** School of Medicine

XXXX-YYYY **Degree:** M.D. **Major:**

**Post-Graduate:** Medical Center

XXXX-YYYY ­­­____ Residency

**Fellowship:** Medical Center

XXXX-YYYY Program name Fellow

**LICENSURE, CERTIFICATIONS**

XXXX Advanced Cardiac Life Support, Provider

XXXX Drug Enforcement Agency

**ACADEMIC, ADMINISTRATIVE & CLINICAL APPOINTMENTS**

**ACADEMIC APPOINTMENTS**

Month XXXX-Month YYYY Chief Resident

Medical Center

Department of ____

City, State

**ADMINISTRATIVE APPOINTMENTS**

Month XXXX-Month YYYY _____ Assistant Clerkship Director

_____ School of Medicine

**CLINICAL PRIVILEGES**

Month XXXX-Current Staff Physician

Medical Center

City, State

**HONORS AND AWARDS**

XXXX Short Description (one line if possible)

**KEYWORDS/AREAS OF INTEREST**

Social Determinants of Health

Clinical Skills

Simulation

Standardized Patients

Professional Development

Mentoring

**PROFESSIONAL ACTIVITIES**

**PROFESSIONAL MEMBERSHIPS**

XXXX-present Name of Society/Academy/Association

**SERVICE TO PROFESSIONAL PUBLICATIONS/EDITORAL WORK**

XXXX-present Journal name, Reviewer

**CONTINUING EDUCATIONAL COURSES ATTENDED**

XXXX Meeting name, City, State

**GRANTS, FUNDING AND PHILANTHROPY**

XXXX ____ Grant Award

Project Name

$Value

**UNIVERSITY AND PUBLIC SERVICE**

XXXX-YYYY ____ Committee, University/Medical Center. Brief description.

**BIBLIOGRAPHY**

**BOOKS AND CHAPTERS**

1. Title

Chapter X: Chapter Name; **Name,** **MD**; Coauthor, MD. Cambridge University Press, Submitted, Pending publication.

**ORIGINAL ARTICLES**

1. **Last Name First Initial.** Title. *Journal abbreviation.* XXXX month; 1(1):V1-V2. doi.org/XYZ
2. Author 1, **Last Name First Initial,** Author 3, Author 4. Title. *Journal abbreviation.* Accepted, pending publication. XXX YYYY. <https://doi.org/XXX>

**PUBLISHED ABSTRACTS**

1. **Last Name First Initial.** Title. *Journal abbreviation.* XXXX month; 1(1):V1-V2. doi.org/XYZ

**ARTICLES ABOUT ME**

1. Title. *Journal abbreviation.* Month, day, YYYY. Link.

**RESEARCH PRESENTATIONS**

**POSTER PRESENTATIONS**

1. **Last Name First Initial**, Author 2, Author 3. Title. Month YYYY. Abstract presented at ____ Conference, City, State.

**ORAL PRESENTATIONS**

1. **Last Name First Initial**, Author 2, Author 3. *Title.* Presented Month YYYY, at ____, City, State.

**TEACHING ACTIVITIES**

**LECTURES – TO FACULTY, RESIDENTS AND MEDICAL STUDENTS**

1. *Title.* Location, Club/Conference/etc. Approximately X residents, Y faculty. Month Day, YYYY.

**LECTURES-NATIONAL MEETINGS AND CME LECTURES**

1. *Title.* **Full Name, MD.** Month Day, YYYY. 30^th^ ____ Conference, Virtual.

**PROFESSIONAL DEVELOPMENT IN TEACHING**

YYYY Course Title

**CLINICAL TEACHING**

I currently work XX hours per month in the ___ Department. I am responsible for supervising residents and medical students during my shifts.

**MENTORSHIP**

1. Name. Month YYYY – Month YYYY. Description of relationship (3-4 sentences)
